# Ecological niche modeling re‐examined: A case study with the Darwin's fox

**DOI:** 10.1002/ece3.4014

**Published:** 2018-04-16

**Authors:** Luis E. Escobar, Huijie Qiao, Javier Cabello, A. Townsend Peterson

**Affiliations:** ^1^ Department of Fish and Wildlife Conservation Virginia Tech Blacksburg VA USA; ^2^ Key Laboratory of Animal Ecology and Conservation Biology Institute of Zoology Chinese Academy of Sciences Beijing China; ^3^ Centro de Conservación de la Biodiversidad Chiloé‐Silvestre Los Lagos Chile; ^4^ Facultad de Medicina Veterinaria Universidad San Sebastián Puerto Montt Chile; ^5^ Biodiversity Institute University of Kansas Lawrence KS USA

**Keywords:** ecological niche modeling, evaluation, *Lycalopex fulvipes*, Maxent, ROC AUC

## Abstract

Many previous studies have attempted to assess ecological niche modeling performance using receiver operating characteristic (ROC) approaches, even though diverse problems with this metric have been pointed out in the literature. We explored different evaluation metrics based on independent testing data using the Darwin's Fox (*Lycalopex fulvipes*) as a detailed case in point. Six ecological niche models (ENMs; generalized linear models, boosted regression trees, Maxent, GARP, multivariable kernel density estimation, and NicheA) were explored and tested using six evaluation metrics (partial ROC, Akaike information criterion, omission rate, cumulative binomial probability), including two novel metrics to quantify model extrapolation versus interpolation (E‐space index I) and extent of extrapolation versus Jaccard similarity (E‐space index II). Different ENMs showed diverse and mixed performance, depending on the evaluation metric used. Because ENMs performed differently according to the evaluation metric employed, model selection should be based on the data available, assumptions necessary, and the particular research question. The typical ROC AUC evaluation approach should be discontinued when only presence data are available, and evaluations in environmental dimensions should be adopted as part of the toolkit of ENM researchers. Our results suggest that selecting Maxent ENM based solely on previous reports of its performance is a questionable practice. Instead, model comparisons, including diverse algorithms and parameterizations, should be the *sine qua non* for every study using ecological niche modeling. ENM evaluations should be developed using metrics that assess desired model characteristics instead of single measurement of fit between model and data. The metrics proposed herein that assess model performance in environmental space (i.e., E‐space indices I and II) may complement current methods for ENM evaluation.

## 1 INTRODUCTION

Ecological niche models (ENMs) have been employed as a predictive tool in diverse research applications, including studies of distributional ecology, biological conservation, climate change effects, evolution, and spatial epidemiology (Peterson et al., 2011). ENM approaches link occurrence data with environmental variables based on a correlative approach to build a representation of a species' ecological requirements. Numerous algorithms have been used to create ENMs, which provide geographic outputs that approximate distributional areas of species (Franklin & Miller, 2009). Ecological niches are manifested in environmental spaces that comprise sets of abiotic variables that shape the species' potential occurrence; niches translate into geographic distributions according to the combined effects of the distribution of the abiotic conditions, biotic interactions, and accessibility by dispersal (Soberón & Peterson, 2005). Even though records of distribution of a species may be abundant, they may be biased, characterizing just a portion of the species' niche, limited by biotic factors (e.g., interspecific competition), dispersal constraints, biased in sampling effort, or simply the existence of sets of conditions on relevant landscapes (Soberón & Peterson, 2005).

Such disjunctions between the fundamental niche and what is observable of it occur when distributional limits are set chiefly or entirely by dispersal considerations, termed the “Wallace's Dream” scenario (Qiao, Soberón, & Peterson, 2015; Saupe et al., 2012). This idea refers to Alfred Russel Wallace, who realized that geographic barriers often limit species to circumscribed regions. The “Wallace's Dream” scenario describes situations in which a species' distributional potential is circumscribed by barriers to dispersal rather than by unsuitable conditions. Take, for instance, the case of the shrub, *Acacia mearnsii*, which was originally restricted by geographic barriers to southeastern Australia and Tasmania. However, this species has a broad potential distribution, and, once introduced to regions beyond the barriers that originally confined it, spread across the Americas, Europe, Asia, Africa, New Zealand, and Pacific and Indian Ocean islands, making it one of the most successful invaders, in light of its capacity to establish populations in new regions worldwide. In Wallaces Dream situations (i.e., species' distributions constrained by dispersal rather than by environmental conditions), ENMs aiming to estimate a species' fundamental niche and in turn its potential distribution lack necessary contrasts for adequate model calibration and, as a consequence, make erroneous conclusions of the species true potential and generally lack predictive ability (Owens et al., 2013; Saupe et al., 2012).

Appropriate evaluation of ENM predictions requires considerable preparation and care: that evaluation samples be independent and that each be representative of the population under study (Hurlbert, 1984). These assumptions are violated when ENMs are evaluated without accounting for spatial autocorrelation and sampling bias implicit in data from real species or when random points are used to replace absence records, which is frequent (Guillera‐Arroita et al., 2015), although often not appreciated. Models evaluated using incorrect metrics and nonindependent data generate incorrect or incomplete results (Lobo, Jiménez‐Valverde, & Real, 2008). Strikingly, robust model assessments are quite rare in ENM, and users too often trust software without understanding or assessing predictive performance (Joppa et al., 2013). Even the important and highly cited work of Elith et al. (2006), which assessed different ENMs based on a large‐scale suite of species and regions, and found that some methods outperformed others, was based on data susceptible to bias (Kadmon, Farber, & Danin, 2004), used biologically unrealistic and mathematically weak evaluation metrics (Lobo et al., 2008; Peterson, Papeş, & Soberón, 2008), and explored only a single feature of model performance (i.e., omission error; see below).

Even today, ENM evaluation methods are limited and restricted to geographic dimensions (Muscarella et al., 2014), even in spite of the fact that ecological niches are manifested in environmental space. The limited availability of metrics for robust model evaluation is alarming given how often ENMs are used to map organisms of high public interest such as agents of infectious diseases, agricultural pests, and endangered species (Peterson et al., 2011). Recent studies suggest that different ENM methods can differ in their performance under diverse circumstances (Qiao et al., 2015), such that no single “best” ENM likely exists, signaling the need for a critical examination of the unquestioning use of particular methods by modelers (e.g., Bhatt et al., 2013). Hence, it is critical to develop new evaluation metrics that can assess diverse characteristics of ENMs, including the amount of interpolation and extrapolation, that is, prediction inside or outside the range of environmental values observed, respectively. In this study, we explored different evaluation metrics using a particular Wallace's Dream case study, the Darwin's Fox (*Lycalopex fulvipes*, Martin 1837), and its likely geographic distribution. We assessed diverse ENM (generalized linear models, boosted regression trees, Maxent, GARP, multivariable kernel density estimation, and NicheA) and evaluation metrics (partial ROC, Akaike information criterion, omission rate, cumulative binomial probability, and E‐space indices I and II) to test how predictive performance may vary or differ in behavior based on the evaluation metric employed.

## 2 METHODS

### 2.1 Case study

We used data from the Darwin's Fox, the only endemic canid of Chile, known from the southern temperate forests along the Pacific coast (Yahnke, 1995). The species was reported by Charles Darwin in 1834 on Chiloé Island and was long considered as an island endemic. However, in 1990, a mainland population was reported at Nahuelbuta National Park to the north, 550 km from the island population (Medel, Jiménez, Jaksić, Yáñez, & Armesto, 1990).

The Darwin's Fox faces important conservation challenges in terms of conflicts with human settlements and dramatic habitat loss (Jiménez, Lucherini, & Novaro, 2008; Sillero‐Zubiri, 2004). A recent survey on Chiloé Island, home to the largest population of the fox (~250 individuals), revealed that >85% of local citizens had negative attitudes toward the fox (Molina‐Espinosa, 2011). The Darwin's Fox is threatened by illegal hunting, apparent competition with other fox species and domestic dogs, lack of ex situ management, and limited knowledge of the basic biology of the species (Baillie, Hilton‐Taylor, & Stuart, 2004; Jaksić, Jiménez, Medel, & Marquet, 1990; Jiménez & Mcmahon, 2004; Sillero‐Zubiri, 2004). With an estimated global population of ~375 individuals and limited knowledge of areas for potential reintroduction, Darwin's Fox is considered to be the canid species at highest risk of extinction globally (Escobar, 2013).

Hence, biological and ecological processes affecting the endangered Darwin's Fox remain poorly understood, complicating efforts to guide field research and policies for its conservation (Sillero‐Zubiri, 2004). The geographic separation of the Chiloé Island and Nahuelbuta populations, due to past (e.g., Chacao Channel dividing Chiloé Island and the mainland) or recent (e.g., extirpation) circumstances, provides an ideal situation in which to test the performance of ecological niche modeling approaches under a situation approximating a “Wallace's Dream” configuration. Thus, ENMs based on this configuration make necessary assessment of diverse evaluation metrics, which can help to identify good candidate models for a Wallace's Dream scenario: In our particular case study, we prioritize ENMs providing high interpolation and low extrapolation to avoid an exaggerated potential range and focus conservation efforts while accounting for the species' observed environmental tolerances.

### 2.2 Occurrence data

**Figure 1:**
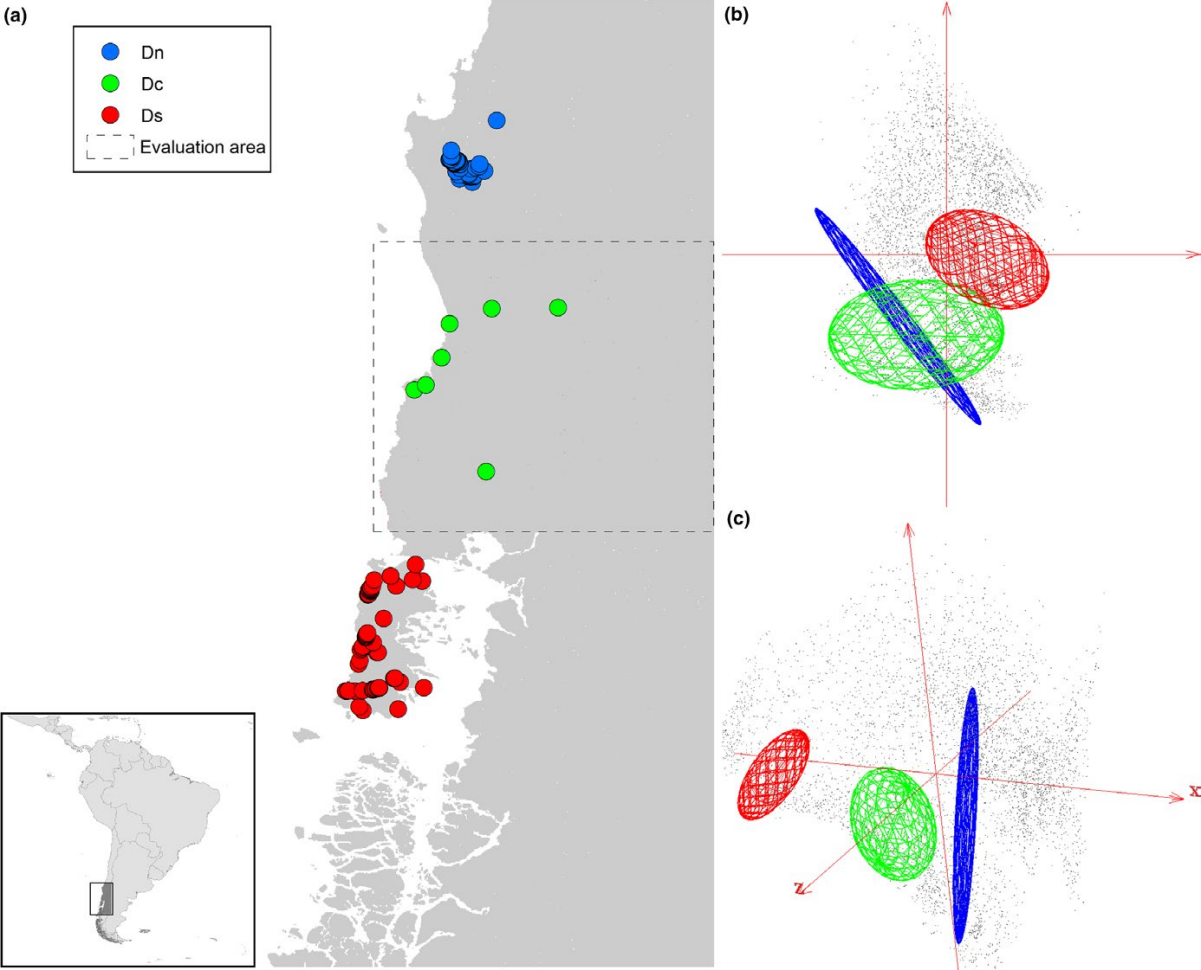
Darwin's Fox (*Lycalopex fulvipes*) occurrences in G and E spaces. (a) Occurrences in geographic space (**G**) in southern Chile (see inset), including a population in Nahuelbuta National Park (**D**_n_) in the northern part of the known species' range (blue points), new records in the central part of the species' distribution (**D**_c_; green points), and the southern populations on Chiloé Island (**D**_s_, red points). The evaluation area, not used in model calibration, is denoted by the dashed line. (b) Occurrences in a two‐dimensional environmental space (**E**), with the same color scheme as in panel A; axes are the first two principal components of the 19 bioclimatic variables, and gray points are the environmental background of the study area. Note the environmental overlap between the blue and green ellipsoids in this bidimensional environmental space (84.4% of the variability in the climatic data). (c) Occurrences in a three‐dimensional environmental space (**E**) with symbolism as in geography. Note that in this higher dimensionality space, no environmental overlap exists between blue and green ellipsoids (94.2% of the variability in the climate data)

Considering the limited knowledge on this species, we collected data on Darwin's Fox occurrences from diverse sources, including the available literature in English, German, and Spanish; natural history museum collections data online; and camera‐trap observations and field observations from our long‐term studies (see summary in Appendix S1). We separated occurrences into three population groups: Nahuelbuta (“northern”), Chiloé Island (“southern”), and new records of the species' occurrence (“central”; Figure 1a). The Nahuelbuta population (47 occurrence sites) was termed **D**_n_, the Chiloé Island population (108 occurrences) **D**_s_, and the new records (7 occurrences) **D**_c_. To reduce pseudoreplication, occurrences were resampled across a 2.5 × 2.5′ grid, which reduced numbers of records to 42 for **D**_n_ and 61 for **D**_s_. Figure 1 shows known occurrences of the species in both geographic (**G**) and environmental (**E**) spaces.

To assess model predictions, we merged **D**_n_ and **D**_s_ as a data set for model calibration, as these populations were recognized much earlier than the intervening populations **D**_c_. In fact, the **D**_c_ occurrences included in this study are here published for the first time in the scientific literature (Appendix S1). **D**_c_ was used for model validation because of its geographic and environmental independence from the **D**_s_ and **D**_n_ populations (i.e., **D**_c_ populations may occur in different areas and under different climatic conditions; Figure 1) and in light of the interest inherent in the question of the relative continuity of populations in between the northern and southern populations.

### 2.3 Study area and environmental variables

The extent of the model calibration area has key impacts on ENM results (Barve et al., 2011). We designed our study area based on the prior knowledge of the species' current distribution across the region 70.5 to 74°W and 38.5 to 41°S, resulting in 18,526 cells (Figure 1a). Specifically, we delimited the study area in the south to include Chiloé Island, in the north to include the Nahuelbuta reserve, to the west by the Pacific Ocean, and to the east by the crest of the Andes Mountains.

Environmental dimensions used to model the species' ecological niche included 19 “bioclimatic” variables with a grid resolution of 2.5 × 2.5′ (Hijmans, Cameron, Parra, Jones, & Jarvis, 2005). Because many of the environmental variables were highly correlated, we developed a principal component analysis (PCA) and retained components sufficient to explain ≥90% of the total variation in the climatic data: The first three principal components accumulated 94.2% of information and were used in analyses.

### 2.4 Ecological niche models

We explored predictive power of six ENMs that can be classified into two functional groups. The first group employs correlational approaches that use occurrences and background data to characterize environmental landscapes. The second group of ENMs uses presence data only in generating model outputs.

Models based on occurrences and environmental background data (background **E**) included the maximum entropy method in Maxent v3.3.3k (Phillips, Anderson, & Schapire, 2006), generalized linear models (GLM; MacCullagh & Nelder, 1989) in BIOMOD2 (Thuiller, Lafourcade, Engler, & Araújo, 2009), boosted regression trees (BRT; Elith, Leathwick, & Hastie, 2008) in dismo (Hijmans, Phillips, Leathwick, & Elith, 2012), and the genetic algorithm for rule‐set production (GARP; Stockwell, 1999) with the best subsets procedure in openModeller 1.5 (de Souza Muñoz et al.*,* 2011). Models in this group generate outputs with continuous values. Models using only presence data included niche‐centroid distance estimation via minimum‐volume ellipsoid approaches in NicheA v.3.0 (Qiao, Peterson, Soberón, Campbell, Ji, & Escobar, 2016) with continuous outputs and hypervolume multivariable kernel density estimation (KDE; Blonder, Lamanna, Violle, & Enquist, 2014) in R (R Core Team 2016) using the package hypervolume (Blonder, 2015) with a binary output. In all cases (i.e., for each of the modeling algorithms), all models were calibrated using default parameters to allow easy replication, fair comparisons with other studies (Elith et al., 2006), and to reproduce customary applications by the broader community of users—however, for illustrative purposes only, a more detailed calibration of Maxent models is described briefly in the Section 4. To facilitate some of the evaluations, all models were set to generate outputs in binary format (i.e., suitable/unsuitable) based on two common threshold values (see below).

Models were calibrated using **D**_n_ and **D**_s_ and then evaluated using **D**_c_. Evaluations were conducted in a two‐dimensional geographic space (i.e., latitude and longitude; **G**) and also in a three‐dimensional environmental space (**E**) defined by the first three components derived from the climate data. During evaluation, we assessed three features of the models: abilities of models to predict correctly the independent **D**_c_ occurrences, the fit of the model with the calibration data, and levels of interpolation and extrapolation.

### 2.5 Model evaluation in geography

#### 2.5.1 Evaluation of continuous models

Models were evaluated based on their spatial fit with the calibration data and on correct prediction of independent evaluation occurrence data across an evaluation area (**D**_c_; Figure 1a, green points and dashed line, respectively). To assess continuous‐output models, we used two metrics: the Akaike information criterion (AIC; Johnson & Omland, 2004; Burnham, Anderson, & Huyvaert, 2011; Warren & Seifert, 2011) and partial area under the receiver operating characteristic curve (pROC; Peterson et al., 2008). Details of our implementation of the two evaluation approaches are in the paragraphs that follow.

AIC is a penalized likelihood criterion expressed as AIC = 2*K − 2*ln(*L*), where *L* is the maximized value of the likelihood function for a model and *K* is the number of parameters employed in the model (Burnham et al., 2011). For Maxent models, *K* is extracted from the “lambdas” file (Warren & Seifert, 2011). GLM were quadratic and without interaction terms and, based on the first three principal components, we set *K *=* *6.

Computing *K* for BRT and GARP is more complex. Generally, the *K* of a classification model (regression tree model or genetic algorithm) is calculated as K=N×(p+(1/2p(p+1))+c), where *N* is the number of nodes on the regression tree (Sain & Carmack, 2002) or the number of individuals per population for a genetic algorithm (Kosakovsky, Mannino, Gravenor, Muse, & Frost, 2007; Yoshimoto, Moriyama, & Harada, 1999), *p* is the dimensionality of the variables, and *c* is the penalty for each data‐based split. Here, we generated 400 populations in GARP model and used 1,000 regression trees in the BRT models; we set *N* to the average number of individuals among the 400 populations and nodes among the 1,000 trees. We used *p *=* *3 because the first three principal components were used as the **E** background. Because every individual in a population or every node on a tree splits the records into three groups (i.e., true, false, and unknown), we set *c* to 3.

Traditional model evaluation approaches (e.g., Elith et al., 2006) involved receiver operating characteristic (ROC) analyses, which have been criticized based on equal weighting of omission and commission errors, consideration of irrelevant predictions, and other issues (Lobo et al., 2008; Peterson et al., 2008); true absences with which to estimate commission error are generally lacking at coarse geographic scales (see Peterson et al., 2011). Hence, we used the alternative pROC metric developed for ENM evaluations (Peterson et al., 2008). This metric assesses the relationship between omission error for independent points and the proportion of area predicted as suitable for the species, but only under conditions of low omission error. AUC ratios (the partial AUC divided by random expectations) range from 0 to 2, with values of 1 representing random performance (Peterson, 2012; Peterson et al., 2008). This evaluation was carried out in pROC software (Barve, 2008) using the continuous output in the evaluation area and evaluation occurrences **D**_c_, with 100 replicate analyses and α = 0.05. A detailed explanation of pROC procedures can be found in Appendix S2.A.

#### 2.5.2 Evaluation of binary models

To compare all six ENMs, including those with binary results only (i.e., KDE), we used omission rate (OR; proportion of evaluation occurrences **D**_c_ predicted incorrectly by binary models), proportion of area predicted suitable in the evaluation area, and cumulative binomial probability (CBP; test based on the omission rate and the proportion of area predicted as suitable in the evaluation area; Escobar et al., 2015; Peterson, 2012). Continuous outputs from Maxent, NicheA, GARP, NicheA, GLM, and BRT were converted to binary based on two thresholding values based on omission error tolerances from the calibration occurrences (Peterson et al., 2011). First, we used a threshold based on the minimum predicted value of all calibration occurrences (**D**_s_ and **D**_n_)—aka minimum training presence, which represents 0% omission error in the calibration occurrences. Additionally, we evaluated models based on a threshold of 5% omission error; that is, we removed the 5% of calibration occurrences, in **D**_s_ and **D**_n_, with the lowest predicted values. OR and CBP allowed us to measure Type I error for every model prediction. To reduce potential Type II error, results of low OR associated with large areas identified as suitable were identified.

### 2.6 Model evaluation in environmental space

**Figure 2:**
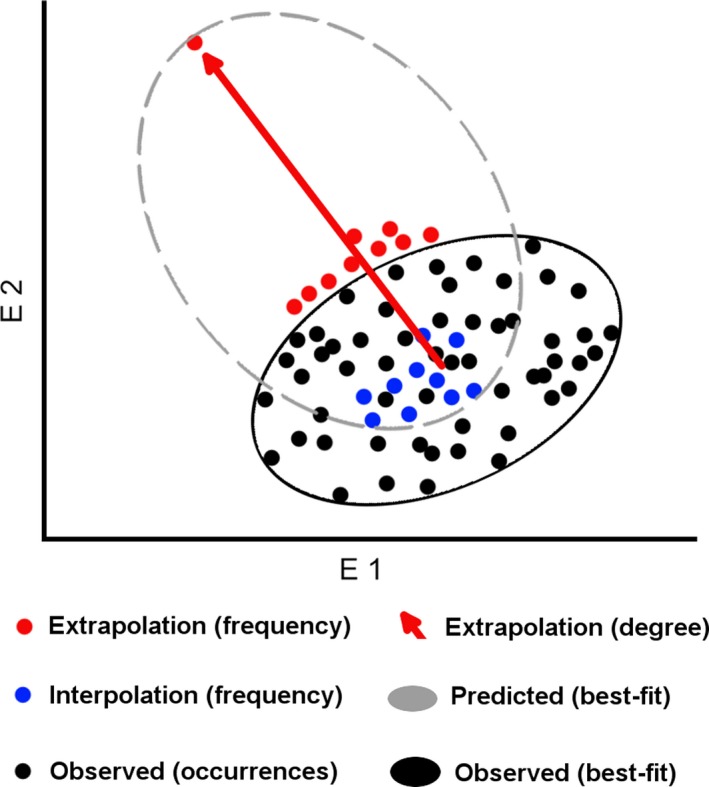
New performance metrics for environmental space. Available occurrences were displayed in a two‐dimensional environmental space (black points) to estimate a minimum‐volume ellipsoid resembling the observed niche (black ellipsoid). *E‐space index I*: Model prediction values were categorized as frequency of interpolation (i.e., number of points predicted inside the observed range; blue points) and frequency of extrapolation (i.e., number of points predicted outside the observed range; red points). *E‐space index II:* This metric compares model's fit with degree of extrapolation. Model fit was measured as the Jaccard index of similarity between the volume of the modeled niche (gray ellipsoid) versus the volume of the observed niche (gray ellipsoid). This metrics also included the degree of extrapolation as the niche distance between the centroid of the observed niche and the most distance prediction of the modeled niche: Note the arrow indicating maximum extrapolation distance between central values of observed data (black points) and most extrapolative values predicted by the model (farthest red point)

We designed two new evaluation metrics that are applied in environmental dimensions: E‐space indices I and II. E‐space index I assesses the amount of environmental interpolation and extrapolation in predictions: Environmental interpolation is prediction of environmental values that are within the range of environmental values of the occurrences used for model calibration (blue points in Figure 2). In contrast, extrapolation refers to prediction of environmental values beyond the range of values represented among the occurrence data (red points in Figure 2). We used all available occurrences (**D**_s_, **D**_n_, **D**_c_; black points in Figure 2) to estimate a best‐fit (minimal volume) ellipsoid (MVE; black ellipsoid in Figure 2) in a three‐dimensional environmental space (Van Aelst & Rousseeuw, 2009; details in Appendix S2.B) composed of the first three principal components. This MVE was then used as the observed niche with which interpolation and extrapolation were evaluated. Analyses in E‐space were carried out based on unique environmental combinations; thus, we ignored duplicate points with identical values in environmental space. We estimated frequency of extrapolation as the number of unique environmental values predicted outside of the ellipsoid. Interpolation was the number of unique environmental combinations predicted inside the ellipsoid (Appendix S2.B). This approach allowed us to discriminate between failed models (i.e., high extrapolation, low interpolation) and successful models (i.e., low extrapolation, high interpolation), according to our criteria of biological realism (Owens et al., 2013; Peterson et al., 2011).

E‐space index II is a more complex metric related to the level or intensity of extrapolation and the fit of the model with the data available. For example, two ENMs might have similar frequencies or amounts of extrapolation (e.g., both models with 10 values predicted inside of the observed range of 10–20°C, and 10 predicted outside; see above E‐space index I), but the degree of extrapolation between these models could be dramatically different, making models biologically incompatible (e.g., extrapolation to values of 21°C vs. 100°C; Figure 2). Thus, because a metric measuring this *degree* of extrapolation in terms of the observed values (i.e., distance of points from the MVE) would be useful, we measured the degree of extrapolation as the Euclidian distance between the centroid and the most distant prediction away from the MVE (Figure 2; Appendix S2.B). To assess overall fit of the model to the data in environmental space, we also measured similarity between the observed niche (i.e., MVE) and niches predicted by the different models using the Jaccard similarity index (Jaccard, 1912; Figure 2; Appendix S2.B). Finally, once models were evaluated with independent data, final predictions were developed using all the occurrences available for more informed models of the species truly potential. Binary models of suitability were generated using the threshold based on 0% omission error described above, and binary maps were summed to generate an ensemble summarizing areas with agreement among models.

## 3 RESULTS

The environmental space defined by the first three principal components explained 94.2% of overall variance; the first two dimensions explained 84.4%. Interestingly, the **D**_c_ occurrences overlapped environmentally with northern populations **D**_n_ from a 2D environmental perspective (Figure 1b), but environmental overlap between **D**_c_ and **D**_n_ was nil in the 3D space (Figure 1c). Darwin's Fox populations in the northern and southern areas of the range provided distinct environmental information to the models (i.e., no environmental overlap manifested between **D**_s_ and **D**_n_). **D**_c_ occurrences filled a portion of the environmental gap between **D**_s_ + **D**_n_, and occupied the broadest environmental space (green ellipsoid in Figure 1b,c). **D**_c_ niche volume was 5.56, compared to **D**_n_ and **D**_s_ volumes of 2.87 and 0.54, respectively. We found heterogeneous frequencies of occurrences across the species' temperature range: When mean temperature was extracted using all available occurrences, Darwin's Foxes were not found across the entire temperature range available in the study area, but rather clustered in specific temperature intervals (Appendix S3.A).

**Figure 3:**
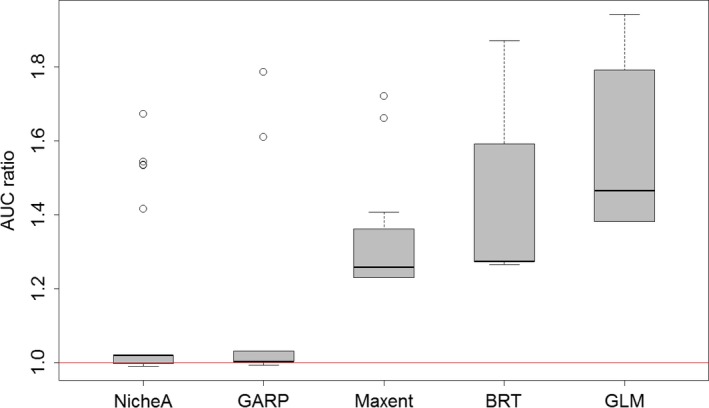
Ecological niche model evaluation in central populations (**D**_c_) using partial ROC ratios based on northern and southern (**D**_s_ + **D**_n_) populations. Boxplots denote AUC ratios in 100 replicates using 50% of evaluation occurrences in each replicate and 5% of omission error. The red line denotes a null distribution of AUC ratios under which predictions are not better than by random expectations. GLM, generalized linear model; BRT, boosted regression trees; Maxent, maximum entropy; GARP, genetic algorithm for rule‐set production; KDE, hypervolume multivariable kernel density estimation; NicheA, minimum‐volume ellipsoid

**Figure 4:**
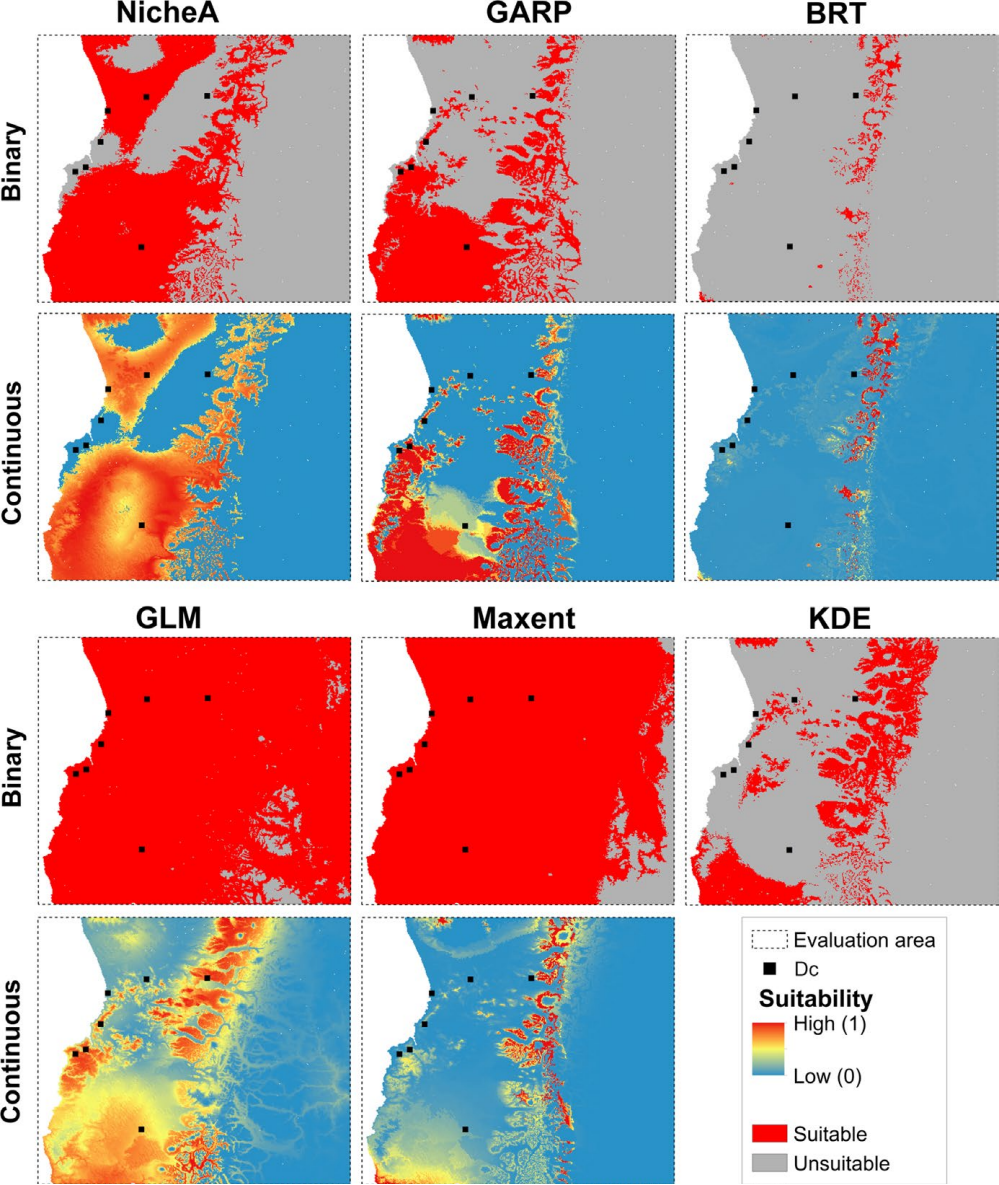
Continuous and binary models of Darwin's Fox in the evaluation area (threshold = 0%). Models calibrated using northern and southern (**D**_s_ + **D**_n_) Darwin's Fox occurrences, projected in the evaluation area (dashed line). Independent occurrences from the central population (**D**_c_; black squares) are used to evaluate the model in terms of predictions in continuous (i.e., range of colors; highly suitable = red, unsuitable = blue) and binary outputs (i.e., suitable = red, unsuitable = gray). Binary models were generated based on 0% omission error from calibration occurrences. NicheA, minimum‐volume ellipsoid; GARP, genetic algorithm for rule‐set production; BRT, boosted regression trees; GLM, generalized linear model; Maxent, maximum entropy; KDE, hypervolume multivariable kernel density estimation

**Figure 5:**
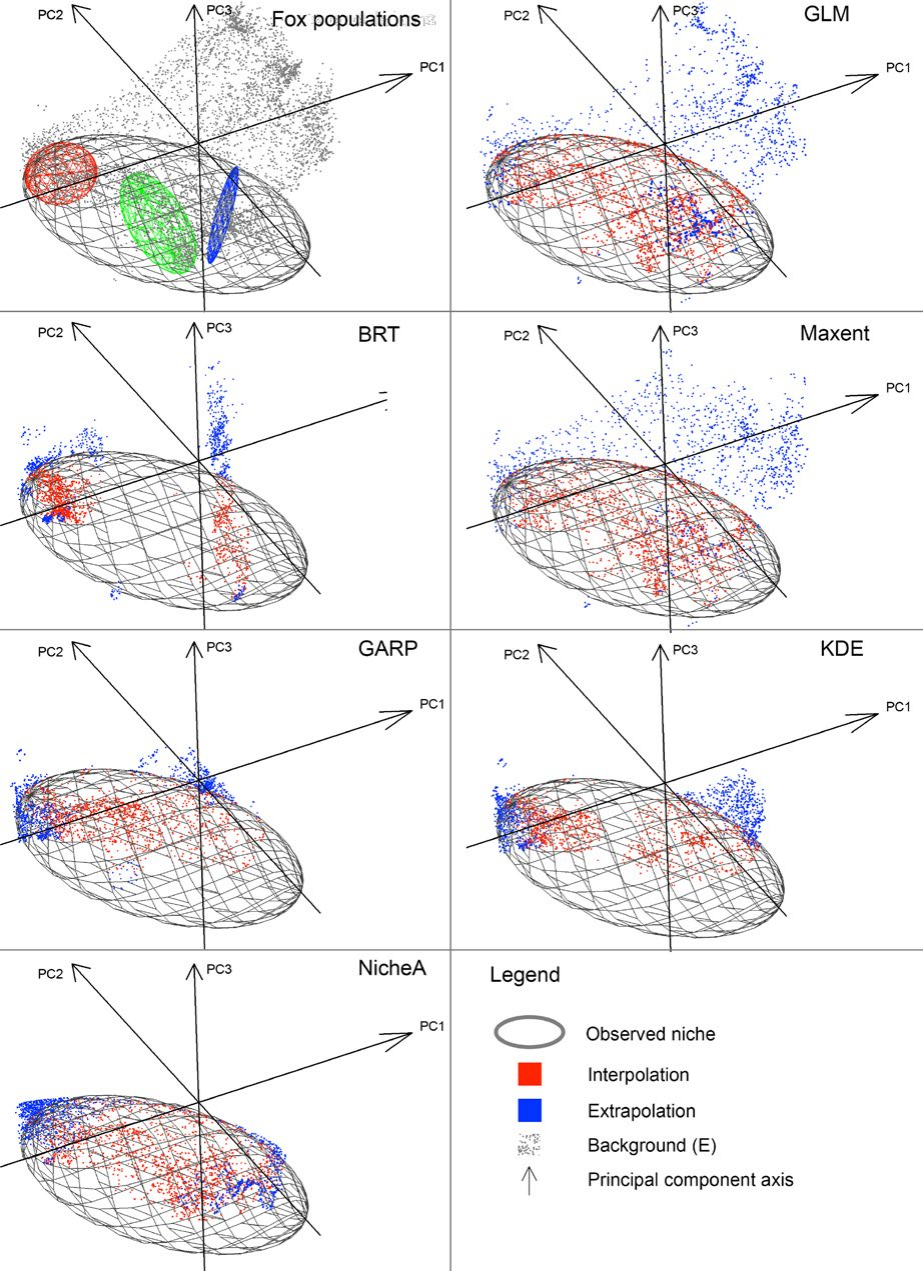
Model evaluations based on interpolation and extrapolation in environmental space (threshold = 0%). Top left: Darwin's Fox populations, from the northern (blue ellipsoid), central (green ellipsoid), and southern (red ellipsoid); populations were enclosed to generate observed ecological niche hypotheses; the environmental background is shown in this panel as gray points. Subsequent panels: Predictions were categorized according to environmental interpolation (red points) as predictions inside the ellipsoid and environmental extrapolation (blue points; see Section 2) as predictions outside the ellipsoid; the environmental background is not shown in these panels for better visualization of models output. GLM, generalized linear model; BRT, boosted regression trees; Maxent, maximum entropy; GARP, genetic algorithm for rule‐set production; KDE, hypervolume multivariable kernel density estimation; NicheA, minimum‐volume ellipsoid. Note that predictions by some models resemble the background cloud (e.g., GLM and Maxent), suggesting that all the conditions available in the model calibration area were predicted suitable by the model via model interpolation (red points) or extrapolation (blue points)

**Figure 6:**
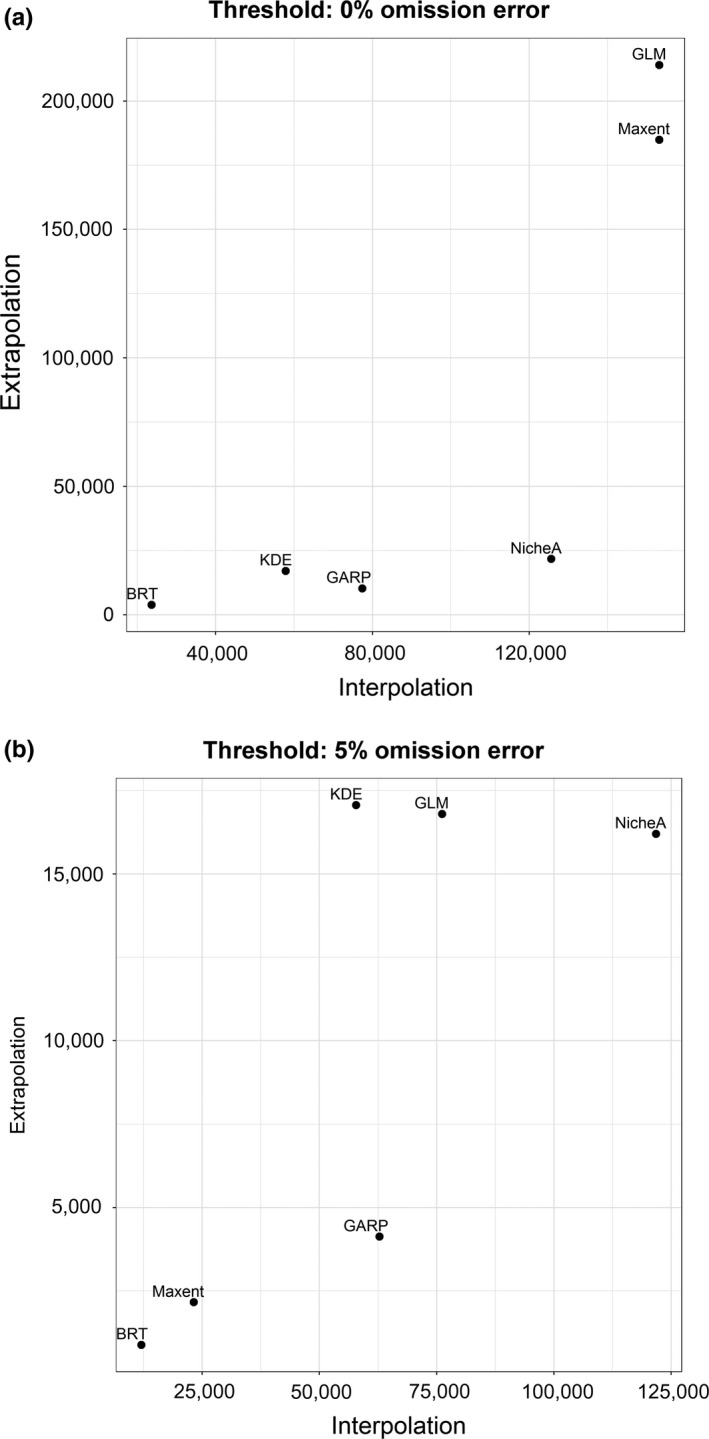
Model performance evaluation in environmental dimensions based on the *E‐space index I*. Frequency of environmental interpolation and extrapolation. Models were plotted in terms of environmental interpolation (*x*‐axis) and extrapolation (*y‐*axis) to compare their performance under both circumstances. Units are number of environmental points from principal component variables with predicted values inside (i.e., interpolation) or outside the ellipsoid of the observed niche (i.e., model extrapolation). (a) Binary models based on a threshold of 0% omission error in the calibration occurrences. (b) Binary models based on a threshold of 5% omission error in the calibration occurrences. GLM, generalized linear model; BRT, boosted regression trees; Maxent, maximum entropy; GARP, genetic algorithm for rule‐set production; KDE, hypervolume multivariable kernel density estimation; NicheA, minimum‐volume ellipsoid

**Figure 7:**
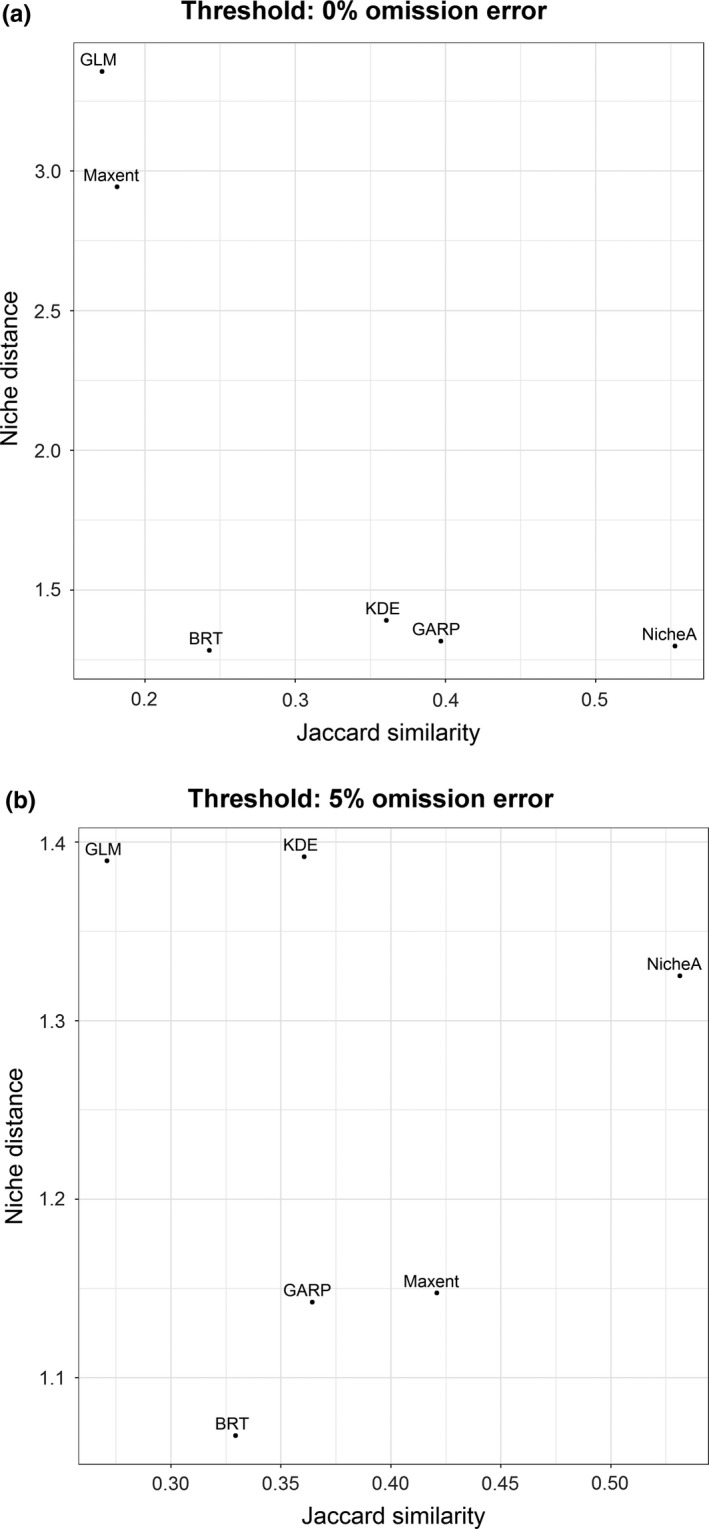
Model performance evaluation in environmental dimensions based on the *E‐space index II*. Model fit in terms of niche overlap measured by Jaccard similarity and the degree of extrapolation as the niche distance between the occurrences and niche center. Models were plotted in terms of Jaccard similarity (*x*‐axis) and degree of extrapolation in terms niche distance (*y*‐axis; see Figure 2). (a) Binary models based on a threshold of 0% omission error in the calibration occurrences. (b) Binary models based on a threshold of 5% omission error in the calibration occurrences. GLM, generalized linear model; BRT, boosted regression trees; Maxent, maximum entropy; GARP, genetic algorithm for rule‐set production; KDE, hypervolume multivariable kernel density estimation; NicheA, minimum‐volume ellipsoid

Our analyses focused on the predictive capabilities of models calibrated based on incomplete data (i.e., using only **D**_n_ and **D**_s_; Figures 3, 4, 5, 6, 7). According to AIC values from models with incomplete data (Table 1), the default parameter Maxent model had the best fit, followed by GLM. However, when all occurrences were employed (**D**_s_, **D**_n_, **D**_c_) to calibrate final models, GLM provided the best fit to the data based on AIC.

**Table 1:** Akaike information criterion (AIC) values for models calibrated using northern and southern populations (i.e., **D**_n_ + **D**_s_) and all occurrences available (**D**_n_ + **D**_c_ + **D**_s_)

	*K*	Ln(likelihood)	AIC
Calibration models **D**_n_ + **D**_s_			
Maxent	57	−1061.09	2236.17
GLM	6	−1206.42	2424.85
BRT	192	−1031.91	2447.82
GARP	96	−1132.65	2457.30
Final models **D**_n_ + **D**_c_ + **D**_s_			
GLM	6	−1187.17	2386.34
Maxent	55	−1173.17	2456.33
GARP	96	−1155.53	2503.06
BRT	192	−1133.39	2650.78

Using independent evaluation data (**D**_c_), the models' pROC ratios provided detailed quantitative information on model outputs, allowing us to detect inconsistent models (i.e., high variation; e.g., GLM, BRT; Figure 3). However, pROC showed low discrimination ranking high models with very broad (e.g., GLM) and very narrow predictions (e.g., BRT; Figures 3 and 4). On average, based on pROC, GLM, BRT, and Maxent had good predictive capabilities compared to random models, whereas NicheA and GARP had low performance in predicting independent evaluation data (Figures 3 and 4).

Threshold values based on 0% and 5% omission error changed considerably for GLM, BRT, and GARP (Appendix S3.B). OR evaluations with and omission error threshold of 0% revealed that GLM and Maxent predictions anticipated all independent evaluation points (i.e., zero omission error; Table 2), but by predicting extensive areas as suitable (Figure 4). On the other hand, BRT identified only 3% and KDE 21% of the evaluation area as suitable, but were unable to anticipate any of the evaluation occurrences **D**_c_ (Figure 4; Table 2). NicheA and GARP identified narrower areas as suitable (41.2% and 28.3%, respectively), but failed to anticipate four of seven occurrences, predictions that were not better than chance according to the CBP test. Hence, based on the CBP metric with a threshold of 0% omission error, only GLM and Maxent provided better‐than‐random predictions (Table 2). When models where thresholded based on 5% omission error, GLM and Maxent dramatically reduced the area predicted suitable (Table 2; Appendix S3.C), which could be associated with the positive skew distribution of their predicted values (Appendix S3.B). For example, most Maxent predictions had low values, so that small increments in the threshold will assign broad areas as unsuitable. Under this threshold, only GLM provided predicted better than by chance based on CBP metric (Table 2).

**Table 2:** True omission error evaluations based on validation data from the novel population **D**_c_ (*n* = 7). Binary maps based on an a priori percentage of omission error tolerance of 0% and 5% in the calibration data **D**_n_ and **D**_s_

Model calibrated using **D**_n_ + **D**_s_	Omission rate		Area predicted suitable		CBP (*p* value)	
	0%	5%	0%	5%	0%	5%
GLM	0.00	0.30	0.96	0.30	<.001	<.001
Maxent	0.00	1.00	0.92	0.03	<.001	>.05
BRT	1.00	1.00	0.03	0.01	>.05	>.05
KDE	1.00	NA	0.21	NA	>.05	NA
NicheA	0.57	0.57	0.41	0.40	>.05	>.05
GARP	0.57	0.71	0.28	00.20	>.05	>.05

CBP, Cumulative binomial probability; GLM, generalized linear model; BRT, boosted regression trees; Maxent, maximum entropy; GARP, genetic algorithm for rule‐set production; KDE, hypervolume multivariable kernel density estimation; NicheA, minimum‐volume ellipsoid.

Metrics of ENM performance in environmental space showed that different models predicted different levels of interpolation and extrapolation, information not captured by traditional metrics (e.g., AIC; Figure 5 and Appendix S3.D). Based on E‐space index I, ENMs showed different frequency of interpolation and extrapolation (Figure 6). Based on a threshold of 0% omission error, models with highest frequency of environmental extrapolation were GLM and Maxent (Figure 6a); BRT had the lowest amount of extrapolation. NicheA showed high amounts of environmental interpolation, similar to Maxent, but much lower environmental extrapolation, similar to BRT. GARP, followed by KDE, had intermediate amounts of interpolation and low extrapolation in predictions, suggesting that these models balanced interpolation and extrapolation better (Figure 6a). However, even though GARP and KDE were similar in interpolation and extrapolation frequencies, they predicted suitability under different environmental values and extents, as visualized in environmental space (Figure 6a). E‐space index I also showed that some ENMs are more affected than others by the threshold value selected. For example, while BRT and GLM remained consistent, Maxent was the most affected by variations in threshold values, with a radical change from very high interpolation and extrapolation to very low interpolation and extrapolation (Figure 6a vs. b).

When E‐space index II was considered, we found that under a 0% omission threshold, GLM and Maxent predicted suitability in environmental conditions considerably beyond observed values (Figure 7a). GLM and Maxent models also showed the lowest environmental overlap with observed occurrences, revealing high extrapolation and low fit to observations when environmental dimensions are considered. NicheA provided the best fit between predictions and observations as measured with Jaccard similarity; NicheA and BRT showed the lowest degree of extrapolation. KDE and GARP showed moderate similarity between predictions and observations and low degree of extrapolation as expressed as niche distance (Figure 7b). Using a 5% omission error threshold reduced considerably the exaggerated degree of extrapolation for GLM and Maxent as expressed by distance of outlier predictions in environmental space (Figure 7b; Appendix S3.D).

In geographic space and using 0% omission error threshold, models with high extrapolation (e.g., GLM, Maxent) were visualized as broad areas of potential distribution for Darwin's Fox. GLM and Maxent were considerably impacted geographically by increments in the omission error threshold to 5%, shrinking dramatically the area predicted suitable (Appendix S3.C). ENMs with limited extrapolation and interpolation (e.g., BRT, KDE, GARP) identified more specific areas as suitable even when calibrated with all available occurrences (Figure 4 and Appendix S3.E). Summing all of the binary models calibrated using all available occurrences for the final ENM ensemble approach showed areas of high and low agreement of models, mostly clustered in areas of known occurrence, and extending across the Andean mountain valleys and onto the plateau (Appendix S3.E).

## 4 DISCUSSION

Many previous studies have attempted to assess predictive performance of ecological niche modeling methods regarding their predictive ability, overfitting, and accuracy (Rangel & Loyola, 2012). Others have argued that a model ensemble approach is a parsimonious way to deal with ENM‐based variation, avoiding the decision of choosing one ENM over another (Araújo & New, 2007); however, using a single model is the common practice (Qiao et al., 2015). For instance, the work of Elith et al. (2006) has seen over 5,200 citations, but its conclusions are rarely questioned, even given known weaknesses in the evaluation metrics used (Golicher, Ford, Cayuela, & Newton, 2012; Lobo et al., 2008; Peterson, Papeş, & Eaton, 2007; Peterson et al., 2008). In this study, we have begun to address key issues that have been generally neglected in selecting ENMs for studies (but see Diniz‐Filho et al., 2009; Buisson, Thuiller, Casajus, Lek, & Grenouillet, 2010; Terrible et al., 2012; de Oliveira, Araújo, Rangel, Alagador, & Diniz‐Filho, 2012; de Oliveira, Rangel, Lima‐Ribeiro, Terribile, & Diniz‐Filho, 2014; de Oliveira et al., 2015; Collevatti et al., 2012, 2013). Model performance varied dramatically among ENMs and depending on the evaluation metric employed, making multimetric comparisons and careful consideration of the needs of each particular study a critical element in the analytical process and final model selection.

Under ideal conditions, species will occupy a continuous portion of environmental space that reflects their fundamental ecological niches (Soberón & Nakamura, 2009). For most species, however, such conditions rarely exist, and Wallace's Dream scenarios may appear, in which the true dimensions of the niche that are observable are limited due to other factors. Here, the historical distribution of the Darwin's Fox is an ideal example of a Wallace's Dream scenario, with isolated populations occupying different environmental spaces. Novel environmental values representing previously unknown sectors of the species' fundamental ecological niche were illuminated using novel reports of Darwin's Fox populations in the central parts of the species' range (**D**_c_). This new information effectively filled both environmental and geographic gaps between northern and southern populations (Figure 1). We also found that some ENMs may fail to reconstruct species' niches under Wallace's Dream scenarios, which was manifested by models' inability to predict **D**_c_ populations.

GLM filled consistently and substantially the environmental gap in the observable parts of the species' ecological niche (Figures 4 and 5), but at the cost of dramatic extrapolation beyond the environmental conditions occupied by the species (Figures 5 and 6). High model extrapolation may be undesirable, as it assumes that the species can survive under conditions outside of the range of conditions under which it has been observed to maintain populations, sometimes far outside known conditions. Such extrapolation can be biologically unrealistic; for example, in some cases, models anticipate suitability at 100°C, which is implausible for most species (Owens et al., 2013).

On the other hand, model overfitting, expressed in our environmental space metrics as low interpolation and extrapolation (e.g., BRT), is also likely to be biologically unrealistic. For example, why would a species not be able to survive under intermediate conditions otherwise contained inside its environmental range (e.g., see Appendix S3.A)? Basic physiology suggests that species will be able to survive under intermediate conditions, as physiological responses tend to be bell‐shaped in terms of response of suitability to environmental conditions, rather than bimodal, intolerance to intermediate environmental conditions (Austin, Cunningham, & Fleming, 1984; Birch, 1953; Maguire, 1973; Qiao, Escobar, et al., 2016).

Under this thinking framework, we would seek an ENM method with high interpolation but low extrapolation, at least for the needs of this study. Some methods performed poorly under some evaluation metrics (e.g., AIC), but may fulfill the requirements of this study, such as NicheA, GARP, and KDE. We suggest that the evaluation metric should be selected based on the model feature desired and the use intended (Soberón & Peterson, 2005). For example, some research questions may require prioritizing model overfitting expressed as low interpolation and low extrapolation, whereas others may require predictions that are not overfitted and that are inclusive of broad suitable conditions. For example, an overfit model would be desirable in cases attempting to identify suitable areas for reintroductions of rare species, while broad models may be desirable for searches for last populations of possibly extinct species. Hence, assessing modeling methods in terms of diverse metrics should be a common practice in view of the abilities of different metrics to assess different model features. In this vein, AIC corrected by sample sizes (AICc) could be included in the set of evaluation metrics for ENMs developed from small number of occurrences (Warren & Seifert, 2011). We also noted that our small number of occurrences affected dramatically the thresholding. For example, removing 5% of calibration occurrences with the lowest predicted values, instead of 0%, resulted in Maxent models that were markedly different.

Detailed parameterizations instead of default configurations may impact fit of models to the data in interesting ways (de Oliveira et al., 2017). In particular, selection of Maxent models based on information criteria such as AIC is emerging as a popular new paradigm in ecological niche modeling (Warren & Seifert, 2011). This practice, however, is still under intense exploration and experimentation (Muscarella et al., 2014), and the biological significance of such “best” models remains understudied. We recall the words of Samuel Karlin, an American mathematician who stated, “The purpose of models is not to fit the data but to sharpen the questions.” As regards the present study, our focus was on developing useful evaluation metrics, rather than on detailed parameterization of models, which have been treated elsewhere (Muscarella et al., 2014; Peterson et al., 2011).

Still, as many readers will be curious about the effects of detailed parameterization on the sort of results that we have presented in this contribution, we explored a more detailed parameterization of Maxent (Appendix S4). We assessed 1,220 candidate models and found that the optimal AICc metrics do not coincide with default parameters. That is, detailed model parameterization helped to generate models with better fit with the data and less complexity (57 parameters for the default model vs. 23 for the optimized model). In terms of other metrics, however, default and optimized models did not differ markedly (e.g., omission rate, area predicted suitable, cumulative binomial probability), such that the resulting distribution maps did not differ noticeably (Appendix S4).

Based on diverse model evaluation criteria (amount of extrapolation, amount of interpolation, degree of extrapolation, model fit with the data, pROC, cumulative binomial test, and omission rate), we found that no single ENM achieved the highest scores in all metrics. For the particular application examined in this study, we preferred models presenting low extrapolation and high interpolation, criteria that were chosen a priori. Under this condition, NicheA was a good candidate model in terms of low extrapolation, high interpolation, moderate omission rate, high similarity to the observed niche, and low degree of extrapolation, but at the cost of a non‐significant *p*‐value as estimated based on a one‐tailed binomial test. For an expanded discussion of the results on the distributional ecology of Darwin's Fox, see Appendix S3.E.

Ecological niche models are usually designed and assessed from a geographic perspective (e.g., Radosavljevic & Anderson, 2014). However, our results suggest that such interpretations hold relatively limited information and should be taken with caution; models should rather be analyzed in both environmental and geographic spaces. What is more, complications arising from spatial autocorrelation and nonindependence of points in geographic spaces further complicate geographic only evaluations. More highly dimensional environmental spaces may be still more informative in such explorations (Figure 1).

We encourage a future reanalysis of the original work of Elith et al. (2006), based on the same data sets and model outputs, but under different and diverse evaluation metrics. Such a reanalysis would determine whether that study's conclusions are consistent under the same assumptions and parameters, but in the context of different evaluation metrics. Such a re‐evaluation exercise would increase the transparency and good practices behind one of the foundational studies in ecological niche modeling. We note that ENMs in this study used default parameters to allow replicability and fair comparisons, but more detailed parameterizations may generate different outputs. To facilitate the replication of our study, we have incorporated the scripts of the metrics as Appendix S2.B. However, development of a formal software package including ENM evaluations in environmental space is warranted.

## 5 CONCLUSIONS

AUC and AIC have dominated protocols for ENM model evaluation for at least a decade; however, such metrics are limited in information and may fail to evaluate some properties of the desired model (Qiao et al., 2015). Researchers should establish clear and delimited a priori assumptions and desired model characteristics (Peterson, 2006); based on these decisions, researchers can select ENM methods and evaluation metrics that address their requirements. We found that model evaluations in environmental dimensions were highly informative to guide model selection and interpretation. Our proposed E‐space metrics of extrapolation and interpolation in the environmental space offer a useful enrichment to more customary characterization of model predictions. Future research on these metrics should include development of standardized indices to make studies comparable.

## CONFLICT OF INTERESTS

The authors declare no competing financial interests.

## AUTHOR CONTRIBUTIONS

L.E.E. conceived the study. H.Q. and L.E.E. performed the analyses and wrote the manuscript. H.Q. developed the evaluation metrics. J.C. collected data and wrote the manuscript. A.T.P. co‐wrote the manuscript. All authors reviewed the manuscript.
