# Supplementary material for: Ecological niche modeling re‐examined: A case study with the Darwin's fox
Source: Ecol Evol. 2018 Apr 16;8(10):4757–70. doi: 10.1002/ece3.4014 (PMC5980497; doi:10.1002/ece3.4014)
Supplement: Supplementary file 1 [file ECE3-8-4757-s001.pdf]

Ecological Niche Modeling Re-examined: A Case Study with the Darwin's Fox

Luis E. Escobar[1], Huijie Qiao[2*], Javier Cabello[3,4], A. Townsend Peterson[5]

[1]Department of Fish and Wildlife Conservation, Virginia Tech, Blacksburg, Virginia, 24061 USA.
[2]Key Laboratory of Animal Ecology and Conservation Biology, Institute of Zoology, Chinese Academy of Sciences, Beijing, 100101 China.
[3]Centro de Conservación de la Biodiversidad Chiloé-Silvestre, Chiloé Island, Los Lagos, Chile.
[4]Facultad de Medicina Veterinaria, Universidad San Sebastián, Campus Pichi Pelluco, Lago Panguipulli, 1390 Puerto Montt, Chile.
[5]Biodiversity Institute, University of Kansas, Lawrence, Kansas, 66045 USA.

* Corresponding author: Huijie Qiao, qiaohj@ioz.ac.cn

Table S1. Darwin's fox (*Pseudalopex fulvipes*) occurrence points. Occurrences are in geographic coordinates. Data were captured from literature and our ongoing field work in southern Chile in areas including, Río Inio; Caleta Emerenciana, Pirámide, Cerro Mirador, Chaiguaco, and Yaldad in Tantauco Park, Río Chaigua, Chaiguate in Chaiguaco; Rahue; Rancho Grande, Lar, Pescado River Chiloé National Park; Huelde; Cucao; Tulo bridge Ahuenco; Dunas Tongoy; Guaibil-Ahuenco; Sector Palomar-Choroihué; Mechaico-San Antonio; San Antonio.

Longitude	Latitude	Source
-73.75	-43.3333	(Jiménez and McMahon, 2004)
-73.000	-37.750	(Jiménez and McMahon, 2004)
-72.9843	-37.887	(Vasquez-Grandon, 2011)
-72.9995	-37.8309	(Vasquez-Grandon, 2011)
-73.02	-37.826	(Vasquez-Grandon, 2011)
-72.9812	-37.8255	(Vasquez-Grandon, 2011)
-73.012	-37.8248	(Vasquez-Grandon, 2011)
-72.9968	-37.8228	(Vasquez-Grandon, 2011)
-73.0402	-37.8208	(Vasquez-Grandon, 2011)
-72.9633	-37.8167	(Vasquez-Grandon, 2011)
-73.0373	-37.8154	(Vasquez-Grandon, 2011)
-73.1228	-37.8118	(Vasquez-Grandon, 2011)
-73.0198	-37.811	(Vasquez-Grandon, 2011)
-73.0224	-37.8004	(Vasquez-Grandon, 2011)
-73.1514	-37.7765	(Vasquez-Grandon, 2011)
-73.1479	-37.775	(Vasquez-Grandon, 2011)
-72.8573	-37.7704	(Vasquez-Grandon, 2011)

-73.1104	-37.7414	(Vasquez-Grandon, 2011)
-72.9497	-37.7358	(Vasquez-Grandon, 2011)
-72.9141	-37.7254	(Vasquez-Grandon, 2011)
-73.1148	-37.704	(Vasquez-Grandon, 2011)
-73.115	-37.7008	(Vasquez-Grandon, 2011)
-72.9107	-37.6994	(Vasquez-Grandon, 2011)
-73.1732	-37.6978	(Vasquez-Grandon, 2011)
-73.1646	-37.6955	(Vasquez-Grandon, 2011)
-73.1753	-37.6909	(Vasquez-Grandon, 2011)
-73.1762	-37.6872	(Vasquez-Grandon, 2011)
-73.1489	-37.685	(Vasquez-Grandon, 2011)
-73.1742	-37.6828	(Vasquez-Grandon, 2011)
-73.1212	-37.6822	(Vasquez-Grandon, 2011)
-73.1775	-37.6773	(Vasquez-Grandon, 2011)
-73.1254	-37.6693	(Vasquez-Grandon, 2011)
-73.1351	-37.6654	(Vasquez-Grandon, 2011)
-73.1406	-37.662	(Vasquez-Grandon, 2011)
-73.1454	-37.6606	(Vasquez-Grandon, 2011)
-73.151	-37.6604	(Vasquez-Grandon, 2011)
-73.2223	-37.6534	(Vasquez-Grandon, 2011)
-73.2151	-37.6501	(Vasquez-Grandon, 2011)
-73.2081	-37.6496	(Vasquez-Grandon, 2011)
-73.2005	-37.6492	(Vasquez-Grandon, 2011)
-73.1848	-37.6249	(Vasquez-Grandon, 2011)
-73.1953	-37.6231	(Vasquez-Grandon, 2011)
-73.2019	-37.6203	(Vasquez-Grandon, 2011)
-73.203	-37.558	(Vasquez-Grandon, 2011)
-73.4814	-43.1134	(Jiménez et al., 2012)
-73.731	-43.0545	(Jiménez et al., 2012)
-74.1586	-42.8688	(Jiménez et al., 2012)
-74.1474	-42.8324	(Jiménez et al., 2012)
-73.9736	-42.7501	(Jiménez et al., 2012)
-74.1402	-42.7226	(Jiménez et al., 2012)
-74.0092	-42.6458	(Jiménez et al., 2012)
-74.0343	-42.0918	(Jiménez et al., 2012)
-74.0158	-42.0609	(Jiménez et al., 2012)
-73.6017	-41.9973	(Jiménez et al., 2012)
-73.571	-41.8411	(Jiménez et al., 2012)
-73.9577	-42.7477	(González-Acuña et al., 2006)
-74.1222	-42.7094	(González-Acuña et al., 2006)
-73.5036	-42.0117	(González-Acuña et al., 2006)
-73.9578	-42.7478	(Killian and Kristina Killian, 2005)

-74.1222	-42.7094	(Killian and Kristina Killian, 2005)
-73.5036	-42.0118	(Killian and Kristina Killian, 2005)
-73.900	-42.400	(Jimenez et al., 1991)
-72.7333	-37.25	(Jimenez et al., 1991)
-72.8419	-40.8808	(Dieguez, 2014)
-73.5833	-40.0333	(Farias et al., 2014)
-73.4667	-39.9833	(Farias et al., 2014)
-73.3	-39.7	(Farias et al., 2014)
-73.2333	-39.35	(Vilà et al., 2004)
-73.1167	-37.85	(Vilà et al., 2004)
-72.1	-39.1833	(D'Elía et al., 2013)
-74.1167	-43.35	Field Museum (www.gbif.org)
-74.1531	-43.3103	This study
-74.1263	-43.1487	This study
-74.1258	-43.148	This study
-74.285	-43.1479	This study
-74.2811	-43.1478	This study
-74.2917	-43.1473	This study
-74.2769	-43.1473	This study
-74.125	-43.1466	This study
-74.1993	-43.146	This study
-74.1181	-43.145	This study
-74.2659	-43.1448	This study
-74.1177	-43.1447	This study
-74.013	-43.1363	This study
-74.0126	-43.1356	This study
-74.0086	-43.1348	This study
-73.9959	-43.1315	This study
-74.0101	-43.1309	This study
-74.0089	-43.1296	This study
-74.0079	-43.1292	This study
-73.991	-43.1289	This study
-73.9808	-43.1232	This study
-73.9599	-43.1209	This study
-73.9559	-43.1199	This study
-73.9469	-43.1175	This study
-73.9528	-43.1175	This study
-73.9454	-43.1173	This study
-73.9515	-43.1165	This study
-73.9493	-43.1153	This study
-73.795	-43.0183	This study

-73.7851	-43.0177	This study
-73.7895	-43.0176	This study
-73.7891	-43.0171	This study
-73.786	-43.0161	This study
-74.1199	-42.6909	This study
-74.1093	-42.6903	This study
-74.1156	-42.6887	This study
-74.119	-42.6881	This study
-74.0842	-42.5967	This study
-74.0869	-42.5845	This study
-74.0861	-42.5807	This study
-74.085	-42.5797	This study
-74.0794	-42.5797	This study
-74.0798	-42.5778	This study
-74.0732	-42.5697	This study
-74.0774	-42.5624	This study
-74.0732	-42.5555	This study
-74.0688	-42.5491	This study
-74.066	-42.1527	This study
-74.0619	-42.1514	This study
-74.0633	-42.1513	This study
-74.0589	-42.1502	This study
-74.0585	-42.1499	This study
-74.0585	-42.1497	This study
-74.0567	-42.1484	This study
-74.053	-42.1467	This study
-74.0357	-42.1162	This study
-74.0351	-42.1152	This study
-74.0334	-42.1137	This study
-74.0339	-42.1126	This study
-74.0346	-42.112	This study
-74.0397	-42.1114	This study
-74.0355	-42.1108	This study
-74.0453	-42.1079	This study
-74.0462	-42.1075	This study
-74.0465	-42.1071	This study
-74.0469	-42.1067	This study
-74.0508	-42.1066	This study
-74.0474	-42.1065	This study
-74.0477	-42.1063	This study
-74.0509	-42.1063	This study
-74.0544	-42.106	This study
-74.0521	-42.1059	This study

-74.0519	-42.1049	This study
-74.0408	-42.1023	This study
-74.0399	-42.0998	This study
-74.0347	-42.0833	This study
-74.0346	-42.0792	This study
-74.0311	-42.0762	This study
-73.7706	-42.0622	This study
-73.7706	-42.0622	This study
-73.7706	-42.0622	This study
-74.000	-42.000	This study
-73.8283	-41.9583	This study
-73.8283	-41.9583	This study
-73.8283	-41.9583	This study
-73.8283	-41.9583	This study
-73.8283	-41.9583	This study
-73.8283	-41.9583	This study
-72.9695	-37.8262	This study
-73.0342	-37.825	This study

References

D'Elía, G., Ortloff, A., Sanchez, P., Guiñez, B., Varas, 2013. A new geographic record of the endangered Darwin's fox *Lycalopex fulvipes* (Carnivora: Canidae): Filling the distributional gap. Rev. Chil. Hist. Nat. 86, 485–488. doi:10.4067/S0716-078X2013000400010

Dieguez, A., 2014. Niños ayudaron a encontrar al esquivo zorro de Darwin. Las Ultim. Not. 14.

Farias, A.A., Sepúlveda, M.A., Silva-rodríguez, E.A., Eguren, A., González, D., Jordán, N.I., Ovando, E., Stowhas, P., Svensson, G.L., 2014. A new population of Darwin's fox (*Lycalopex fulvipes*) in the Valdivian Coastal Range. Rev. Chil. Hist. Nat. doi:10.1186/0717-6317-1-3

González-Acuña, D., Briceño, C., Cicchino, A., Funk, S.M., Jiménez, J., 2006. First records of *Trichodectes canis* (Insecta: Phthiraptera: Trichodectidae) from Darwin's fox, *Pseudalopex fulvipes* (Mammalia: Carnivora: Canidae). Eur. J. Wildl. Res. 53, 76–79. doi:10.1007/s10344-006-0066-y

Jiménez, J., Briceñoc, C., Alcaínoe, H., Funk, S., 2012. Coprologic survey of endoparasites from Darwin's fox (*Pseudalopex fulvipes*) in Chiloé, Chile. Arch Med Vet 97, 93–97.

Jimenez, J.E., Marquet, P.A., Medel, R.G., Jaksic, F.M., 1991. Comparative ecology of Darwin's Fox (*Pseudolapex fulvipes*) in mainland and island settings of southern Chile. Rev. Chil. 63, 177–186.

Jiménez, J.E., Mcmahon, E., 2004. Darwin's fox *Pseudalopex fulvipes* (Martin, 1837)

Critically Endangered – CR: C2a(ii) (2004), in: Hoffmann, M., Mech, D., Sillero-Zubiri, C. (Eds.), Canids: Foxes, Wolves, Jackals and Dogs: Status Survey and Conservation Action Plan. IUCN Canid Specialist Group, Cambridge, pp. 50–55.

Killian, K., Kristina Killian, 2005. Telemetrische Untersuchung zum Einfluss des Menschen auf die Lebensraumnutzung des Darwin-Fuchses (*Pseudalopex fulvipes*, Martin 1837) auf Chiloé, Chile. Universität Hamburg.

Vasquez-Grandon, A., 2011. Conservación del zorro de Darwin en la Cordillera de Nahuelbuta., in: Preservación y Conservación de la Flora y Fauna de la Cordillera de Nahuelbuta. Angol, p. 32.

Vilà, C., Leonard, J. A., Iriarte, A., O'Brien, S.J., Johnson, W.E., Wayne, R.K., 2004. Detecting the vanishing populations of the highly endangered Darwin's fox, *Pseudalopex fulvipes*. Anim. Conserv. 7, 147–153. doi:10.1017/S1367943004001271
