# Supplementary material for: Ecological niche modeling re‐examined: A case study with the Darwin's fox
Source: Ecol Evol. 2018 Apr 16;8(10):4757–70. doi: 10.1002/ece3.4014 (PMC5980497; doi:10.1002/ece3.4014)
Supplement: Supplementary file 2 [file ECE3-8-4757-s002.pdf]

**Ecological Niche Modeling Re-examined: A Case Study with the Darwin's Fox**

Luis E. Escobar[1], Huijie Qiao[2*], Javier Cabello[3,4], A. Townsend Peterson[5]

[1]Department of Fish and Wildlife Conservation, Virginia Tech, Blacksburg, Virginia, 24061 USA.
[2]Key Laboratory of Animal Ecology and Conservation Biology, Institute of Zoology, Chinese Academy of Sciences, Beijing, 100101 China.
[3]Centro de Conservación de la Biodiversidad Chiloé-Silvestre, Chiloé Island, Los Lagos, Chile.
[4]Facultad de Medicina Veterinaria, Universidad San Sebastián, Campus Pichi Pelluco, Lago Panguipulli, 1390 Puerto Montt, Chile.
[5]Biodiversity Institute, University of Kansas, Lawrence, Kansas, 66045 USA.

* Corresponding author: Huijie Qiao, qiaohj@ioz.ac.cn

**Supplementary material S2. Complementary methods.**

**A. Step by step guide to partial ROC evaluations.**

Ecological niche model evaluation using Partial ROC software (Barve 2008).

For details and theoretical bases of this model evaluation method, see Peterson *et al.* (2008). Briefly, models are calibrated with a set of data and are evaluated with an independent set of data. Evaluations consider the omission rate and the proportion of the evaluation area predicted as suitable, based on a sequence of threshold values across an evaluation area selected *a priori*. This spectrum of model predictions in a two-dimensional error space is then refined based on a user-defined error tolerance (*E*), which corresponds to how much "noise" is likely present in the occurrence dataset. For example, *E* = 5% would allow up to 5% of occurrences to be left out of thresholded predictions, as they may represent erroneous georeferencing rather than model failure (Peterson *et al.* 2008). For each threshold, omission rates are calculated, and only the part of the error space for which the omission rate is $<E$ is considered. The curve tracing the omission values and proportions of the study area is then divided by the corresponding null expectations (see Fielding and Bell 1997) to create an "area under the curve (AUC) ratio. This process is done several times in Partial ROC from a series of replicates using a percentage of evaluation points (usually 50%) selected randomly. This bootstrap step allows testing to establish whether a given AUC ratio is significantly elevated about 1, which is the performance of a random classifier (Peterson *et al.* 2008).

**Steps to run the evaluations using Partial ROC software.**

1. The data available for the species should be split in a set for calibration and a set for evaluation.

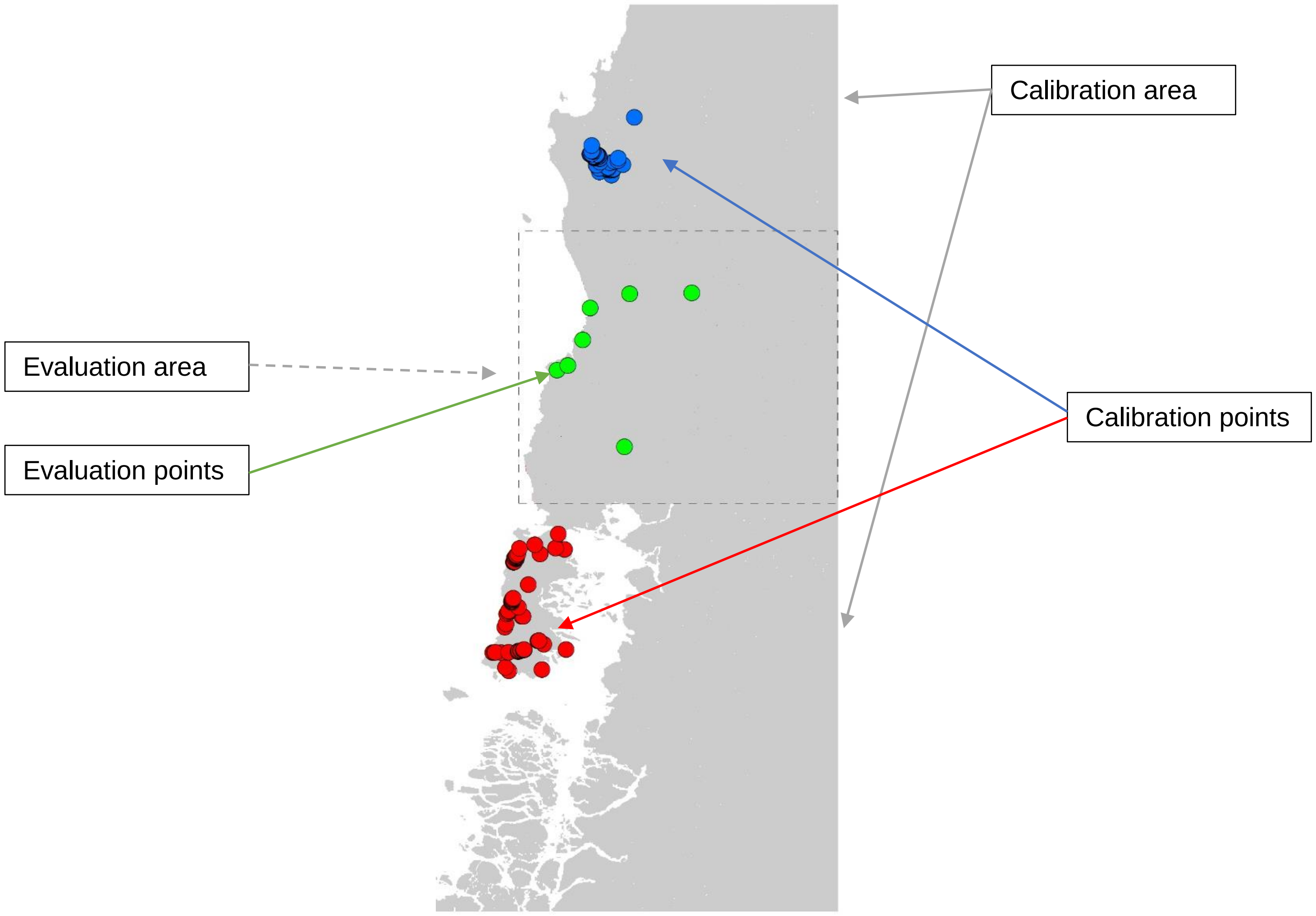

2. Models must be calibrated with one set of data: calibration points.

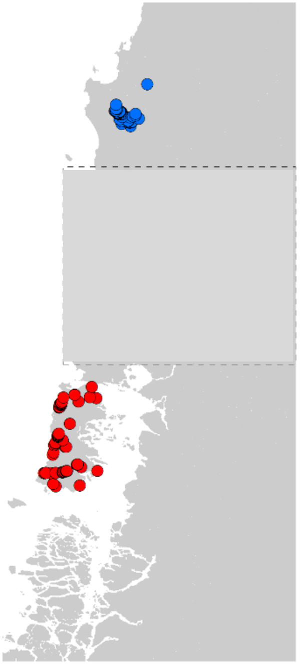

3. Once the model is calibrated using the calibration occurrences, cut the evaluation area that was defined *a priori*, and that is independent of the area using for model calibration (Hurlbert 1984). The evaluation points are distributed across this area, and the partial ROC test evaluates the coincidence between the points and the model predictions. Below is an example of the models' output in continuous format cut to the evaluation region. Pixels with high values are in red while pixels with low values are in green. Note that the coincidence of the evaluation points (black crosses) with the model predictions is the key to success of these evaluations … in particular, the GLM prediction shows good correspondence.

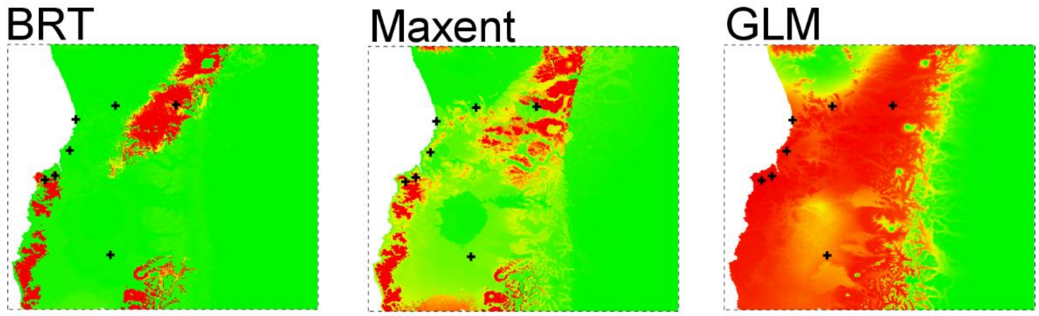

4. The raster file of the evaluation area should be converted to ascii. However, Partial ROC cannot read decimals. Thus, multiply the raster by 10000 and round the pixel values to obtain only integers. For example, rasters from Maxent may originally range from 0 to 0.9, after this step, values of the ascii should range from 0 to 9,000.

5. Count the number of pixels in the raster for each value of prediction. Following the example, count how many pixels have a value of "0" and how many pixels have a value of "9,000."

6. Make a list of the values of prediction from the raster ordered by the values of prediction (left column) sorted from smallest to largest. Note that suitability values of "0" should be counted (if no pixels are predict as "0"suitability add zero as the count of pixels for this value). Save this list as a comma delimited file, two columns, with header:

| value of suitability | count of pixels |
|---:|---:|
| 0 | 96179 |
| 1000 | 2569 |
| 2000 | 2488 |
| 3000 | 3670 |
| 4000 | 1443 |
| 5000 | 1816 |
| 6000 | 2108 |
| 7000 | 2579 |
| 8000 | 669 |
| 9000 | 3729 |
| 10000 | 16967 |

7. Now assign a value of prediction to each evaluation point. Use the evaluation points to “extract” the values of prediction from the continuous raster. Then make a list of the values of suitability for these points and add an identification number to each point. Rank the values of suitability for each point (right column) sorted from largest to smallest. Save this list as a comma delimited file, two columns, with header:

| ID | Suitability value |
|---|---|
| 1 | 10000 |
| 2 | 10000 |
| 3 | 3000 |
| 4 | 0 |
| 5 | 0 |
| 6 | 0 |
| 7 | 0 |

8. Now we have the two comma delimited files, one containing the values of prediction in the area of evaluation, and another file containing the values of prediction in the evaluation points. These files are used in Partial ROC.

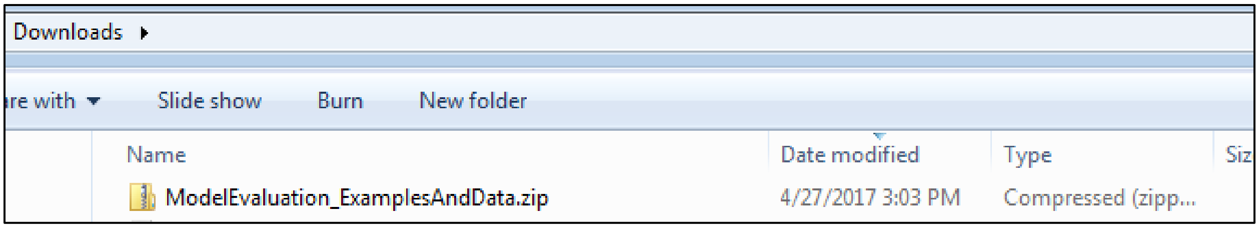


9. First download the software from here: https://kuscholarworks.ku.edu/bitstream/handle/1808/10059/ModelEvaluation_ExamplesAndData.zip?sequence=2&isAllowed=y which is the original source of the software from the paper explaining its use and interpretation see (Peterson 2012).

10. Decompress the file:

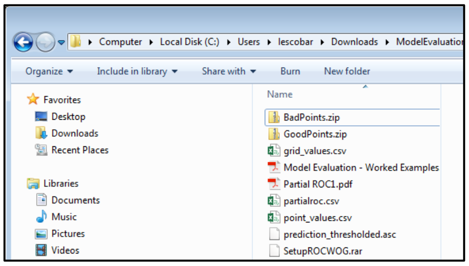


11. You will see one file called “SetupROCWOG.rar” decompress.

12. Now you will have a file called “SetupROCWOG.xyz” rename this file changing the extension from “.xyz” to “.exe”

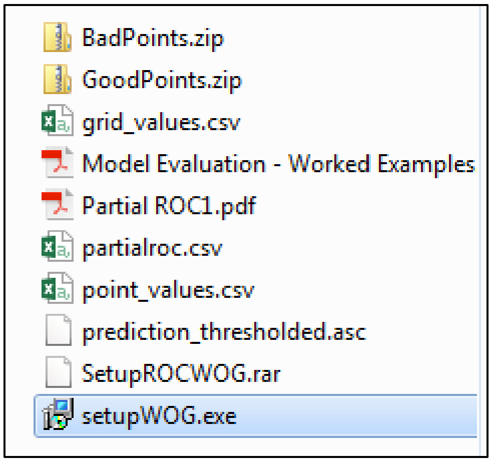

13. Double click in the .exe file and install the software as you will do with any other Windows based software. Go to Start, (or to the short cut in the Desktop) to open the Partial ROC software:

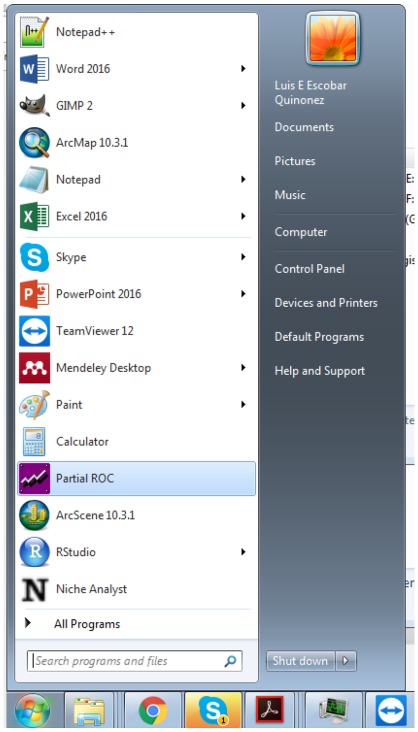


14. You may have some problems to open the software, with a message like this:

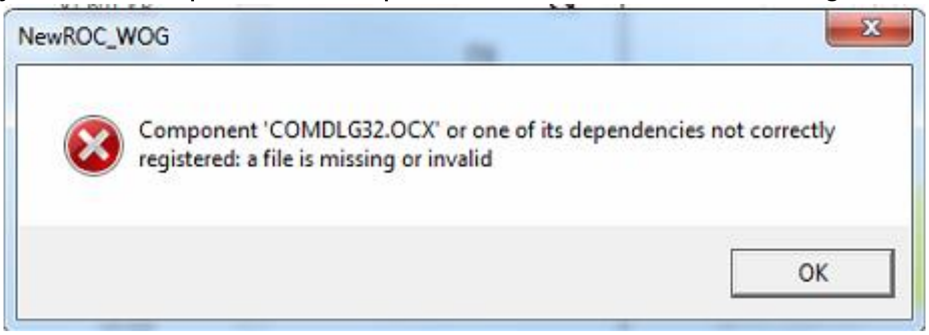


15. No worries, just install the component “COMDLG32.OCX”

16. Go to this link: http://www.bioinformatics.org/snp-tools-excel/install_comdlg32.htm or simple download the file Comdlg32.ocx file.

17. Then copy and paste the file in **C:\Windows\SysWOW64**

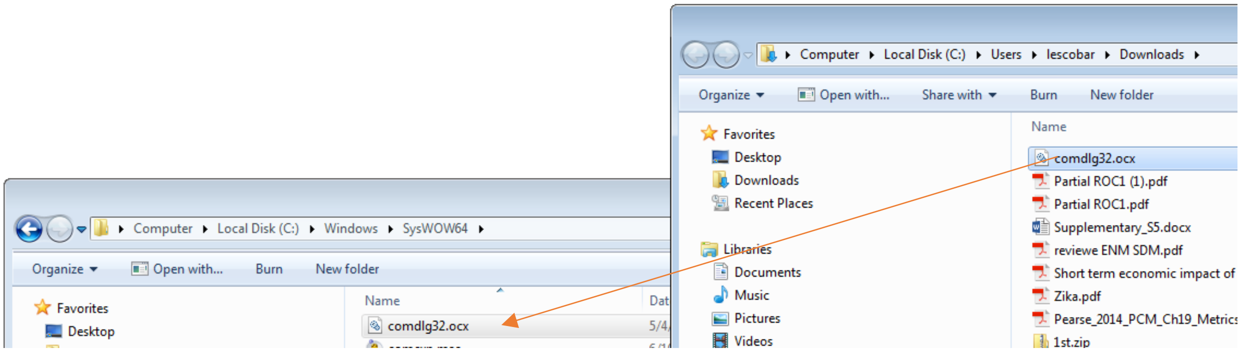


18. Go to Start, search for the "Command Prompt" icon, right click, Run as administrator:

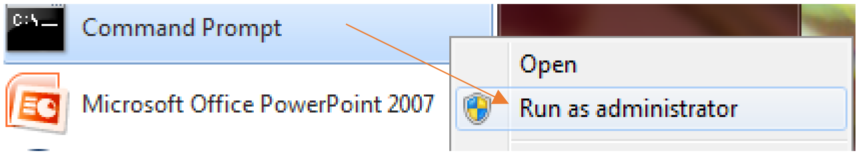


19. In the cmd (a black window) type:
**REGSVR32 C:\Windows\System32\comdlg32.ocx** Then push enter:

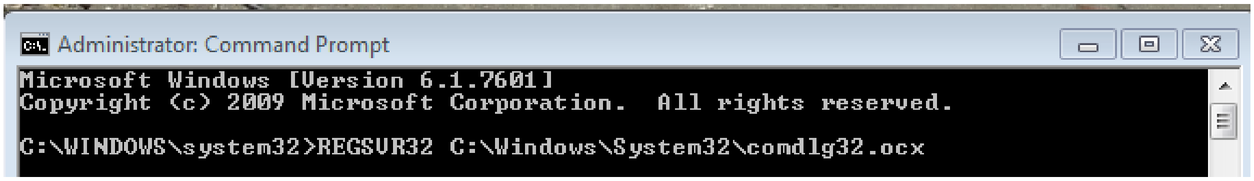


20. If the installation was successful after you will see this message:

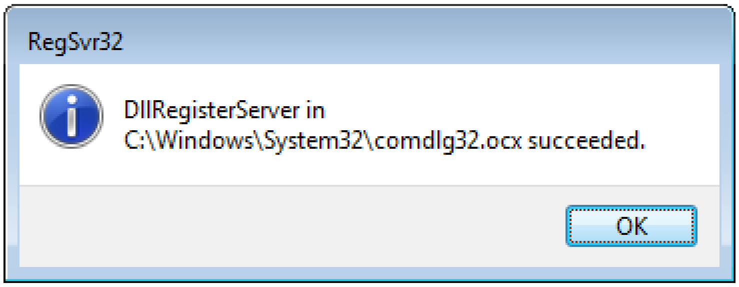

21. Open Partial ROC:

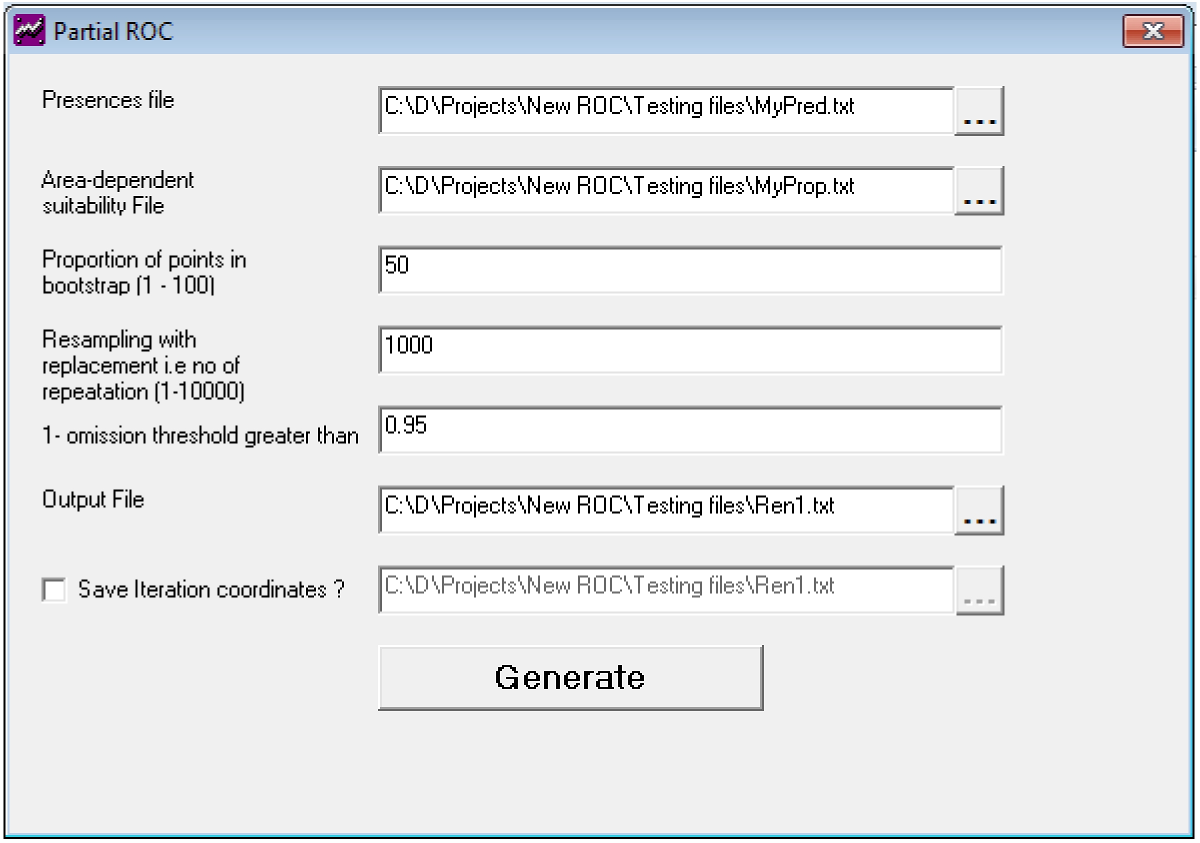

22. Replace the file paths from the examples in the software to your real files of evaluation areas and evaluation points, also define a folder and file name to save the results.

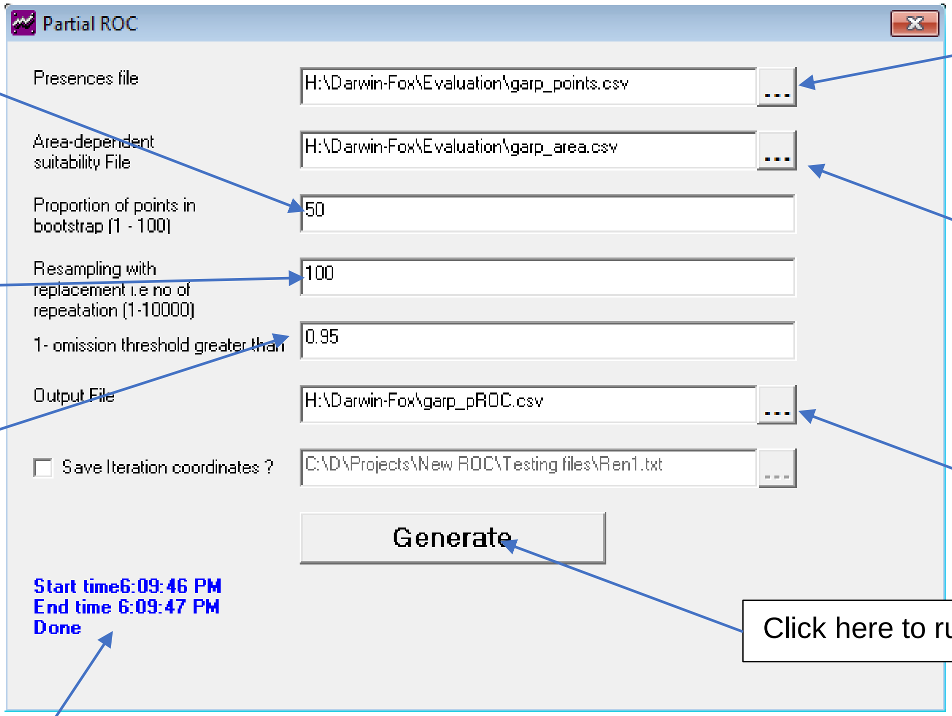


Percentage of evaluation points that will be used to test each evaluation replicate

Number of bootstrap replicates (select at least 100 or 1000)

For an error of 5% set 0.95. This % of error will be used as threshold to construct binary predictions.

Local time when the analysis was started and completed.

Select the .csv file with suitability values of evaluation points

Select the .csv file with suitability values by pixel in the evaluation area

Select the folder to save the results.

Click here to run!

23. If the analysis was completed successfully this window will appear:

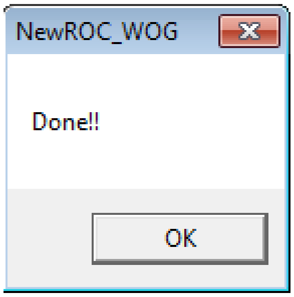


24. The output file will contain a csv with the Partial ROC ratios. Values above 1 represent predictions—of evaluation points in the evaluation area—better than by random. You will expect at least 95% of ratios above 1 for an alpha <0.05.

| IterationNo | AUCatValue 0.95 | AUCAt 0.5 | Ratio |
|---|---|---|---|
| 0 | 6.11E-02 | 6.07E-02 | 1.006557 |
| 1 | 0.104801767 | 0.10171205 | 1.030377 |
| 2 | 5.24E-02 | 5.23E-02 | 1.001924 |
| 3 | 0.104801767 | 0.10171205 | 1.030377 |
| 4 | 0.104801767 | 0.10171205 | 1.030377 |
| 5 | 3.49E-02 | 3.52E-02 | 0.992786 |
| 6 | 5.24E-02 | 5.23E-02 | 1.001924 |
| 7 | 5.24E-02 | 5.23E-02 | 1.001924 |
| 8 | 0.104801767 | 0.10171205 | 1.030377 |
| 9 | 0.104801767 | 0.10171205 | 1.030377 |
| 10 | 5.24E-02 | 5.23E-02 | 1.001924 |
| 11 | 0.104801767 | 0.10171205 | 1.030377 |
| 12 | 3.49E-02 | 3.52E-02 | 0.992786 |
| 13 | 5.24E-02 | 5.23E-02 | 1.001924 |

***Partial ROC metric is also available in NicheA 3.0 (Qiao et al. 2015, 2016).***

**References**


Barve, N. (2008) Tool for Partial-ROC. Available at: http://kuscholarworks.ku.edu/dspace/handle/1808/10059.

Fielding, A.H. & Bell, J.F. (1997) A review of methods for the assessment of prediction errors in conservation presence/absence models. *Environmental Conservation*, **24,** 38-49.

Hurlbert, S.H.. (1984) Pseudoreplication and the design of ecological field experiments. *Ecological Monographs* **54**, 187–211.

Peterson, A.T.T. (2012) Niche modeling: Model evaluation. *Biodiversity Informatics* **8**, 41.

Peterson, A.T.T., Papes, M., Soberón, J., Papeş, M. and Soberón, J. (2008) Rethinking receiver operating characteristic analysis applications in ecological niche modeling. *Ecological Modelling* **213**, 63–72.

Qiao, H., Peterson, A.T., Campbell, L.P., Soberón, J., Ji, L. and Escobar, L.E. (2016) NicheA: Creating virtual species and ecological niches in multivariate environmental scenarios. *Ecography* **39**, 805–813.

Qiao, H., Soberón, J., Escobar, L.E., Campbell, L. and Peterson, A.T. (2015) NicheA. Version 3.0.1. Available at: http://nichea.sourceforge.net/ [Accessed December 1, 2015].

**Supplementary material S2. Complementary methods.**

**B. Scripts employed in this study.**

*(Note: This is a brief guide to access the scripts used in this study. For more details of the R scripts for models developed using dismo, biomod2, and hypervolume (KDE) packages visit https://github.com/qiaohj/DarwinFox; for details of the NicheA scripts visit https://github.com/qiaohj/NicheA.)*

Tip: all the JAVA scripts are shared via an SVN service, which address is svn://mmweb.animal.net.cn/nichea/trunk/NicheA (Note: The SVN needs to be installed to manage the source code, for more information please visit https://tortoisesvn.net/)

1. Convert the raster files in folder 'DarwinFox\environments\ASC' from ASCII to GeoTIFF format via function 'Raster conversion' in NicheA.
   Webpage: http://nichea.sourceforge.net/function_conversion_tool.html
2. Launch principal component analysis in NicheA with the converted raster files above, and saving the results to the folder 'DarwinFox\environments\PCA'.
   Webpage: http://nichea.sourceforge.net/function_pca.html
3. Create Virtual N for **D**$_c$, **D**$_s$, **D**$_n$ in NicheA, and saving the results to the folder 'DarwinFox\V_N'.
   Webpage: http://nichea.sourceforge.net/function_create_g4.html
4. Run Maxent model
   a) Export virtual Ns to Maxent format, and saving them to the folder 'DarwinFox\Maxent'.
      Webpage: http://nichea.sourceforge.net/function_export_enm.html
   b) Run Maxent model with default parameters.
5. Run GBM/BRT model,
   a) Export virtual Ns to dismo format.
   b) Run the R script, 'DarwinFox\dismo\C_N\rscript.1.r'
   c) Run dismo_text2raster in Java, convert the output of dismo model from TEXT to GeoTIFF format.

6. Run MVE model
    a) Export the model via ‘Export N as continue raster’ function in NicheA.
7. Run GLM model
    a) Export virtual Ns to BIOMOD2 format.
    b) Run the R script, ‘DarwinFox\BIOMOD2\C_N\glm.r’
    c) Convert the results from IMG format to GeoTIFF in NicheA
8. Running GARP model
    a) Export virtual Ns to openModeller format.
    b) Create request.txt file manually
    c) Run om_console request.txt to generate the GARP model
9. Run KDE model
    a) Export virtual Ns to MKDE format.
    b) Run the R script, “DarwinFox\KDE\C_N\ rscript.1.r”
    c) Run mkde_text2raster in Java to convert the output of KDE model from TEXT to GeoTIFF format.
10. Run format_enm_result in Java to convert result to [0, 1]
11. Calculate MTP threshold values in NicheA for every C_N model.
    Webpage: http://nichea.sourceforge.net/function_threshold.html
12. Create the virtual N inside and outside of MVE of all in NicheA.
    Webpage: http://nichea.sourceforge.net/function_create_g.html
13. Run getENMValue in Java to get all the results of occurrences.
14. Calculate extrapolation based assessment, run calculateDistancetoCentroid and calculateEDistancetoCentroid functions in Java, to get the result (E_result.csv, MVE_result.csv)
15. For Jaccard Index, run “Quantifying niche similarity” functions in NicheA.
    Webpage: http://nichea.sourceforge.net/function_niche_overlap.html
16. For AICc, run “Calculate AIC/BIC” function in NicheA.
    Webpage: http://nichea.sourceforge.net/function_aic.html
