# Supplementary material for: Ecological niche modeling re‐examined: A case study with the Darwin's fox
Source: Ecol Evol. 2018 Apr 16;8(10):4757–70. doi: 10.1002/ece3.4014 (PMC5980497; doi:10.1002/ece3.4014)
Supplement: Supplementary file 3 [file ECE3-8-4757-s003.pdf]

Ecological Niche Modeling Re-examined: A Case Study with the Darwin's Fox

Luis E. Escobar[1], Huijie Qiao[2*], Javier Cabello[3,4], A. Townsend Peterson[5]

[1]Department of Fish and Wildlife Conservation, Virginia Tech, Blacksburg, Virginia, 24061 USA.
[2]Key Laboratory of Animal Ecology and Conservation Biology, Institute of Zoology, Chinese Academy of Sciences, Beijing, 100101 China.
[3]Centro de Conservación de la Biodiversidad Chiloé-Silvestre, Chiloé Island, Los Lagos, Chile.
[4]Facultad de Medicina Veterinaria, Universidad San Sebastián, Campus Pichi Pelluco, Lago Panguipulli, 1390 Puerto Montt, Chile.
[5]Biodiversity Institute, University of Kansas, Lawrence, Kansas, 66045 USA.

* Corresponding author: Huijie Qiao, qiaohj@ioz.ac.cn

Supplementary material S3. Complementary results.

A. Frequency of Darwin's Fox (*Lycalopex fulvipes*) occurrences according to annual mean temperature.

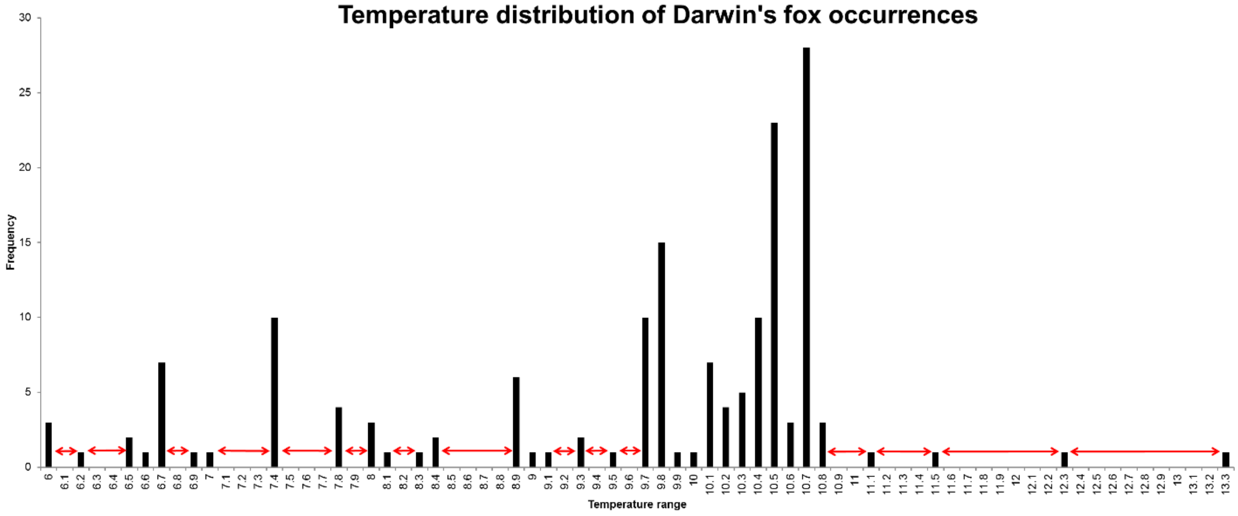

B. **Table of threshold values based on 0% and 5% omission error in calibration occurrences.**

Algorithm	0%	5%	% change	Histogram of predicted values
GLM	0.002	0.4372	43.52%	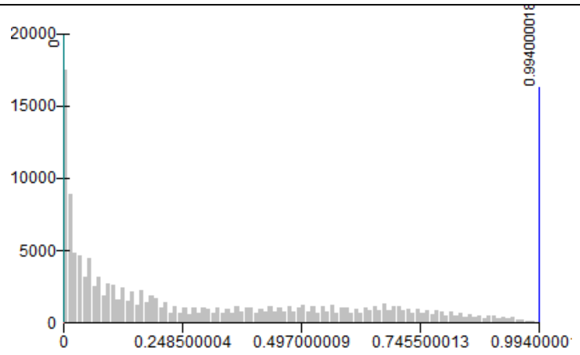
BRT	0.082574	0.466733	38.42%	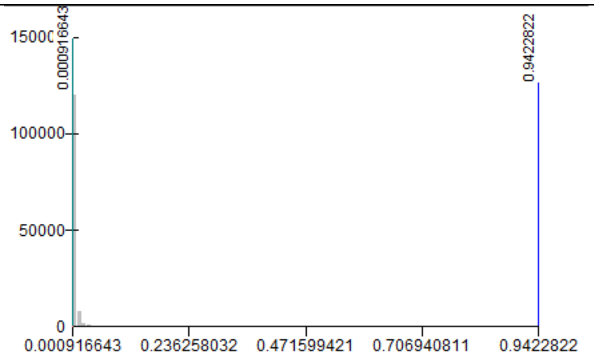
GARP	0.1	0.465	36.5%	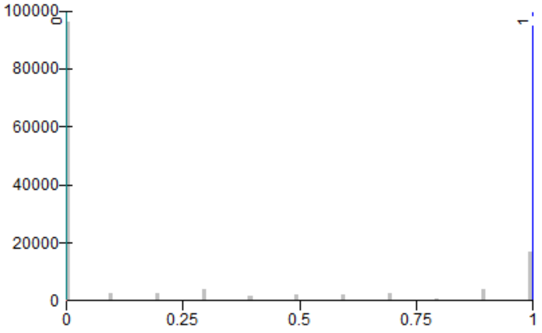

Maxent	0.000027	0.167793	17%	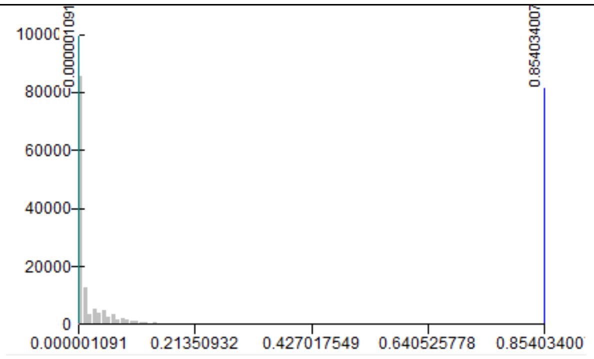
NicheA	0.000095	0.000762	0.06%	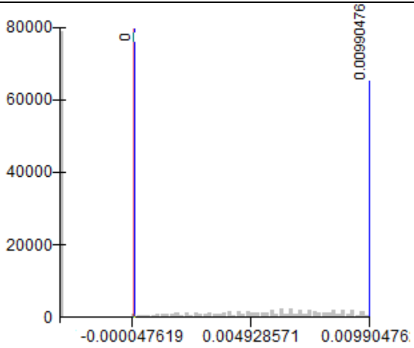
KDE	1	1	0%	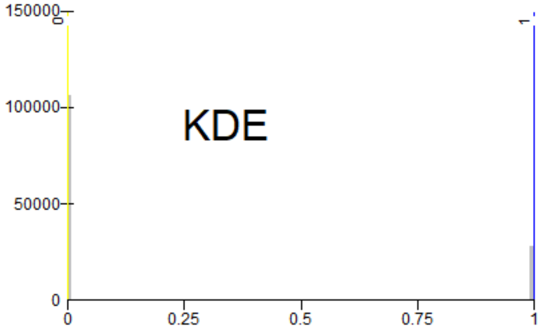

C. Continuous and binary models of Darwin's Fox in the evaluation area (Threshold = 5%).

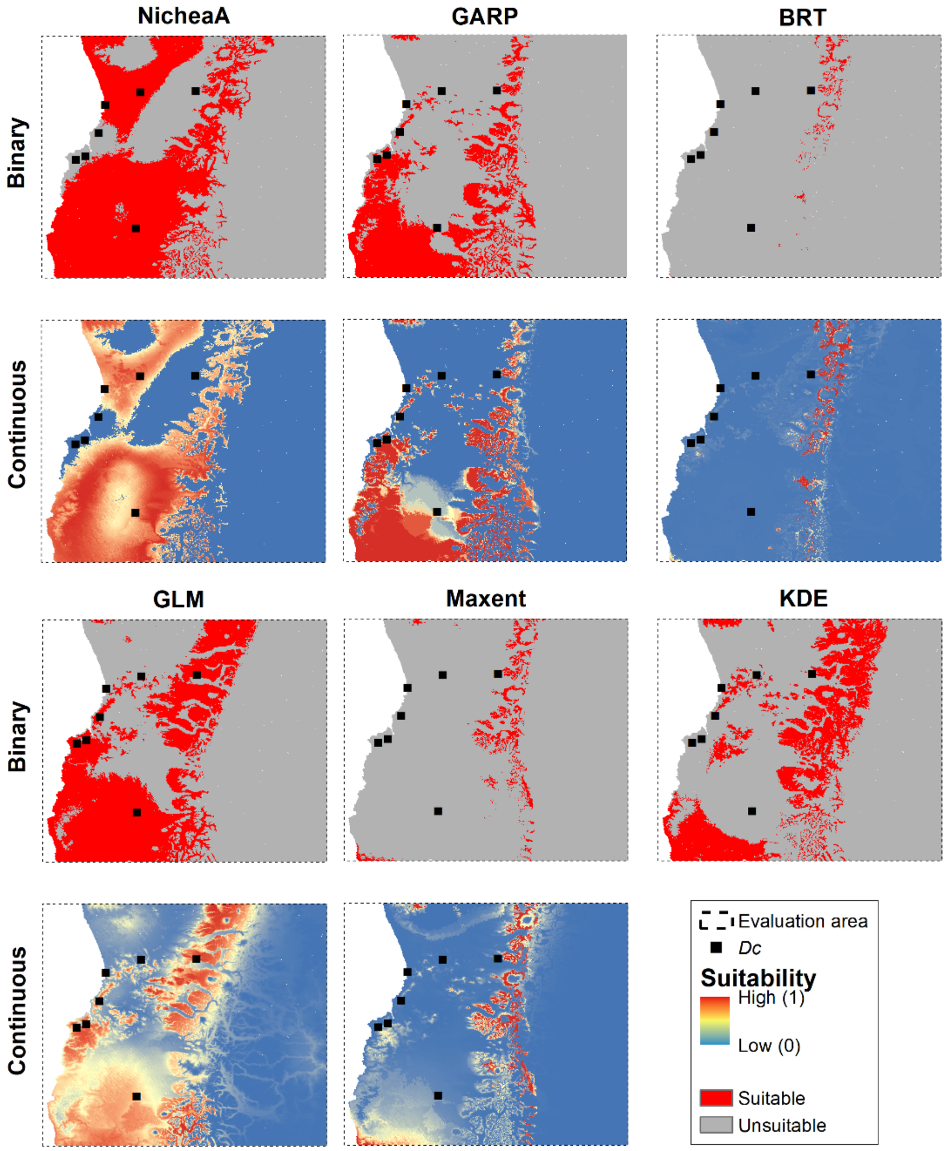

Models calibrated using northern and southern (**D**$_s$ + **D**$_n$) Darwin's Fox occurrences, projected in the evaluation area (dashed line). Independent occurrences from the central population (**D**$_c$; black squares) are used to evaluate the model in terms of predictions in continuous (i.e., range of colors; highly suitable = red, unsuitable = blue) and binary outputs (i.e., suitable = red, unsuitable = gray). Binary models were generated based on 5% omission error from calibration occurrences. NicheA: minimum-volume ellipsoid; GARP: genetic algorithm for rule-set production; BRT: boosted regression trees; GLM: generalized linear model; Maxent: maximum entropy; KDE: hypervolume multivariable kernel density estimation.

D. Model evaluations based on interpolation and extrapolation in environmental space (Threshold = 5%).

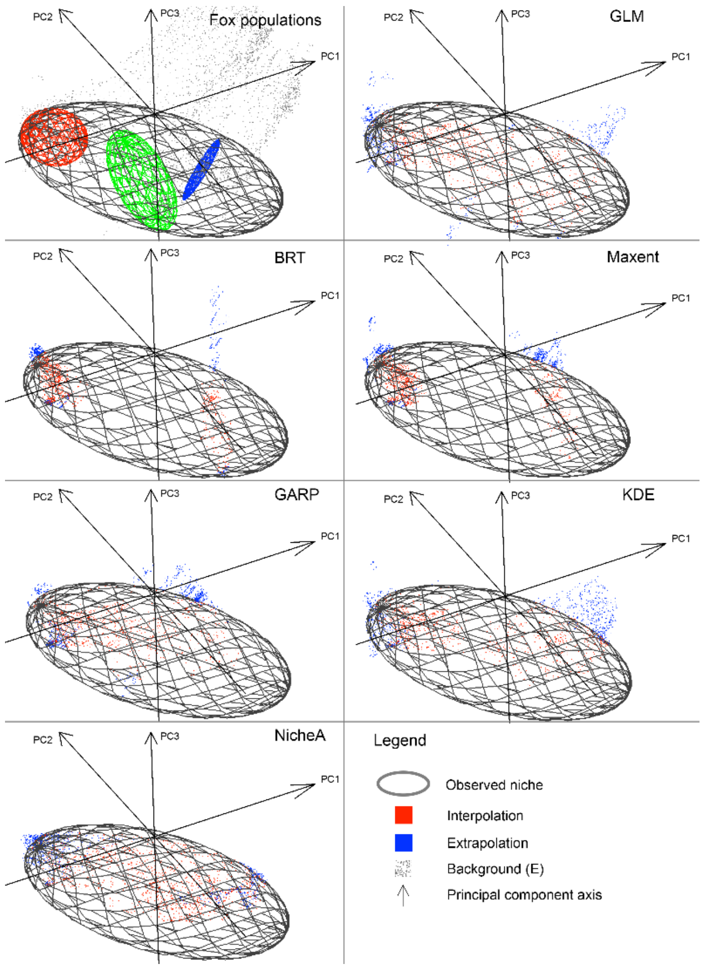

Top left: Darwin's Fox populations, from the northern (blue ellipsoid), central (green ellipsoid), and southern (red ellipsoid), populations were enclosed to generate observed ecological niche hypotheses; the environmental background is shown in this panel as gray points. Subsequent panels: predictions were categorized according to environmental interpolation (red points) as predictions inside the ellipsoid and environmental extrapolation (blue points; see Methods) as predictions outside the ellipsoid; the environmental background is not shown in these panels for better visualization of models output. GLM: generalized linear model; BRT: boosted regression trees; Maxent: maximum entropy; GARP: genetic algorithm for rule-set production; KDE: hypervolume multivariable kernel density estimation; NicheA: minimum-volume ellipsoid. Note that predictions by some models resemble the background cloud (e.g., GLM and Maxent), suggesting that all the conditions available in the model calibration area were predicted suitable by the model via model interpolation (red points) or extrapolation (blue points).

E. **Darwin's Fox (*Lycalopex fulvipes*) potential distribution map selected and final model assemble.**

NicheA model based on all the occurrences and a threshold assuming 0% omission error in calibration occurrences. This model was selected because, during the rigorous model evaluation process, this modeling approach had the highest scores for our model selection criteria: low extrapolation and high interpolation. Red denotes areas are suitable and gray areas are not suitable for the species. Black crosses are the occurrences available for this study.

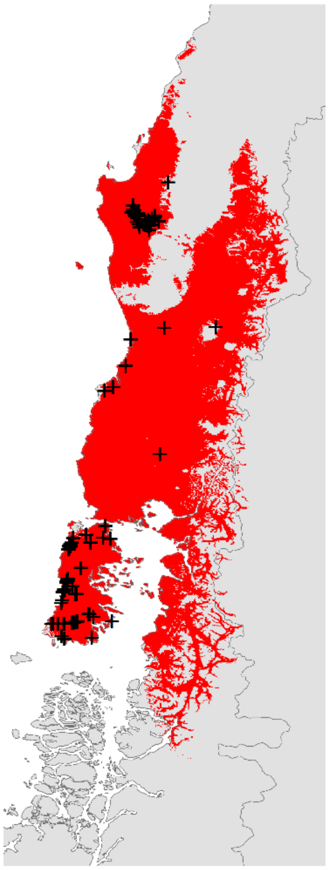

Final models for each algorithm (i.e., generalized linear model; boosted regression trees; maximum entropy; genetic algorithm for rule-set production; hypervolume multivariable kernel density estimation; minimum-volume ellipsoid), calibrated using all occurrences. Model agreement: sum of the maps showing areas of high (red) and low (green) agreement of models (i.e., all models predicted suitable a specific pixel). Binary models based on 0% omission error in calibration occurrences.

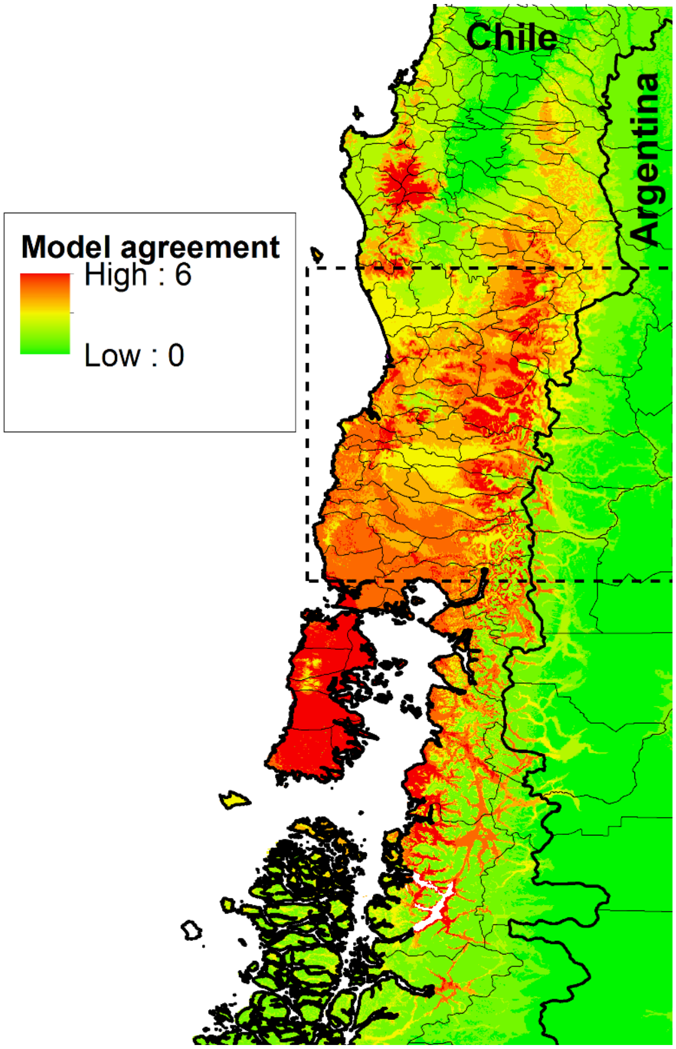

Interpretation of maps

We remain intrigued by the limited and restricted nature of the distribution of Darwin's Fox, although the model of the species' potential distribution show less disjunct and fragmented range than had been thought (Fig. S4.1.). We suspect that biological interactions at local scales (e.g., competition with dogs, habitat loss, human-wildlife conflicts; Baillie, Hilton-Taylor & Stuart 2004) may be limiting the range and abundance of the species in areas that are otherwise environmentally suitable for the species according to our final model. This suite of factors might be responsible for the endangered status of this species. In the central regions of the species' range predicted by our model (Fig. S4.1), there is today high fragmentation of native forest with high density of roads and broad areas of crop lands, which may be related to low abundance of the species in these areas. What's more, northern and southern populations show minimal or no genetic exchange as a result of geographic isolation, likely reflecting combined effects of anthropogenic land use change and natural barriers. For example, the genetic pool of the Darwin's Fox population on Chiloé Island has been isolated geographically from population in continental Chile from ~30,000 years, while the population in the northern area of the range, in Nahuelbuta Reserve, has been isolated recently due to habitat loss reducing genetic flow (Cabello, J. *pers. comm.*).
