# Supplementary material for: Ecological niche modeling re‐examined: A case study with the Darwin's fox
Source: Ecol Evol. 2018 Apr 16;8(10):4757–70. doi: 10.1002/ece3.4014 (PMC5980497; doi:10.1002/ece3.4014)
Supplement: Supplementary file 4 [file ECE3-8-4757-s004.docx]

**Supplementary Material S4. Complementary Maxent parameterization based on 20 different regularization values and 31 feature combinations for a total 610 Maxent models.**

Using ENMeval, we ran 1220 Maxent models with different parameter combinations, which were 20 regularization multipliers (RM; from 0.5 to 10 by 0.5) times 31 features ("L", "Q", "H", "P", "T", "LQ", "LH", "LP", "LT", "QH", "QP", "QT", "HP", "HT", "PT", "LQH", "LQP", "LQT", "LHP", "LHT", "LPT", "QHP", "QHT", "QPT", "HPT", "LQHP", "LQHT", "LQPT", "LHPT", "QHPT", "LQHPT"). This allowed to compare between default Maxent and a best Maxent model (i.e., lowest AICc; for details please see (Muscarella et al. 2014)).

**Figure S4.1.** Histogram showing AICc values with calibration data only (**D***_s_* + **D***_n_* Darwin’s Fox occurrences; see Methods in the manuscript). AICc values ranged between 5976.576 (Features: LHT, RM=1) and 2119.946 (Feature: LQPT, RM=2).


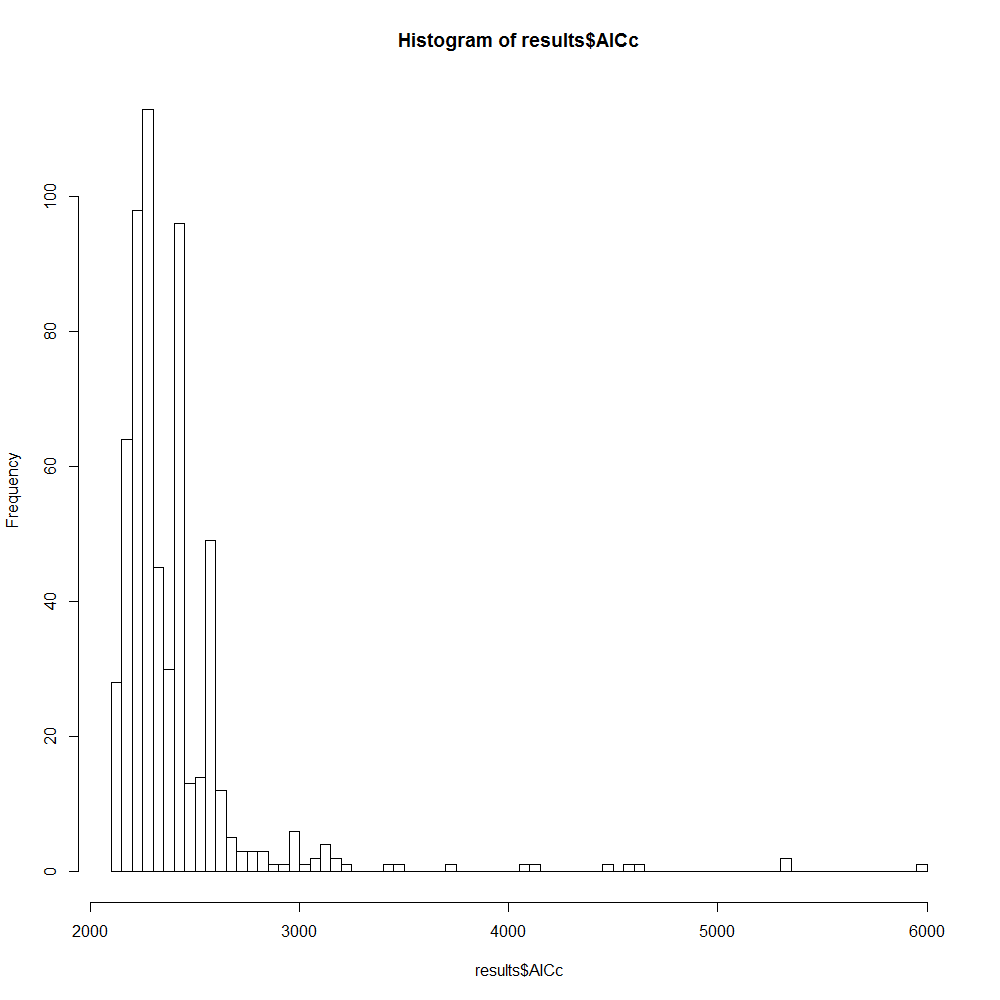


**Figure S4.2.** Histgram showing AICc values with all available occurrences to generate final models (D*_n_* + D*_c_* + D*_s_*). AICc values ranged between 26106.46 (Features: H, RM=2.5) and 2315.147 (Features: LPT, RM=2).


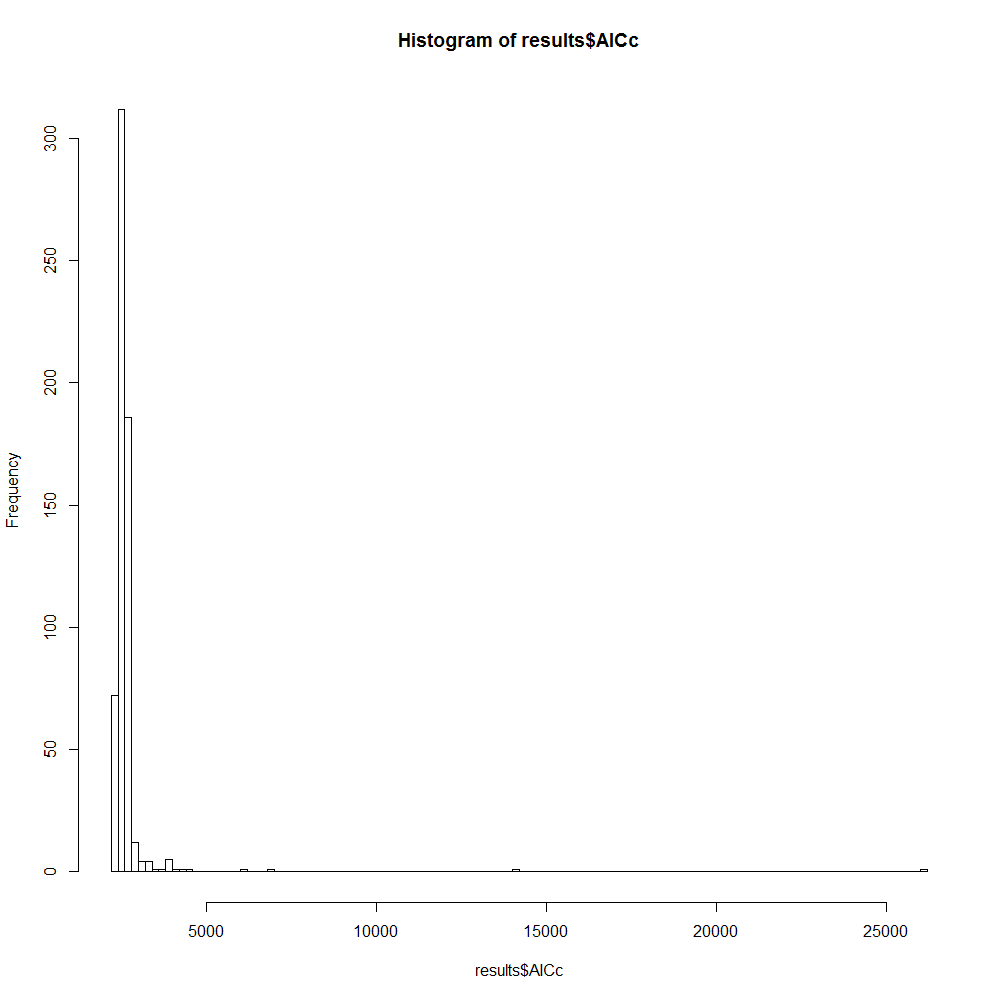


**Figure S4.3.** Potential geographic distribution of Darwin’s Fox based on best partial (D*_n_* + D*_s_*) and final model (D*_n_* + D*_c_* + D*_s_*).

| D*_n_* + D*_s_* occurrences, Features: LQPT, RM=2 | D*_n_* + D*_c_* + D*_s_* occurrences, Features: LPT, RM=2 |
| --- | --- |
| 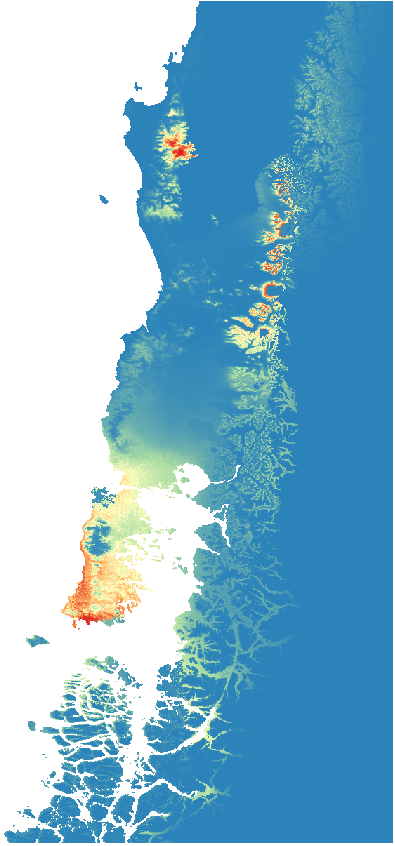 | 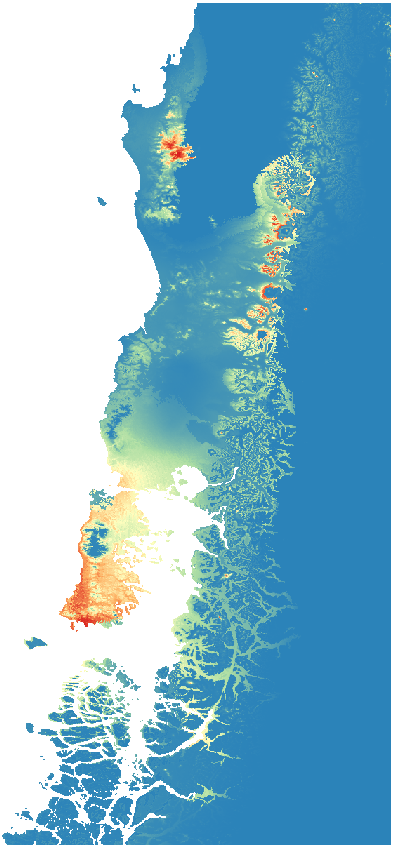 |

Threshold values required to generate binary maps:

| **Omission error** | **(D*_n_* + D*_c_* + D*_s_*)** | **(D*_n_* + D*_s_*)** |
| --- | --- | --- |
| 0% | 0.000229 | 0.000004 |
| 5% | 0.057442 | 0.132013 |

**Table S4.1.** Summary of Akaike information criterion (AIC) values for default model and best model (i.e., lowest AIC; for details please see (Muscarella et al. 2014)) calibrated using northern and southern populations (i.e., D*_n_* + D*_s_*) and all occurrences available (D*_n_* + D*_c_* + D*_s_*).

|  | K | Ln(likelihood) | AIC |
| --- | --- | --- | --- |
| *Calibration models* D*_n_* + D*_s_* | | | |
| Maxent (default) | 57 | -1061.09 | 2236.17 |
| Maxent (optimized) | 23 | -1092.85 | 2231.70 |
| *Final models* D*_n_* + D*_c_* + D*_s_* | | | |
| Maxent (default) | 55 | -1173.17 | 2456.33 |
| Maxent (optimized) | 19 | -1201.98 | 2441.96 |

**Table S4.2.** Summary of true omission error evaluations based on validation data from the novel population D*_c_* (n = 7) for “default” and “best” Maxent model. Binary maps based on an *a priori* percentage of omission error tolerance of 0% and 5% in the calibration data D*_n_* and D*_s_*.

| Model calibrated using D*_n_* + D*_s_* | Omission Rate | | Area predicted suitable | | CBP (*P* value) | |
| --- | --- | --- | --- | --- | --- | --- |
|  | 0% | 5% | 0% | 5% | 0% | 5% |
| Maxent (default) | 0.00 | 1.00 | 0.96 | 0.03 | <0.001 | >0.05 |
| Maxent (optimized) | 0.00 | 1.00 | 0.89 | 0.09 | <0.001 | >0.05 |

**Table S4.3.** Raw model evaluation outputs from ENMeval (Muscarella et al. 2014) of the final models using all occurrences available (D*_n_* + D*_c_* + D*_s_*).

| **Features** | **RM** | **Full AUC** | **Mean AUC** | **Var AUC** | **Mean AUC**  **DIFF** | **Var AUC**  **DIFF** | **Mean**  **OR10** | **Var OR10** | **Mean**  **ORmin** | **Var ORmin** | **AICc** | **Delta**  **AICc** | **# Par** |
| --- | --- | --- | --- | --- | --- | --- | --- | --- | --- | --- | --- | --- | --- |
| L | 0.5 | 0.8064 | 0.800833 | 1.564904 | 0.060466 | 0.559499 | 0.106796 | 0.096326 | 0.009709 | 0.009709 | 2559.713 | 439.7675 | 3 |
| Q | 0.5 | 0.8967 | 0.895609 | 2.270158 | 0.050571 | 1.629139 | 0.106796 | 0.096326 | 0.009709 | 0.009709 | 2436.734 | 316.7877 | 3 |
| H | 0.5 | 0.9792 | 0.976022 | 0.445704 | 0.014269 | 0.415131 | 0.106796 | 0.096326 | 0.009709 | 0.009709 | 3065.729 | 945.7829 | 85 |
| P | 0.5 | 0.7549 | 0.754321 | 6.133358 | 0.117958 | 2.249614 | 0.106796 | 0.096326 | 0.009709 | 0.009709 | 2553.062 | 433.1158 | 3 |
| T | 0.5 | 0.9785 | 0.955973 | 1.751469 | 0.034085 | 1.676121 | 0.165049 | 0.139159 | 0.009709 | 0.009709 | 2613.922 | 493.9761 | 75 |
| LQ | 0.5 | 0.9459 | 0.944762 | 1.135217 | 0.027909 | 0.965198 | 0.106796 | 0.096326 | 0.009709 | 0.009709 | 2310.91 | 190.9645 | 6 |
| LH | 0.5 | 0.9795 | 0.975994 | 0.447052 | 0.014313 | 0.416317 | 0.106796 | 0.096326 | 0.009709 | 0.009709 | 2704.329 | 584.3826 | 78 |
| LP | 0.5 | 0.89 | 0.887909 | 1.15416 | 0.048451 | 0.510127 | 0.106796 | 0.096326 | 0.009709 | 0.009709 | 2443.424 | 323.4778 | 6 |
| LT | 0.5 | 0.9785 | 0.955973 | 1.751469 | 0.034085 | 1.676121 | 0.165049 | 0.139159 | 0.009709 | 0.009709 | 2613.922 | 493.9761 | 75 |
| QH | 0.5 | 0.9793 | 0.975577 | 0.458759 | 0.014444 | 0.428217 | 0.106796 | 0.096326 | 0.009709 | 0.009709 | 2416.02 | 296.0738 | 66 |
| QP | 0.5 | 0.9099 | 0.907473 | 2.359163 | 0.05054 | 1.811429 | 0.106796 | 0.096326 | 0.009709 | 0.009709 | 2412.5 | 292.5542 | 6 |
| QT | 0.5 | 0.9794 | 0.957235 | 1.619609 | 0.03314 | 1.551376 | 0.165049 | 0.139159 | 0.009709 | 0.009709 | 2620.025 | 500.079 | 75 |
| HP | 0.5 | 0.9795 | 0.976401 | 0.424116 | 0.013945 | 0.394768 | 0.106796 | 0.096326 | 0.009709 | 0.009709 | 2822.292 | 702.3464 | 81 |
| HT | 0.5 | 0.9822 | 0.962794 | 1.063416 | 0.028343 | 1.014615 | 0.165049 | 0.139159 | 0.009709 | 0.009709 | 2527.219 | 407.2729 | 72 |
| PT | 0.5 | 0.9785 | 0.955972 | 1.751461 | 0.034085 | 1.676121 | 0.165049 | 0.139159 | 0.009709 | 0.009709 | 2613.922 | 493.9761 | 75 |
| LQH | 0.5 | 0.9793 | 0.975482 | 0.471393 | 0.014566 | 0.440679 | 0.106796 | 0.096326 | 0.009709 | 0.009709 | 2278.763 | 158.8173 | 55 |
| LQP | 0.5 | 0.9554 | 0.953167 | 0.790971 | 0.024297 | 0.67021 | 0.106796 | 0.096326 | 0.009709 | 0.009709 | 2249.69 | 129.7445 | 9 |
| LQT | 0.5 | 0.9794 | 0.957235 | 1.619609 | 0.03314 | 1.551376 | 0.165049 | 0.139159 | 0.009709 | 0.009709 | 2620.025 | 500.079 | 75 |
| LHP | 0.5 | 0.9799 | 0.976314 | 0.42894 | 0.013964 | 0.399843 | 0.106796 | 0.096326 | 0.009709 | 0.009709 | 2458.736 | 338.7898 | 69 |
| LHT | 0.5 | 0.9822 | 0.962794 | 1.063416 | 0.028343 | 1.014615 | 0.165049 | 0.139159 | 0.009709 | 0.009709 | 2527.219 | 407.2729 | 72 |
| LPT | 0.5 | 0.9785 | 0.955972 | 1.751461 | 0.034085 | 1.676121 | 0.165049 | 0.139159 | 0.009709 | 0.009709 | 2613.922 | 493.9761 | 75 |
| QHP | 0.5 | 0.9802 | 0.976473 | 0.407314 | 0.013781 | 0.378967 | 0.106796 | 0.096326 | 0.009709 | 0.009709 | 2661.873 | 541.9274 | 77 |
| QHT | 0.5 | 0.9822 | 0.962794 | 1.063416 | 0.028343 | 1.014615 | 0.165049 | 0.139159 | 0.009709 | 0.009709 | 2527.219 | 407.2729 | 72 |
| QPT | 0.5 | 0.9794 | 0.957235 | 1.619609 | 0.03314 | 1.551376 | 0.165049 | 0.139159 | 0.009709 | 0.009709 | 2620.025 | 500.079 | 75 |
| HPT | 0.5 | 0.9822 | 0.962792 | 1.063405 | 0.028343 | 1.014615 | 0.165049 | 0.139159 | 0.009709 | 0.009709 | 2527.219 | 407.2729 | 72 |
| LQHP | 0.5 | 0.9801 | 0.976275 | 0.429805 | 0.013978 | 0.401124 | 0.106796 | 0.096326 | 0.009709 | 0.009709 | 2359.979 | 240.0329 | 63 |
| LQHT | 0.5 | 0.9822 | 0.962794 | 1.063416 | 0.028343 | 1.014615 | 0.165049 | 0.139159 | 0.009709 | 0.009709 | 2527.219 | 407.2729 | 72 |
| LQPT | 0.5 | 0.9794 | 0.957235 | 1.619609 | 0.03314 | 1.551376 | 0.165049 | 0.139159 | 0.009709 | 0.009709 | 2620.025 | 500.079 | 75 |
| LHPT | 0.5 | 0.9822 | 0.962792 | 1.063405 | 0.028343 | 1.014615 | 0.165049 | 0.139159 | 0.009709 | 0.009709 | 2527.219 | 407.2729 | 72 |
| QHPT | 0.5 | 0.9822 | 0.96279 | 1.063391 | 0.028343 | 1.014615 | 0.165049 | 0.139159 | 0.009709 | 0.009709 | 2527.219 | 407.2729 | 72 |
| LQHPT | 0.5 | 0.9822 | 0.96279 | 1.063391 | 0.028343 | 1.014615 | 0.165049 | 0.139159 | 0.009709 | 0.009709 | 2527.219 | 407.2729 | 72 |
| L | 1 | 0.8064 | 0.800837 | 1.574355 | 0.060688 | 0.563392 | 0.106796 | 0.096326 | 0.009709 | 0.009709 | 2559.714 | 439.7683 | 3 |
| Q | 1 | 0.8966 | 0.89559 | 2.269841 | 0.050578 | 1.628525 | 0.106796 | 0.096326 | 0.009709 | 0.009709 | 2436.725 | 316.779 | 3 |
| H | 1 | 0.9767 | 0.974593 | 0.489915 | 0.014889 | 0.452674 | 0.106796 | 0.096326 | 0.009709 | 0.009709 | 4482.584 | 2362.638 | 94 |
| P | 1 | 0.7549 | 0.754321 | 6.133358 | 0.117958 | 2.249614 | 0.106796 | 0.096326 | 0.009709 | 0.009709 | 2553.062 | 433.1158 | 3 |
| T | 1 | 0.9766 | 0.957582 | 1.648864 | 0.031755 | 1.574322 | 0.184466 | 0.151913 | 0.009709 | 0.009709 | 2173.102 | 53.15574 | 50 |
| LQ | 1 | 0.9459 | 0.94476 | 1.135532 | 0.027912 | 0.965573 | 0.106796 | 0.096326 | 0.009709 | 0.009709 | 2310.935 | 190.9886 | 6 |
| LH | 1 | 0.9767 | 0.974582 | 0.491656 | 0.014913 | 0.454319 | 0.106796 | 0.096326 | 0.009709 | 0.009709 | NA | NA | 102 |
| LP | 1 | 0.8899 | 0.887832 | 1.157562 | 0.048541 | 0.511079 | 0.106796 | 0.096326 | 0.009709 | 0.009709 | 2443.436 | 323.4905 | 6 |
| LT | 1 | 0.9771 | 0.958465 | 1.576088 | 0.030948 | 1.505424 | 0.194175 | 0.158005 | 0.009709 | 0.009709 | 2188.334 | 68.38788 | 52 |
| QH | 1 | 0.9763 | 0.972891 | 0.757783 | 0.016258 | 0.717658 | 0.106796 | 0.096326 | 0.009709 | 0.009709 | 2397.276 | 277.3301 | 63 |
| QP | 1 | 0.9099 | 0.907484 | 2.357746 | 0.050517 | 1.810771 | 0.106796 | 0.096326 | 0.009709 | 0.009709 | 2412.47 | 292.5241 | 6 |
| QT | 1 | 0.9748 | 0.960679 | 1.376384 | 0.02876 | 1.296969 | 0.165049 | 0.139159 | 0.009709 | 0.009709 | 2135.219 | 15.27326 | 41 |
| HP | 1 | 0.9773 | 0.974649 | 0.464033 | 0.014816 | 0.427726 | 0.116505 | 0.103941 | 0.009709 | 0.009709 | NA | NA | 102 |
| HT | 1 | 0.9775 | 0.963335 | 1.23552 | 0.02658 | 1.174762 | 0.174757 | 0.145631 | 0.009709 | 0.009709 | 4108.782 | 1988.836 | 93 |
| PT | 1 | 0.9777 | 0.961058 | 1.31323 | 0.028915 | 1.24517 | 0.184466 | 0.151913 | 0.009709 | 0.009709 | 2162.013 | 42.06752 | 47 |
| LQH | 1 | 0.9762 | 0.972716 | 0.768211 | 0.016317 | 0.727883 | 0.106796 | 0.096326 | 0.009709 | 0.009709 | 2397.679 | 277.7326 | 63 |
| LQP | 1 | 0.9552 | 0.953035 | 0.799563 | 0.024384 | 0.677776 | 0.106796 | 0.096326 | 0.009709 | 0.009709 | 2248.399 | 128.4527 | 8 |
| LQT | 1 | 0.9749 | 0.961114 | 1.339105 | 0.028339 | 1.261925 | 0.15534 | 0.132496 | 0.009709 | 0.009709 | 2134.003 | 14.05725 | 41 |
| LHP | 1 | 0.9778 | 0.975408 | 0.452482 | 0.0141 | 0.418329 | 0.116505 | 0.103941 | 0.009709 | 0.009709 | 2180.457 | 60.51129 | 39 |
| LHT | 1 | 0.9774 | 0.963332 | 1.235695 | 0.026586 | 1.174957 | 0.174757 | 0.145631 | 0.009709 | 0.009709 | 5976.576 | 3856.631 | 97 |
| LPT | 1 | 0.9776 | 0.962253 | 1.246133 | 0.027842 | 1.17667 | 0.174757 | 0.145631 | 0.009709 | 0.009709 | 2135.308 | 15.3622 | 41 |
| QHP | 1 | 0.977 | 0.973404 | 0.631623 | 0.015773 | 0.592524 | 0.116505 | 0.103941 | 0.009709 | 0.009709 | 3160.513 | 1040.567 | 86 |
| QHT | 1 | 0.9761 | 0.964551 | 1.147034 | 0.025195 | 1.085743 | 0.165049 | 0.139159 | 0.009709 | 0.009709 | 2174.971 | 55.02537 | 48 |
| QPT | 1 | 0.9752 | 0.96128 | 1.396612 | 0.028392 | 1.320174 | 0.15534 | 0.132496 | 0.009709 | 0.009709 | 2132.911 | 12.96462 | 41 |
| HPT | 1 | 0.979 | 0.96648 | 0.944684 | 0.024189 | 0.890409 | 0.165049 | 0.139159 | 0.009709 | 0.009709 | 2827.828 | 707.8822 | 82 |
| LQHP | 1 | 0.9783 | 0.975062 | 0.52102 | 0.014393 | 0.487469 | 0.116505 | 0.103941 | 0.009709 | 0.009709 | 2224.263 | 104.3168 | 47 |
| LQHT | 1 | 0.9762 | 0.964551 | 1.146797 | 0.025202 | 1.085373 | 0.165049 | 0.139159 | 0.009709 | 0.009709 | 2197.653 | 77.70666 | 51 |
| LQPT | 1 | 0.9764 | 0.962593 | 1.30337 | 0.027416 | 1.232174 | 0.145631 | 0.125642 | 0.009709 | 0.009709 | 2131.225 | 11.27886 | 41 |
| LHPT | 1 | 0.9792 | 0.96745 | 0.900575 | 0.023334 | 0.849869 | 0.174757 | 0.145631 | 0.009709 | 0.009709 | 2275.67 | 155.7239 | 60 |
| QHPT | 1 | 0.9771 | 0.965222 | 1.148498 | 0.024934 | 1.090019 | 0.145631 | 0.125642 | 0.009709 | 0.009709 | 2202.562 | 82.61575 | 52 |
| LQHPT | 1 | 0.9779 | 0.966314 | 1.026133 | 0.023962 | 0.97063 | 0.145631 | 0.125642 | 0.009709 | 0.009709 | 2176.972 | 57.02585 | 49 |
| L | 1.5 | 0.8064 | 0.800881 | 1.604631 | 0.061388 | 0.575654 | 0.106796 | 0.096326 | 0.009709 | 0.009709 | 2559.715 | 439.7689 | 3 |
| Q | 1.5 | 0.8966 | 0.89555 | 2.269753 | 0.050596 | 1.627642 | 0.106796 | 0.096326 | 0.009709 | 0.009709 | 2436.704 | 316.7582 | 3 |
| H | 1.5 | 0.9749 | 0.972871 | 0.539331 | 0.015942 | 0.494998 | 0.116505 | 0.103941 | 0.009709 | 0.009709 | NA | NA | 145 |
| P | 1.5 | 0.7549 | 0.75432 | 6.133323 | 0.117958 | 2.249616 | 0.106796 | 0.096326 | 0.009709 | 0.009709 | 2553.062 | 433.116 | 3 |
| T | 1.5 | 0.9759 | 0.95614 | 1.715875 | 0.03259 | 1.636179 | 0.184466 | 0.151913 | 0.009709 | 0.009709 | 2149.061 | 29.11553 | 39 |
| LQ | 1.5 | 0.9459 | 0.944778 | 1.138886 | 0.027946 | 0.968588 | 0.106796 | 0.096326 | 0.009709 | 0.009709 | 2311.037 | 191.0913 | 6 |
| LH | 1.5 | 0.9749 | 0.972823 | 0.532855 | 0.015961 | 0.488558 | 0.116505 | 0.103941 | 0.009709 | 0.009709 | NA | NA | 145 |
| LP | 1.5 | 0.8895 | 0.887471 | 1.17065 | 0.048885 | 0.515036 | 0.106796 | 0.096326 | 0.009709 | 0.009709 | 2443.498 | 323.5517 | 6 |
| LT | 1.5 | 0.9759 | 0.957432 | 1.584747 | 0.031342 | 1.508154 | 0.184466 | 0.151913 | 0.009709 | 0.009709 | 2172.147 | 52.20134 | 43 |
| QH | 1.5 | 0.9726 | 0.970367 | 0.856252 | 0.017542 | 0.803021 | 0.106796 | 0.096326 | 0.009709 | 0.009709 | 2468.94 | 348.994 | 66 |
| QP | 1.5 | 0.91 | 0.907546 | 2.353459 | 0.050433 | 1.808674 | 0.106796 | 0.096326 | 0.009709 | 0.009709 | 2412.433 | 292.487 | 6 |
| QT | 1.5 | 0.9735 | 0.961641 | 1.225634 | 0.027007 | 1.147304 | 0.145631 | 0.125642 | 0.009709 | 0.009709 | 2124.875 | 4.929095 | 30 |
| HP | 1.5 | 0.9754 | 0.972666 | 0.490416 | 0.015899 | 0.446903 | 0.106796 | 0.096326 | 0.009709 | 0.009709 | NA | NA | 113 |
| HT | 1.5 | 0.9754 | 0.965333 | 1.091999 | 0.023946 | 1.031534 | 0.145631 | 0.125642 | 0.009709 | 0.009709 | 5327.386 | 3207.44 | 96 |
| PT | 1.5 | 0.9762 | 0.960552 | 1.255594 | 0.028703 | 1.183653 | 0.15534 | 0.132496 | 0.009709 | 0.009709 | 2141.75 | 21.80382 | 37 |
| LQH | 1.5 | 0.9722 | 0.970502 | 0.832462 | 0.017379 | 0.779612 | 0.106796 | 0.096326 | 0.009709 | 0.009709 | 2275.313 | 155.3671 | 48 |
| LQP | 1.5 | 0.9551 | 0.952801 | 0.824206 | 0.024543 | 0.70095 | 0.106796 | 0.096326 | 0.009709 | 0.009709 | 2252.604 | 132.6586 | 9 |
| LQT | 1.5 | 0.9736 | 0.961603 | 1.225238 | 0.02703 | 1.147263 | 0.145631 | 0.125642 | 0.009709 | 0.009709 | 2128.815 | 8.869149 | 31 |
| LHP | 1.5 | 0.9758 | 0.973353 | 0.478315 | 0.015263 | 0.437017 | 0.106796 | 0.096326 | 0.009709 | 0.009709 | 2188.657 | 68.71127 | 37 |
| LHT | 1.5 | 0.9754 | 0.965336 | 1.091755 | 0.023944 | 1.031258 | 0.145631 | 0.125642 | 0.009709 | 0.009709 | 5327.386 | 3207.44 | 96 |
| LPT | 1.5 | 0.9768 | 0.963112 | 1.062433 | 0.026324 | 0.996251 | 0.145631 | 0.125642 | 0.009709 | 0.009709 | 2127.497 | 7.550768 | 33 |
| QHP | 1.5 | 0.9736 | 0.969762 | 0.858474 | 0.018332 | 0.804657 | 0.106796 | 0.096326 | 0.009709 | 0.009709 | 2786.618 | 666.6717 | 79 |
| QHT | 1.5 | 0.9749 | 0.966199 | 1.022152 | 0.022866 | 0.962735 | 0.15534 | 0.132496 | 0.009709 | 0.009709 | 2328.877 | 208.9313 | 60 |
| QPT | 1.5 | 0.9737 | 0.961301 | 1.282642 | 0.027417 | 1.203831 | 0.135922 | 0.118599 | 0.009709 | 0.009709 | 2129.981 | 10.03484 | 33 |
| HPT | 1.5 | 0.9774 | 0.966397 | 0.904882 | 0.023316 | 0.8498 | 0.145631 | 0.125642 | 0.009709 | 0.009709 | 2729.284 | 609.3378 | 79 |
| LQHP | 1.5 | 0.9757 | 0.972581 | 0.60554 | 0.015987 | 0.562026 | 0.116505 | 0.103941 | 0.009709 | 0.009709 | 2203.251 | 83.30528 | 39 |
| LQHT | 1.5 | 0.9749 | 0.966223 | 1.022106 | 0.022832 | 0.962721 | 0.145631 | 0.125642 | 0.009709 | 0.009709 | 2341.186 | 221.2405 | 61 |
| LQPT | 1.5 | 0.9763 | 0.963128 | 1.183669 | 0.026192 | 1.114953 | 0.126214 | 0.111365 | 0.009709 | 0.009709 | 2128.07 | 8.124409 | 34 |
| LHPT | 1.5 | 0.978 | 0.967865 | 0.834812 | 0.021922 | 0.784325 | 0.145631 | 0.125642 | 0.009709 | 0.009709 | 2215.753 | 95.80725 | 49 |
| QHPT | 1.5 | 0.9757 | 0.965443 | 1.061365 | 0.023688 | 1.002004 | 0.135922 | 0.118599 | 0.009709 | 0.009709 | 2306.022 | 186.0763 | 59 |
| LQHPT | 1.5 | 0.9775 | 0.967666 | 0.895116 | 0.021847 | 0.84556 | 0.116505 | 0.103941 | 0.009709 | 0.009709 | 2167.887 | 47.9414 | 43 |
| L | 2 | 0.8065 | 0.800879 | 1.634799 | 0.062085 | 0.587706 | 0.106796 | 0.096326 | 0.009709 | 0.009709 | 2559.723 | 439.7773 | 3 |
| Q | 2 | 0.8966 | 0.895485 | 2.269972 | 0.05064 | 1.627054 | 0.106796 | 0.096326 | 0.009709 | 0.009709 | 2436.699 | 316.7534 | 3 |
| H | 2 | 0.973 | 0.970575 | 0.601332 | 0.017328 | 0.548763 | 0.106796 | 0.096326 | 0.009709 | 0.009709 | NA | NA | 130 |
| P | 2 | 0.7549 | 0.754312 | 6.133631 | 0.117969 | 2.249774 | 0.106796 | 0.096326 | 0.009709 | 0.009709 | 2553.065 | 433.119 | 3 |
| T | 2 | 0.975 | 0.955258 | 1.719246 | 0.032602 | 1.637756 | 0.184466 | 0.151913 | 0.009709 | 0.009709 | 2171.466 | 51.51978 | 36 |
| LQ | 2 | 0.9459 | 0.944783 | 1.141558 | 0.02799 | 0.970898 | 0.106796 | 0.096326 | 0.009709 | 0.009709 | 2311.189 | 191.2431 | 6 |
| LH | 2 | 0.973 | 0.970665 | 0.599468 | 0.017284 | 0.547003 | 0.106796 | 0.096326 | 0.009709 | 0.009709 | NA | NA | 130 |
| LP | 2 | 0.8892 | 0.887111 | 1.184553 | 0.049249 | 0.519487 | 0.106796 | 0.096326 | 0.009709 | 0.009709 | 2443.615 | 323.6687 | 6 |
| LT | 2 | 0.9742 | 0.956954 | 1.579699 | 0.030899 | 1.499079 | 0.174757 | 0.145631 | 0.009709 | 0.009709 | 2171.897 | 51.95127 | 36 |
| QH | 2 | 0.9694 | 0.967558 | 0.912786 | 0.019088 | 0.845941 | 0.116505 | 0.103941 | 0.009709 | 0.009709 | 2409.618 | 289.6718 | 60 |
| QP | 2 | 0.9101 | 0.907612 | 2.351084 | 0.050383 | 1.807621 | 0.106796 | 0.096326 | 0.009709 | 0.009709 | 2412.427 | 292.4814 | 6 |
| QT | 2 | 0.9722 | 0.961457 | 1.195329 | 0.026326 | 1.113215 | 0.135922 | 0.118599 | 0.009709 | 0.009709 | 2141.83 | 21.88444 | 25 |
| HP | 2 | 0.9731 | 0.970378 | 0.549587 | 0.017289 | 0.497018 | 0.106796 | 0.096326 | 0.009709 | 0.009709 | NA | NA | 105 |
| HT | 2 | 0.974 | 0.964742 | 1.134517 | 0.024303 | 1.067335 | 0.145631 | 0.125642 | 0.009709 | 0.009709 | 2765.394 | 645.4483 | 79 |
| PT | 2 | 0.9747 | 0.9595 | 1.254313 | 0.028863 | 1.177721 | 0.174757 | 0.145631 | 0.009709 | 0.009709 | 2155.778 | 35.83193 | 33 |
| LQH | 2 | 0.9696 | 0.968147 | 0.881059 | 0.01878 | 0.816395 | 0.106796 | 0.096326 | 0.009709 | 0.009709 | 2223.826 | 103.8798 | 35 |
| LQP | 2 | 0.954 | 0.951729 | 0.871137 | 0.025279 | 0.741403 | 0.116505 | 0.103941 | 0.009709 | 0.009709 | 2257.754 | 137.8086 | 9 |
| LQT | 2 | 0.9722 | 0.961573 | 1.174149 | 0.026208 | 1.092316 | 0.135922 | 0.118599 | 0.009709 | 0.009709 | 2141.83 | 21.88444 | 25 |
| LHP | 2 | 0.9744 | 0.971687 | 0.493465 | 0.016085 | 0.447649 | 0.106796 | 0.096326 | 0.009709 | 0.009709 | 2199.198 | 79.25182 | 36 |
| LHT | 2 | 0.9742 | 0.964648 | 1.146396 | 0.02441 | 1.079318 | 0.145631 | 0.125642 | 0.009709 | 0.009709 | 2902.013 | 782.0671 | 82 |
| LPT | 2 | 0.9762 | 0.962931 | 0.997539 | 0.026043 | 0.929036 | 0.15534 | 0.132496 | 0.009709 | 0.009709 | 2121.944 | 1.997624 | 25 |
| QHP | 2 | 0.9704 | 0.966823 | 0.935241 | 0.019951 | 0.866356 | 0.106796 | 0.096326 | 0.009709 | 0.009709 | 2423.301 | 303.3551 | 62 |
| QHT | 2 | 0.9733 | 0.965588 | 1.030356 | 0.022752 | 0.964415 | 0.135922 | 0.118599 | 0.009709 | 0.009709 | 2241.803 | 121.8571 | 47 |
| QPT | 2 | 0.9719 | 0.961151 | 1.243568 | 0.02667 | 1.159844 | 0.126214 | 0.111365 | 0.009709 | 0.009709 | 2136.525 | 16.57901 | 26 |
| HPT | 2 | 0.9759 | 0.96488 | 0.995169 | 0.024291 | 0.932265 | 0.145631 | 0.125642 | 0.009709 | 0.009709 | 2950.18 | 830.2343 | 83 |
| LQHP | 2 | 0.9746 | 0.9718 | 0.621433 | 0.015951 | 0.576491 | 0.116505 | 0.103941 | 0.009709 | 0.009709 | 2158.773 | 38.8273 | 24 |
| LQHT | 2 | 0.9732 | 0.965596 | 1.021246 | 0.022725 | 0.95517 | 0.135922 | 0.118599 | 0.009709 | 0.009709 | 2256.013 | 136.0674 | 49 |
| LQPT | 2 | 0.9763 | 0.964001 | 1.097806 | 0.024726 | 1.032494 | 0.126214 | 0.111365 | 0.009709 | 0.009709 | 2119.946 | 0 | 24 |
| LHPT | 2 | 0.9774 | 0.967593 | 0.794497 | 0.021774 | 0.741691 | 0.165049 | 0.139159 | 0.009709 | 0.009709 | 2163.327 | 43.38075 | 37 |
| QHPT | 2 | 0.973 | 0.964718 | 1.074119 | 0.023386 | 1.005874 | 0.135922 | 0.118599 | 0.009709 | 0.009709 | 2237.522 | 117.5764 | 48 |
| LQHPT | 2 | 0.9759 | 0.967501 | 0.942294 | 0.021504 | 0.888125 | 0.126214 | 0.111365 | 0.009709 | 0.009709 | 2148.669 | 28.72299 | 33 |
| L | 2.5 | 0.8064 | 0.800916 | 1.664934 | 0.062755 | 0.599634 | 0.106796 | 0.096326 | 0.009709 | 0.009709 | 2559.739 | 439.7935 | 3 |
| Q | 2.5 | 0.8965 | 0.89546 | 2.270366 | 0.050679 | 1.62619 | 0.106796 | 0.096326 | 0.009709 | 0.009709 | 2436.698 | 316.7521 | 3 |
| H | 2.5 | 0.9708 | 0.968338 | 0.66394 | 0.018673 | 0.60111 | 0.116505 | 0.103941 | 0.009709 | 0.009709 | NA | NA | 119 |
| P | 2.5 | 0.7549 | 0.754315 | 6.133248 | 0.117966 | 2.249578 | 0.106796 | 0.096326 | 0.009709 | 0.009709 | 2553.069 | 433.1232 | 3 |
| T | 2.5 | 0.9747 | 0.953844 | 1.795407 | 0.033501 | 1.710703 | 0.174757 | 0.145631 | 0.009709 | 0.009709 | 2171.523 | 51.57714 | 27 |
| LQ | 2.5 | 0.9459 | 0.944761 | 1.14374 | 0.028028 | 0.972979 | 0.106796 | 0.096326 | 0.009709 | 0.009709 | 2311.367 | 191.4208 | 6 |
| LH | 2.5 | 0.9709 | 0.968311 | 0.666424 | 0.018729 | 0.603154 | 0.116505 | 0.103941 | 0.009709 | 0.009709 | NA | NA | 116 |
| LP | 2.5 | 0.8888 | 0.886724 | 1.198776 | 0.049627 | 0.52399 | 0.106796 | 0.096326 | 0.009709 | 0.009709 | 2443.743 | 323.7968 | 6 |
| LT | 2.5 | 0.9732 | 0.956038 | 1.52123 | 0.03071 | 1.440386 | 0.184466 | 0.151913 | 0.009709 | 0.009709 | 2198.032 | 78.08586 | 34 |
| QH | 2.5 | 0.9661 | 0.964255 | 0.970053 | 0.020766 | 0.888822 | 0.116505 | 0.103941 | 0.009709 | 0.009709 | 2487.911 | 367.9648 | 64 |
| QP | 2.5 | 0.9101 | 0.907671 | 2.348233 | 0.050332 | 1.806262 | 0.106796 | 0.096326 | 0.009709 | 0.009709 | 2412.424 | 292.478 | 6 |
| QT | 2.5 | 0.9703 | 0.960294 | 1.198052 | 0.026463 | 1.107439 | 0.135922 | 0.118599 | 0.009709 | 0.009709 | 2148.206 | 28.2602 | 18 |
| HP | 2.5 | 0.9706 | 0.967906 | 0.598839 | 0.018765 | 0.534065 | 0.106796 | 0.096326 | 0.009709 | 0.009709 | NA | NA | 119 |
| HT | 2.5 | 0.9732 | 0.963494 | 1.186579 | 0.024937 | 1.115243 | 0.15534 | 0.132496 | 0.009709 | 0.009709 | 2983.028 | 863.0821 | 83 |
| PT | 2.5 | 0.9738 | 0.958794 | 1.238551 | 0.028913 | 1.158837 | 0.165049 | 0.139159 | 0.009709 | 0.009709 | 2156.626 | 36.6805 | 25 |
| LQH | 2.5 | 0.9673 | 0.966776 | 0.903302 | 0.019436 | 0.833917 | 0.106796 | 0.096326 | 0.009709 | 0.009709 | 2198.516 | 78.5705 | 23 |
| LQP | 2.5 | 0.9531 | 0.95071 | 0.920046 | 0.025949 | 0.784304 | 0.116505 | 0.103941 | 0.009709 | 0.009709 | 2262.365 | 142.4191 | 9 |
| LQT | 2.5 | 0.9703 | 0.960364 | 1.157778 | 0.02636 | 1.067756 | 0.135922 | 0.118599 | 0.009709 | 0.009709 | 2151.519 | 31.57315 | 19 |
| LHP | 2.5 | 0.9726 | 0.970135 | 0.493962 | 0.016802 | 0.441228 | 0.106796 | 0.096326 | 0.009709 | 0.009709 | 2203.774 | 83.82799 | 33 |
| LHT | 2.5 | 0.9732 | 0.963322 | 1.194911 | 0.025107 | 1.123336 | 0.15534 | 0.132496 | 0.009709 | 0.009709 | 2983.028 | 863.0821 | 83 |
| LPT | 2.5 | 0.9749 | 0.961254 | 1.037664 | 0.026886 | 0.96388 | 0.145631 | 0.125642 | 0.009709 | 0.009709 | 2146.359 | 26.41318 | 25 |
| QHP | 2.5 | 0.9654 | 0.962702 | 1.02646 | 0.022171 | 0.935068 | 0.116505 | 0.103941 | 0.009709 | 0.009709 | 2417.202 | 297.2559 | 59 |
| QHT | 2.5 | 0.9713 | 0.963995 | 1.063298 | 0.023376 | 0.987519 | 0.126214 | 0.111365 | 0.009709 | 0.009709 | 2210.185 | 90.23943 | 37 |
| QPT | 2.5 | 0.9701 | 0.960089 | 1.228332 | 0.026747 | 1.136481 | 0.126214 | 0.111365 | 0.009709 | 0.009709 | 2153.003 | 33.05708 | 22 |
| HPT | 2.5 | 0.9741 | 0.963166 | 1.083696 | 0.025091 | 1.014228 | 0.145631 | 0.125642 | 0.009709 | 0.009709 | 2422.44 | 302.4943 | 64 |
| LQHP | 2.5 | 0.9752 | 0.972664 | 0.595254 | 0.015157 | 0.552757 | 0.126214 | 0.111365 | 0.009709 | 0.009709 | 2147.399 | 27.45357 | 21 |
| LQHT | 2.5 | 0.9713 | 0.964241 | 1.026818 | 0.023132 | 0.951622 | 0.126214 | 0.111365 | 0.009709 | 0.009709 | 2195.94 | 75.99402 | 34 |
| LQPT | 2.5 | 0.9748 | 0.963836 | 1.032473 | 0.024097 | 0.964471 | 0.135922 | 0.118599 | 0.009709 | 0.009709 | 2137.537 | 17.59078 | 22 |
| LHPT | 2.5 | 0.976 | 0.965672 | 0.837175 | 0.022787 | 0.778276 | 0.145631 | 0.125642 | 0.009709 | 0.009709 | 2164.469 | 44.52283 | 32 |
| QHPT | 2.5 | 0.9707 | 0.962985 | 1.108407 | 0.024096 | 1.029011 | 0.126214 | 0.111365 | 0.009709 | 0.009709 | 2233.812 | 113.8663 | 43 |
| LQHPT | 2.5 | 0.9747 | 0.967086 | 0.890695 | 0.021158 | 0.831774 | 0.116505 | 0.103941 | 0.009709 | 0.009709 | 2172.785 | 52.8388 | 32 |
| L | 3 | 0.8064 | 0.800945 | 1.695398 | 0.063421 | 0.611491 | 0.106796 | 0.096326 | 0.009709 | 0.009709 | 2559.763 | 439.8174 | 3 |
| Q | 3 | 0.8965 | 0.895439 | 2.270609 | 0.050706 | 1.625395 | 0.106796 | 0.096326 | 0.009709 | 0.009709 | 2436.7 | 316.7538 | 3 |
| H | 3 | 0.9682 | 0.965632 | 0.742965 | 0.020396 | 0.666075 | 0.126214 | 0.111365 | 0.009709 | 0.009709 | NA | NA | 115 |
| P | 3 | 0.7549 | 0.754319 | 6.132423 | 0.117958 | 2.249301 | 0.106796 | 0.096326 | 0.009709 | 0.009709 | 2553.074 | 433.1285 | 3 |
| T | 3 | 0.9744 | 0.954023 | 1.605979 | 0.033025 | 1.520542 | 0.203883 | 0.163906 | 0.009709 | 0.009709 | 2208.207 | 88.26134 | 26 |
| LQ | 3 | 0.9459 | 0.944738 | 1.146574 | 0.02807 | 0.975527 | 0.106796 | 0.096326 | 0.009709 | 0.009709 | 2311.568 | 191.6221 | 6 |
| LH | 3 | 0.9683 | 0.965536 | 0.756444 | 0.020494 | 0.678502 | 0.126214 | 0.111365 | 0.009709 | 0.009709 | NA | NA | 113 |
| LP | 3 | 0.8884 | 0.88631 | 1.213156 | 0.050006 | 0.528734 | 0.106796 | 0.096326 | 0.009709 | 0.009709 | 2443.909 | 323.963 | 6 |
| LT | 3 | 0.9723 | 0.955126 | 1.442216 | 0.030638 | 1.359615 | 0.213592 | 0.169617 | 0.009709 | 0.009709 | 2192.545 | 72.59912 | 23 |
| QH | 3 | 0.9626 | 0.960979 | 1.026004 | 0.02249 | 0.927075 | 0.106796 | 0.096326 | 0.009709 | 0.009709 | 2338.468 | 218.522 | 46 |
| QP | 3 | 0.9102 | 0.907728 | 2.344954 | 0.050271 | 1.804618 | 0.106796 | 0.096326 | 0.009709 | 0.009709 | 2412.424 | 292.4778 | 6 |
| QT | 3 | 0.9676 | 0.958865 | 1.204812 | 0.026791 | 1.102028 | 0.126214 | 0.111365 | 0.009709 | 0.009709 | 2168.03 | 48.08441 | 15 |
| HP | 3 | 0.9679 | 0.964743 | 0.703821 | 0.020711 | 0.62296 | 0.116505 | 0.103941 | 0.009709 | 0.009709 | NA | NA | 108 |
| HT | 3 | 0.9718 | 0.96201 | 1.262854 | 0.025825 | 1.184721 | 0.15534 | 0.132496 | 0.009709 | 0.009709 | 2581.247 | 461.3011 | 71 |
| PT | 3 | 0.9725 | 0.957151 | 1.251322 | 0.029447 | 1.168494 | 0.174757 | 0.145631 | 0.009709 | 0.009709 | 2182.36 | 62.41425 | 23 |
| LQH | 3 | 0.9672 | 0.96566 | 0.92274 | 0.019983 | 0.848008 | 0.106796 | 0.096326 | 0.009709 | 0.009709 | 2193.526 | 73.58052 | 19 |
| LQP | 3 | 0.9521 | 0.949605 | 0.973053 | 0.026649 | 0.830504 | 0.106796 | 0.096326 | 0.009709 | 0.009709 | 2266.454 | 146.5085 | 9 |
| LQT | 3 | 0.9677 | 0.959046 | 1.148727 | 0.026546 | 1.047769 | 0.126214 | 0.111365 | 0.009709 | 0.009709 | 2175.063 | 55.1167 | 17 |
| LHP | 3 | 0.9712 | 0.968538 | 0.518429 | 0.017513 | 0.459686 | 0.106796 | 0.096326 | 0.009709 | 0.009709 | 2199.587 | 79.64159 | 28 |
| LHT | 3 | 0.9718 | 0.961917 | 1.270486 | 0.025921 | 1.192322 | 0.15534 | 0.132496 | 0.009709 | 0.009709 | 3071.138 | 951.1916 | 84 |
| LPT | 3 | 0.9735 | 0.959935 | 1.061524 | 0.027277 | 0.983199 | 0.15534 | 0.132496 | 0.009709 | 0.009709 | 2156.94 | 36.99454 | 21 |
| QHP | 3 | 0.9612 | 0.959139 | 1.092797 | 0.024293 | 0.979345 | 0.106796 | 0.096326 | 0.009709 | 0.009709 | 2291.823 | 171.8769 | 39 |
| QHT | 3 | 0.9687 | 0.962005 | 1.10688 | 0.024399 | 1.0163 | 0.126214 | 0.111365 | 0.009709 | 0.009709 | 2209.298 | 89.35193 | 31 |
| QPT | 3 | 0.9677 | 0.958825 | 1.227688 | 0.026902 | 1.125706 | 0.135922 | 0.118599 | 0.009709 | 0.009709 | 2156.918 | 36.97246 | 15 |
| HPT | 3 | 0.9718 | 0.961075 | 1.151373 | 0.026081 | 1.073433 | 0.145631 | 0.125642 | 0.009709 | 0.009709 | 2496.972 | 377.0265 | 67 |
| LQHP | 3 | 0.9746 | 0.971413 | 0.608678 | 0.015964 | 0.561949 | 0.116505 | 0.103941 | 0.009709 | 0.009709 | 2145.905 | 25.95877 | 19 |
| LQHT | 3 | 0.969 | 0.962537 | 1.054102 | 0.023921 | 0.966055 | 0.135922 | 0.118599 | 0.009709 | 0.009709 | 2207.129 | 87.18265 | 30 |
| LQPT | 3 | 0.9741 | 0.96377 | 0.981504 | 0.023557 | 0.912083 | 0.126214 | 0.111365 | 0.009709 | 0.009709 | 2145.026 | 25.08019 | 19 |
| LHPT | 3 | 0.9743 | 0.963395 | 0.868857 | 0.023888 | 0.802446 | 0.15534 | 0.132496 | 0.009709 | 0.009709 | 2180.606 | 60.65986 | 30 |
| QHPT | 3 | 0.9681 | 0.96084 | 1.159576 | 0.0254 | 1.063647 | 0.126214 | 0.111365 | 0.009709 | 0.009709 | 2223.329 | 103.3832 | 36 |
| LQHPT | 3 | 0.9752 | 0.966639 | 0.892951 | 0.021069 | 0.832053 | 0.126214 | 0.111365 | 0.009709 | 0.009709 | 2134.427 | 14.48081 | 20 |
| L | 3.5 | 0.8065 | 0.80092 | 1.727128 | 0.064108 | 0.624086 | 0.106796 | 0.096326 | 0.009709 | 0.009709 | 2559.796 | 439.8498 | 3 |
| Q | 3.5 | 0.8965 | 0.895381 | 2.271332 | 0.050753 | 1.62501 | 0.106796 | 0.096326 | 0.009709 | 0.009709 | 2436.704 | 316.7584 | 3 |
| H | 3.5 | 0.9648 | 0.962133 | 0.853462 | 0.022761 | 0.754592 | 0.106796 | 0.096326 | 0.009709 | 0.009709 | 2982.187 | 862.2412 | 81 |
| P | 3.5 | 0.7549 | 0.754306 | 6.132489 | 0.11797 | 2.249259 | 0.106796 | 0.096326 | 0.009709 | 0.009709 | 2553.081 | 433.1349 | 3 |
| T | 3.5 | 0.9742 | 0.952333 | 1.624392 | 0.033679 | 1.542706 | 0.203883 | 0.163906 | 0.009709 | 0.009709 | 2229.229 | 109.2827 | 21 |
| LQ | 3.5 | 0.9458 | 0.944694 | 1.150526 | 0.028143 | 0.978914 | 0.106796 | 0.096326 | 0.009709 | 0.009709 | 2311.795 | 191.8493 | 6 |
| LH | 3.5 | 0.9648 | 0.962233 | 0.827537 | 0.022608 | 0.729403 | 0.106796 | 0.096326 | 0.009709 | 0.009709 | 3733.097 | 1613.151 | 90 |
| LP | 3.5 | 0.888 | 0.885876 | 1.22755 | 0.050407 | 0.532966 | 0.106796 | 0.096326 | 0.009709 | 0.009709 | 2444.109 | 324.1636 | 6 |
| LT | 3.5 | 0.9715 | 0.954575 | 1.384193 | 0.030554 | 1.299719 | 0.213592 | 0.169617 | 0.009709 | 0.009709 | 2216.85 | 96.90373 | 20 |
| QH | 3.5 | 0.9591 | 0.957394 | 1.088305 | 0.024537 | 0.966302 | 0.106796 | 0.096326 | 0.009709 | 0.009709 | 2284.008 | 164.0616 | 31 |
| QP | 3.5 | 0.9102 | 0.907761 | 2.341772 | 0.050214 | 1.802969 | 0.106796 | 0.096326 | 0.009709 | 0.009709 | 2412.429 | 292.483 | 6 |
| QT | 3.5 | 0.9645 | 0.956933 | 1.226437 | 0.027398 | 1.108324 | 0.135922 | 0.118599 | 0.009709 | 0.009709 | 2178.704 | 58.75773 | 10 |
| HP | 3.5 | 0.965 | 0.962243 | 0.75434 | 0.022046 | 0.660509 | 0.106796 | 0.096326 | 0.009709 | 0.009709 | 4592.34 | 2472.394 | 94 |
| HT | 3.5 | 0.9697 | 0.960491 | 1.306459 | 0.026572 | 1.220011 | 0.135922 | 0.118599 | 0.009709 | 0.009709 | 2679.487 | 559.5411 | 74 |
| PT | 3.5 | 0.9704 | 0.954796 | 1.244328 | 0.030071 | 1.155244 | 0.174757 | 0.145631 | 0.009709 | 0.009709 | 2214.267 | 94.32131 | 22 |
| LQH | 3.5 | 0.9649 | 0.963466 | 0.95441 | 0.021208 | 0.868217 | 0.116505 | 0.103941 | 0.009709 | 0.009709 | 2204.54 | 84.59426 | 16 |
| LQP | 3.5 | 0.951 | 0.948682 | 0.999159 | 0.027208 | 0.849547 | 0.106796 | 0.096326 | 0.009709 | 0.009709 | 2271.149 | 151.2031 | 9 |
| LQT | 3.5 | 0.9649 | 0.957352 | 1.158161 | 0.027083 | 1.043805 | 0.126214 | 0.111365 | 0.009709 | 0.009709 | 2190.453 | 70.50673 | 13 |
| LHP | 3.5 | 0.9705 | 0.96809 | 0.539118 | 0.017708 | 0.47834 | 0.106796 | 0.096326 | 0.009709 | 0.009709 | 2188.907 | 68.96104 | 22 |
| LHT | 3.5 | 0.9697 | 0.960409 | 1.309782 | 0.026653 | 1.223385 | 0.135922 | 0.118599 | 0.009709 | 0.009709 | 3034.891 | 914.9447 | 83 |
| LPT | 3.5 | 0.9727 | 0.95959 | 1.071892 | 0.027173 | 0.992427 | 0.145631 | 0.125642 | 0.009709 | 0.009709 | 2164.42 | 44.47394 | 18 |
| QHP | 3.5 | 0.9573 | 0.955724 | 1.149494 | 0.026129 | 1.014927 | 0.106796 | 0.096326 | 0.009709 | 0.009709 | 2235.682 | 115.7366 | 19 |
| QHT | 3.5 | 0.9653 | 0.958834 | 1.175396 | 0.02606 | 1.064466 | 0.135922 | 0.118599 | 0.009709 | 0.009709 | 2193.431 | 73.48499 | 19 |
| QPT | 3.5 | 0.9644 | 0.956627 | 1.240205 | 0.027385 | 1.123472 | 0.135922 | 0.118599 | 0.009709 | 0.009709 | 2173.268 | 53.32201 | 12 |
| HPT | 3.5 | 0.9695 | 0.95906 | 1.202369 | 0.026896 | 1.114185 | 0.126214 | 0.111365 | 0.009709 | 0.009709 | 3423.488 | 1303.542 | 88 |
| LQHP | 3.5 | 0.9732 | 0.970051 | 0.665679 | 0.016899 | 0.612804 | 0.106796 | 0.096326 | 0.009709 | 0.009709 | 2152.243 | 32.29663 | 17 |
| LQHT | 3.5 | 0.966 | 0.959787 | 1.107455 | 0.025252 | 1.003438 | 0.126214 | 0.111365 | 0.009709 | 0.009709 | 2227.713 | 107.7676 | 28 |
| LQPT | 3.5 | 0.9731 | 0.963878 | 0.941325 | 0.0231 | 0.871207 | 0.145631 | 0.125642 | 0.009709 | 0.009709 | 2139.904 | 19.95824 | 13 |
| LHPT | 3.5 | 0.9732 | 0.962519 | 0.901494 | 0.024293 | 0.832257 | 0.126214 | 0.111365 | 0.009709 | 0.009709 | 2184.61 | 64.66424 | 27 |
| QHPT | 3.5 | 0.9648 | 0.957926 | 1.207842 | 0.026705 | 1.093053 | 0.126214 | 0.111365 | 0.009709 | 0.009709 | 2210.159 | 90.21302 | 27 |
| LQHPT | 3.5 | 0.9749 | 0.967047 | 0.883967 | 0.020782 | 0.824511 | 0.135922 | 0.118599 | 0.009709 | 0.009709 | 2144.605 | 24.65928 | 20 |
| L | 4 | 0.8064 | 0.800983 | 1.756719 | 0.064696 | 0.634792 | 0.106796 | 0.096326 | 0.009709 | 0.009709 | 2559.841 | 439.8955 | 3 |
| Q | 4 | 0.8964 | 0.895328 | 2.272028 | 0.050793 | 1.624836 | 0.106796 | 0.096326 | 0.009709 | 0.009709 | 2436.712 | 316.7659 | 3 |
| H | 4 | 0.9624 | 0.960497 | 0.894117 | 0.02368 | 0.784625 | 0.106796 | 0.096326 | 0.009709 | 0.009709 | 3108.377 | 988.4311 | 83 |
| P | 4 | 0.755 | 0.754348 | 6.128748 | 0.117939 | 2.248274 | 0.106796 | 0.096326 | 0.009709 | 0.009709 | 2553.098 | 433.1518 | 3 |
| T | 4 | 0.9719 | 0.953637 | 1.487235 | 0.032115 | 1.394361 | 0.213592 | 0.169617 | 0.009709 | 0.009709 | 2255.731 | 135.7847 | 18 |
| LQ | 4 | 0.9458 | 0.944647 | 1.153632 | 0.028202 | 0.981505 | 0.106796 | 0.096326 | 0.009709 | 0.009709 | 2312.074 | 192.1281 | 6 |
| LH | 4 | 0.9626 | 0.960424 | 0.873765 | 0.023642 | 0.76437 | 0.106796 | 0.096326 | 0.009709 | 0.009709 | 3108.715 | 988.7696 | 83 |
| LP | 4 | 0.8876 | 0.88554 | 1.240495 | 0.05071 | 0.536888 | 0.106796 | 0.096326 | 0.009709 | 0.009709 | 2444.367 | 324.4213 | 6 |
| LT | 4 | 0.9705 | 0.952975 | 1.327745 | 0.030851 | 1.242198 | 0.194175 | 0.158005 | 0.009709 | 0.009709 | 2253.065 | 133.1195 | 20 |
| QH | 4 | 0.9557 | 0.95461 | 1.131906 | 0.025799 | 0.994102 | 0.106796 | 0.096326 | 0.009709 | 0.009709 | 2260.996 | 141.0504 | 19 |
| QP | 4 | 0.9103 | 0.907828 | 2.33929 | 0.050148 | 1.801914 | 0.106796 | 0.096326 | 0.009709 | 0.009709 | 2412.462 | 292.5164 | 6 |
| QT | 4 | 0.961 | 0.954156 | 1.263004 | 0.028388 | 1.126737 | 0.126214 | 0.111365 | 0.009709 | 0.009709 | 2200.308 | 80.36235 | 10 |
| HP | 4 | 0.9633 | 0.960577 | 0.787181 | 0.022766 | 0.685431 | 0.106796 | 0.096326 | 0.009709 | 0.009709 | NA | NA | 113 |
| HT | 4 | 0.9676 | 0.958529 | 1.326494 | 0.02746 | 1.227595 | 0.135922 | 0.118599 | 0.009709 | 0.009709 | 2868.059 | 748.1128 | 79 |
| PT | 4 | 0.9689 | 0.953685 | 1.276199 | 0.030176 | 1.183974 | 0.174757 | 0.145631 | 0.009709 | 0.009709 | 2233.85 | 113.9044 | 19 |
| LQH | 4 | 0.9621 | 0.960784 | 0.99163 | 0.022608 | 0.89129 | 0.106796 | 0.096326 | 0.009709 | 0.009709 | 2220.657 | 100.7116 | 14 |
| LQP | 4 | 0.9499 | 0.947663 | 1.048577 | 0.02787 | 0.891235 | 0.106796 | 0.096326 | 0.009709 | 0.009709 | 2275.699 | 155.7536 | 9 |
| LQT | 4 | 0.9618 | 0.955159 | 1.17688 | 0.027697 | 1.049621 | 0.116505 | 0.103941 | 0.009709 | 0.009709 | 2212.32 | 92.37388 | 12 |
| LHP | 4 | 0.9696 | 0.967594 | 0.530372 | 0.017857 | 0.468304 | 0.106796 | 0.096326 | 0.009709 | 0.009709 | 2225.893 | 105.9467 | 29 |
| LHT | 4 | 0.9676 | 0.958495 | 1.327865 | 0.027492 | 1.229188 | 0.135922 | 0.118599 | 0.009709 | 0.009709 | 4055.657 | 1935.711 | 92 |
| LPT | 4 | 0.9722 | 0.959311 | 1.083078 | 0.02706 | 1.003134 | 0.145631 | 0.125642 | 0.009709 | 0.009709 | 2171.107 | 51.16146 | 16 |
| QHP | 4 | 0.9549 | 0.953288 | 1.173809 | 0.026913 | 1.027449 | 0.116505 | 0.103941 | 0.009709 | 0.009709 | 2245 | 125.0541 | 17 |
| QHT | 4 | 0.9607 | 0.954417 | 1.263975 | 0.028085 | 1.12597 | 0.126214 | 0.111365 | 0.009709 | 0.009709 | 2227.751 | 107.805 | 21 |
| QPT | 4 | 0.9609 | 0.953745 | 1.264687 | 0.028433 | 1.128675 | 0.126214 | 0.111365 | 0.009709 | 0.009709 | 2198.667 | 78.7213 | 13 |
| HPT | 4 | 0.9661 | 0.956398 | 1.257881 | 0.027879 | 1.154784 | 0.126214 | 0.111365 | 0.009709 | 0.009709 | 3451.7 | 1331.754 | 88 |
| LQHP | 4 | 0.971 | 0.968453 | 0.707389 | 0.017875 | 0.649024 | 0.106796 | 0.096326 | 0.009709 | 0.009709 | 2182.118 | 62.17205 | 21 |
| LQHT | 4 | 0.9625 | 0.956553 | 1.173063 | 0.026659 | 1.051251 | 0.135922 | 0.118599 | 0.009709 | 0.009709 | 2232.216 | 112.2701 | 21 |
| LQPT | 4 | 0.9719 | 0.963496 | 0.952504 | 0.023064 | 0.87845 | 0.126214 | 0.111365 | 0.009709 | 0.009709 | 2147.436 | 27.48985 | 12 |
| LHPT | 4 | 0.9726 | 0.962105 | 0.908999 | 0.024098 | 0.839564 | 0.126214 | 0.111365 | 0.009709 | 0.009709 | 2186.589 | 66.64328 | 24 |
| QHPT | 4 | 0.9603 | 0.953377 | 1.270797 | 0.028629 | 1.130233 | 0.135922 | 0.118599 | 0.009709 | 0.009709 | 2232.047 | 112.1008 | 25 |
| LQHPT | 4 | 0.9735 | 0.966053 | 0.915229 | 0.02127 | 0.849487 | 0.126214 | 0.111365 | 0.009709 | 0.009709 | 2150.905 | 30.95937 | 18 |
| L | 4.5 | 0.8065 | 0.800995 | 1.78867 | 0.065329 | 0.646663 | 0.106796 | 0.096326 | 0.009709 | 0.009709 | 2559.895 | 439.9494 | 3 |
| Q | 4.5 | 0.8964 | 0.895298 | 2.271957 | 0.050823 | 1.623699 | 0.106796 | 0.096326 | 0.009709 | 0.009709 | 2436.722 | 316.7764 | 3 |
| H | 4.5 | 0.9619 | 0.960121 | 0.904518 | 0.02391 | 0.791516 | 0.106796 | 0.096326 | 0.009709 | 0.009709 | 2794.372 | 674.4264 | 75 |
| P | 4.5 | 0.755 | 0.754402 | 6.123438 | 0.117891 | 2.246628 | 0.106796 | 0.096326 | 0.009709 | 0.009709 | 2553.119 | 433.1728 | 3 |
| T | 4.5 | 0.968 | 0.948768 | 1.453788 | 0.033063 | 1.356324 | 0.184466 | 0.151913 | 0.009709 | 0.009709 | 2285.937 | 165.9916 | 16 |
| LQ | 4.5 | 0.9458 | 0.944595 | 1.157502 | 0.02827 | 0.984853 | 0.106796 | 0.096326 | 0.009709 | 0.009709 | 2312.37 | 192.4243 | 6 |
| LH | 4.5 | 0.962 | 0.959949 | 0.891563 | 0.023908 | 0.779158 | 0.106796 | 0.096326 | 0.009709 | 0.009709 | 3122.893 | 1002.947 | 83 |
| LP | 4.5 | 0.8873 | 0.885116 | 1.257734 | 0.051101 | 0.543728 | 0.106796 | 0.096326 | 0.009709 | 0.009709 | 2444.658 | 324.7116 | 6 |
| LT | 4.5 | 0.9674 | 0.94943 | 1.25912 | 0.031487 | 1.16906 | 0.203883 | 0.163906 | 0.009709 | 0.009709 | 2289.129 | 169.1834 | 18 |
| QH | 4.5 | 0.9551 | 0.953972 | 1.14699 | 0.026051 | 1.005546 | 0.106796 | 0.096326 | 0.009709 | 0.009709 | 2269.305 | 149.359 | 20 |
| QP | 4.5 | 0.9103 | 0.907879 | 2.336943 | 0.05009 | 1.800652 | 0.106796 | 0.096326 | 0.009709 | 0.009709 | 2412.51 | 292.5639 | 6 |
| QT | 4.5 | 0.9575 | 0.951708 | 1.290536 | 0.02918 | 1.138981 | 0.116505 | 0.103941 | 0.009709 | 0.009709 | 2217.7 | 97.75426 | 9 |
| HP | 4.5 | 0.9614 | 0.958719 | 0.811839 | 0.02341 | 0.702257 | 0.106796 | 0.096326 | 0.009709 | 0.009709 | 3169.287 | 1049.342 | 84 |
| HT | 4.5 | 0.9645 | 0.955246 | 1.384014 | 0.029324 | 1.266023 | 0.116505 | 0.103941 | 0.009709 | 0.009709 | 2683.283 | 563.3371 | 72 |
| PT | 4.5 | 0.9669 | 0.951364 | 1.28639 | 0.030045 | 1.197944 | 0.174757 | 0.145631 | 0.009709 | 0.009709 | 2252.803 | 132.857 | 16 |
| LQH | 4.5 | 0.9589 | 0.957178 | 1.038328 | 0.024383 | 0.919135 | 0.106796 | 0.096326 | 0.009709 | 0.009709 | 2251.515 | 131.5694 | 17 |
| LQP | 4.5 | 0.949 | 0.946819 | 1.082751 | 0.028398 | 0.918226 | 0.106796 | 0.096326 | 0.009709 | 0.009709 | 2279.682 | 159.736 | 9 |
| LQT | 4.5 | 0.9595 | 0.953956 | 1.182973 | 0.027831 | 1.049067 | 0.126214 | 0.111365 | 0.009709 | 0.009709 | 2226.416 | 106.4697 | 10 |
| LHP | 4.5 | 0.9688 | 0.966978 | 0.541385 | 0.01816 | 0.477047 | 0.106796 | 0.096326 | 0.009709 | 0.009709 | 2226.429 | 106.4828 | 26 |
| LHT | 4.5 | 0.9645 | 0.955227 | 1.38589 | 0.029369 | 1.267961 | 0.116505 | 0.103941 | 0.009709 | 0.009709 | 2639.291 | 519.3451 | 70 |
| LPT | 4.5 | 0.9716 | 0.95897 | 1.104876 | 0.027029 | 1.023775 | 0.145631 | 0.125642 | 0.009709 | 0.009709 | 2179.954 | 60.0081 | 15 |
| QHP | 4.5 | 0.9536 | 0.952291 | 1.189385 | 0.027303 | 1.036345 | 0.116505 | 0.103941 | 0.009709 | 0.009709 | 2253.682 | 133.7364 | 17 |
| QHT | 4.5 | 0.9577 | 0.952391 | 1.299757 | 0.028849 | 1.150205 | 0.116505 | 0.103941 | 0.009709 | 0.009709 | 2224.813 | 104.8673 | 14 |
| QPT | 4.5 | 0.957 | 0.950672 | 1.296474 | 0.029695 | 1.139983 | 0.135922 | 0.118599 | 0.009709 | 0.009709 | 2220.081 | 100.1354 | 12 |
| HPT | 4.5 | 0.9619 | 0.952994 | 1.31722 | 0.02943 | 1.190732 | 0.116505 | 0.103941 | 0.009709 | 0.009709 | 2598.879 | 478.9327 | 68 |
| LQHP | 4.5 | 0.9691 | 0.966076 | 0.799721 | 0.01938 | 0.732209 | 0.106796 | 0.096326 | 0.009709 | 0.009709 | 2188.942 | 68.99567 | 19 |
| LQHT | 4.5 | 0.9592 | 0.954258 | 1.210285 | 0.027584 | 1.076023 | 0.116505 | 0.103941 | 0.009709 | 0.009709 | 2233.587 | 113.6409 | 14 |
| LQPT | 4.5 | 0.9703 | 0.962576 | 0.98433 | 0.023415 | 0.904527 | 0.126214 | 0.111365 | 0.009709 | 0.009709 | 2155.997 | 36.05154 | 11 |
| LHPT | 4.5 | 0.9719 | 0.962208 | 0.914874 | 0.023693 | 0.844437 | 0.126214 | 0.111365 | 0.009709 | 0.009709 | 2187.435 | 67.48875 | 21 |
| QHPT | 4.5 | 0.9577 | 0.951747 | 1.284762 | 0.029125 | 1.134046 | 0.135922 | 0.118599 | 0.009709 | 0.009709 | 2238.357 | 118.4111 | 21 |
| LQHPT | 4.5 | 0.9711 | 0.964551 | 0.932503 | 0.021831 | 0.861556 | 0.135922 | 0.118599 | 0.009709 | 0.009709 | 2168.055 | 48.10899 | 18 |
| L | 5 | 0.8065 | 0.80097 | 1.823453 | 0.06602 | 0.659959 | 0.106796 | 0.096326 | 0.009709 | 0.009709 | 2559.957 | 440.0115 | 3 |
| Q | 5 | 0.8963 | 0.895259 | 2.272304 | 0.050868 | 1.622687 | 0.106796 | 0.096326 | 0.009709 | 0.009709 | 2436.736 | 316.7906 | 3 |
| H | 5 | 0.9611 | 0.959531 | 0.911061 | 0.024277 | 0.793917 | 0.106796 | 0.096326 | 0.009709 | 0.009709 | 3137.193 | 1017.247 | 83 |
| P | 5 | 0.7551 | 0.754488 | 6.118659 | 0.117819 | 2.245263 | 0.106796 | 0.096326 | 0.009709 | 0.009709 | 2553.141 | 433.1956 | 3 |
| T | 5 | 0.9671 | 0.938017 | 1.398498 | 0.034111 | 1.358664 | 0.165049 | 0.139159 | 0.009709 | 0.009709 | 2321.814 | 201.868 | 14 |
| LQ | 5 | 0.9457 | 0.944592 | 1.160814 | 0.02832 | 0.987443 | 0.106796 | 0.096326 | 0.009709 | 0.009709 | 2312.683 | 192.7375 | 6 |
| LH | 5 | 0.9605 | 0.95818 | 0.908617 | 0.024847 | 0.785586 | 0.106796 | 0.096326 | 0.009709 | 0.009709 | 2653.643 | 533.6967 | 68 |
| LP | 5 | 0.8868 | 0.884751 | 1.270451 | 0.051439 | 0.546689 | 0.116505 | 0.103941 | 0.009709 | 0.009709 | 2444.98 | 325.0341 | 6 |
| LT | 5 | 0.9614 | 0.943033 | 1.176565 | 0.031894 | 1.083717 | 0.15534 | 0.132496 | 0.009709 | 0.009709 | 2325.073 | 205.1271 | 15 |
| QH | 5 | 0.9545 | 0.953366 | 1.162057 | 0.026287 | 1.017015 | 0.106796 | 0.096326 | 0.009709 | 0.009709 | 2257.383 | 137.4366 | 14 |
| QP | 5 | 0.9104 | 0.907951 | 2.333106 | 0.049999 | 1.798292 | 0.106796 | 0.096326 | 0.009709 | 0.009709 | 2412.559 | 292.6131 | 6 |
| QT | 5 | 0.957 | 0.951186 | 1.298915 | 0.029413 | 1.143642 | 0.116505 | 0.103941 | 0.009709 | 0.009709 | 2223.125 | 103.1794 | 9 |
| HP | 5 | 0.9595 | 0.957461 | 0.83898 | 0.023692 | 0.723645 | 0.106796 | 0.096326 | 0.009709 | 0.009709 | 2975.417 | 855.4714 | 80 |
| HT | 5 | 0.9615 | 0.953435 | 1.400288 | 0.029998 | 1.266308 | 0.116505 | 0.103941 | 0.009709 | 0.009709 | 2547.819 | 427.8728 | 63 |
| PT | 5 | 0.9611 | 0.944564 | 1.288854 | 0.030306 | 1.193854 | 0.165049 | 0.139159 | 0.009709 | 0.009709 | 2282.105 | 162.1592 | 15 |
| LQH | 5 | 0.957 | 0.95579 | 1.050994 | 0.024792 | 0.926461 | 0.106796 | 0.096326 | 0.009709 | 0.009709 | 2256.56 | 136.6138 | 14 |
| LQP | 5 | 0.9481 | 0.946035 | 1.100305 | 0.02888 | 0.929137 | 0.106796 | 0.096326 | 0.009709 | 0.009709 | 2283.828 | 163.8817 | 9 |
| LQT | 5 | 0.9592 | 0.953942 | 1.179176 | 0.027595 | 1.0458 | 0.126214 | 0.111365 | 0.009709 | 0.009709 | 2230.578 | 110.6323 | 9 |
| LHP | 5 | 0.9683 | 0.966538 | 0.549073 | 0.018279 | 0.483512 | 0.106796 | 0.096326 | 0.009709 | 0.009709 | 2230.093 | 110.147 | 24 |
| LHT | 5 | 0.9614 | 0.953375 | 1.401618 | 0.030042 | 1.267781 | 0.116505 | 0.103941 | 0.009709 | 0.009709 | 2576.912 | 456.9656 | 65 |
| LPT | 5 | 0.971 | 0.958666 | 1.115202 | 0.026963 | 1.033337 | 0.145631 | 0.125642 | 0.009709 | 0.009709 | 2191.257 | 71.31093 | 15 |
| QHP | 5 | 0.9529 | 0.951489 | 1.205859 | 0.027705 | 1.048209 | 0.116505 | 0.103941 | 0.009709 | 0.009709 | 2257.06 | 137.1141 | 16 |
| QHT | 5 | 0.9571 | 0.951811 | 1.309113 | 0.029073 | 1.156641 | 0.116505 | 0.103941 | 0.009709 | 0.009709 | 2233.037 | 113.0912 | 15 |
| QPT | 5 | 0.9563 | 0.950149 | 1.308662 | 0.029868 | 1.148132 | 0.126214 | 0.111365 | 0.009709 | 0.009709 | 2227.739 | 107.7926 | 12 |
| HPT | 5 | 0.9611 | 0.952869 | 1.327081 | 0.029212 | 1.198322 | 0.116505 | 0.103941 | 0.009709 | 0.009709 | 4619.723 | 2499.777 | 94 |
| LQHP | 5 | 0.9669 | 0.964189 | 0.820292 | 0.0203 | 0.743947 | 0.106796 | 0.096326 | 0.009709 | 0.009709 | 2194.287 | 74.34073 | 16 |
| LQHT | 5 | 0.959 | 0.954214 | 1.204692 | 0.02745 | 1.069647 | 0.126214 | 0.111365 | 0.009709 | 0.009709 | 2238.622 | 118.6759 | 14 |
| LQPT | 5 | 0.9687 | 0.961743 | 1.0032 | 0.02368 | 0.917859 | 0.135922 | 0.118599 | 0.009709 | 0.009709 | 2166.704 | 46.75784 | 11 |
| LHPT | 5 | 0.971 | 0.962282 | 0.907499 | 0.023414 | 0.835752 | 0.116505 | 0.103941 | 0.009709 | 0.009709 | 2189.99 | 70.04451 | 18 |
| QHPT | 5 | 0.9569 | 0.951138 | 1.295066 | 0.029357 | 1.140169 | 0.116505 | 0.103941 | 0.009709 | 0.009709 | 2235.323 | 115.3769 | 17 |
| LQHPT | 5 | 0.9694 | 0.963037 | 0.971335 | 0.022572 | 0.892207 | 0.126214 | 0.111365 | 0.009709 | 0.009709 | 2164.958 | 45.01232 | 12 |
| L | 5.5 | 0.8063 | 0.801082 | 1.855697 | 0.066596 | 0.670941 | 0.106796 | 0.096326 | 0.009709 | 0.009709 | 2560.027 | 440.0816 | 3 |
| Q | 5.5 | 0.8963 | 0.895232 | 2.271118 | 0.050855 | 1.621643 | 0.106796 | 0.096326 | 0.009709 | 0.009709 | 2436.818 | 316.8725 | 3 |
| H | 5.5 | 0.9608 | 0.95888 | 0.921759 | 0.024577 | 0.801781 | 0.106796 | 0.096326 | 0.009709 | 0.009709 | 3214.151 | 1094.205 | 84 |
| P | 5.5 | 0.7552 | 0.754567 | 6.112925 | 0.117749 | 2.243473 | 0.106796 | 0.096326 | 0.009709 | 0.009709 | 2553.166 | 433.2202 | 3 |
| T | 5.5 | 0.9555 | 0.924279 | 1.40646 | 0.04174 | 1.256774 | 0.145631 | 0.125642 | 0.009709 | 0.009709 | 2366.975 | 247.0296 | 12 |
| LQ | 5.5 | 0.9457 | 0.944553 | 1.163943 | 0.028385 | 0.989866 | 0.106796 | 0.096326 | 0.009709 | 0.009709 | 2313.156 | 193.2096 | 6 |
| LH | 5.5 | 0.9581 | 0.955096 | 0.968138 | 0.026331 | 0.828818 | 0.106796 | 0.096326 | 0.009709 | 0.009709 | 2571.649 | 451.7033 | 61 |
| LP | 5.5 | 0.8864 | 0.884293 | 1.283271 | 0.051806 | 0.549751 | 0.116505 | 0.103941 | 0.009709 | 0.009709 | 2445.331 | 325.3852 | 6 |
| LT | 5.5 | 0.9455 | 0.925552 | 1.108646 | 0.033712 | 0.986772 | 0.15534 | 0.132496 | 0.009709 | 0.009709 | 2363.59 | 243.6439 | 13 |
| QH | 5.5 | 0.9539 | 0.95272 | 1.17629 | 0.026551 | 1.027361 | 0.106796 | 0.096326 | 0.009709 | 0.009709 | 2268.34 | 148.394 | 16 |
| QP | 5.5 | 0.9103 | 0.907871 | 2.329271 | 0.049918 | 1.796464 | 0.106796 | 0.096326 | 0.009709 | 0.009709 | 2412.696 | 292.7498 | 6 |
| QT | 5.5 | 0.9564 | 0.950572 | 1.305866 | 0.029684 | 1.146741 | 0.116505 | 0.103941 | 0.009709 | 0.009709 | 2229.072 | 109.1263 | 9 |
| HP | 5.5 | 0.9586 | 0.956569 | 0.867926 | 0.024084 | 0.74958 | 0.106796 | 0.096326 | 0.009709 | 0.009709 | 2600.26 | 480.3141 | 65 |
| HT | 5.5 | 0.9611 | 0.952994 | 1.398273 | 0.03018 | 1.260196 | 0.116505 | 0.103941 | 0.009709 | 0.009709 | 2562.141 | 442.1956 | 63 |
| PT | 5.5 | 0.9502 | 0.93243 | 1.320518 | 0.036508 | 1.141139 | 0.145631 | 0.125642 | 0.009709 | 0.009709 | 2317.593 | 197.6469 | 13 |
| LQH | 5.5 | 0.9566 | 0.955361 | 1.058486 | 0.024961 | 0.931904 | 0.106796 | 0.096326 | 0.009709 | 0.009709 | 2264.68 | 144.7341 | 15 |
| LQP | 5.5 | 0.9472 | 0.945275 | 1.119639 | 0.029335 | 0.941357 | 0.106796 | 0.096326 | 0.009709 | 0.009709 | 2287.618 | 167.6718 | 9 |
| LQT | 5.5 | 0.959 | 0.953752 | 1.17591 | 0.027459 | 1.042666 | 0.116505 | 0.103941 | 0.009709 | 0.009709 | 2237.25 | 117.3038 | 9 |
| LHP | 5.5 | 0.9678 | 0.966045 | 0.559978 | 0.018393 | 0.493601 | 0.106796 | 0.096326 | 0.009709 | 0.009709 | 2234.436 | 114.4905 | 22 |
| LHT | 5.5 | 0.9599 | 0.951292 | 1.438258 | 0.031166 | 1.290668 | 0.106796 | 0.096326 | 0.009709 | 0.009709 | 2597.365 | 477.4187 | 65 |
| LPT | 5.5 | 0.9702 | 0.95833 | 1.122908 | 0.026894 | 1.040273 | 0.145631 | 0.125642 | 0.009709 | 0.009709 | 2200.633 | 80.68736 | 14 |
| QHP | 5.5 | 0.9521 | 0.95069 | 1.219511 | 0.028062 | 1.057672 | 0.106796 | 0.096326 | 0.009709 | 0.009709 | 2257.796 | 137.8503 | 14 |
| QHT | 5.5 | 0.9567 | 0.951332 | 1.315522 | 0.02925 | 1.160344 | 0.116505 | 0.103941 | 0.009709 | 0.009709 | 2238.895 | 118.949 | 15 |
| QPT | 5.5 | 0.9556 | 0.949621 | 1.325584 | 0.030037 | 1.160429 | 0.116505 | 0.103941 | 0.009709 | 0.009709 | 2231.584 | 111.638 | 11 |
| HPT | 5.5 | 0.9609 | 0.952775 | 1.326666 | 0.028962 | 1.198506 | 0.116505 | 0.103941 | 0.009709 | 0.009709 | 2811.715 | 691.7693 | 76 |
| LQHP | 5.5 | 0.9649 | 0.96214 | 0.888254 | 0.021383 | 0.80161 | 0.106796 | 0.096326 | 0.009709 | 0.009709 | 2213.378 | 93.43214 | 18 |
| LQHT | 5.5 | 0.9586 | 0.953971 | 1.202185 | 0.027355 | 1.066936 | 0.106796 | 0.096326 | 0.009709 | 0.009709 | 2235.475 | 115.5295 | 11 |
| LQPT | 5.5 | 0.9671 | 0.960919 | 1.019727 | 0.023902 | 0.929349 | 0.126214 | 0.111365 | 0.009709 | 0.009709 | 2174.086 | 54.14032 | 10 |
| LHPT | 5.5 | 0.9704 | 0.9622 | 0.907683 | 0.023259 | 0.834994 | 0.116505 | 0.103941 | 0.009709 | 0.009709 | 2197.419 | 77.4732 | 17 |
| QHPT | 5.5 | 0.9562 | 0.950592 | 1.310593 | 0.029601 | 1.150476 | 0.106796 | 0.096326 | 0.009709 | 0.009709 | 2242.022 | 122.0758 | 17 |
| LQHPT | 5.5 | 0.9672 | 0.961597 | 1.012493 | 0.023395 | 0.924659 | 0.126214 | 0.111365 | 0.009709 | 0.009709 | 2178.804 | 58.85839 | 12 |
| L | 6 | 0.8062 | 0.801056 | 1.889304 | 0.067228 | 0.683147 | 0.116505 | 0.103941 | 0.009709 | 0.009709 | 2560.106 | 440.1596 | 3 |
| Q | 6 | 0.8963 | 0.895212 | 2.270189 | 0.050843 | 1.620714 | 0.106796 | 0.096326 | 0.009709 | 0.009709 | 2436.907 | 316.9615 | 3 |
| H | 6 | 0.9605 | 0.958295 | 0.932866 | 0.024954 | 0.808874 | 0.106796 | 0.096326 | 0.009709 | 0.009709 | 2740.814 | 620.8682 | 71 |
| P | 6 | 0.7552 | 0.754637 | 6.107628 | 0.117694 | 2.24186 | 0.106796 | 0.096326 | 0.009709 | 0.009709 | 2553.192 | 433.2466 | 3 |
| T | 6 | 0.9387 | 0.922946 | 1.373079 | 0.040441 | 1.131353 | 0.135922 | 0.118599 | 0.009709 | 0.009709 | 2380.639 | 260.6934 | 10 |
| LQ | 6 | 0.9456 | 0.944474 | 1.166952 | 0.028465 | 0.992072 | 0.106796 | 0.096326 | 0.009709 | 0.009709 | 2313.64 | 193.6943 | 6 |
| LH | 6 | 0.9547 | 0.950594 | 1.035596 | 0.028374 | 0.875464 | 0.106796 | 0.096326 | 0.009709 | 0.009709 | 2636.927 | 516.9816 | 64 |
| LP | 6 | 0.886 | 0.883789 | 1.298737 | 0.052229 | 0.553631 | 0.116505 | 0.103941 | 0.009709 | 0.009709 | 2445.716 | 325.7702 | 6 |
| LT | 6 | 0.9237 | 0.908135 | 1.136394 | 0.038439 | 0.899831 | 0.135922 | 0.118599 | 0.009709 | 0.009709 | 2396.671 | 276.7248 | 11 |
| QH | 6 | 0.9533 | 0.952113 | 1.192557 | 0.026817 | 1.039149 | 0.106796 | 0.096326 | 0.009709 | 0.009709 | 2268.217 | 148.2713 | 14 |
| QP | 6 | 0.9103 | 0.907784 | 2.326364 | 0.049867 | 1.795014 | 0.106796 | 0.096326 | 0.009709 | 0.009709 | 2412.845 | 292.8988 | 6 |
| QT | 6 | 0.9558 | 0.950013 | 1.311568 | 0.029848 | 1.149061 | 0.116505 | 0.103941 | 0.009709 | 0.009709 | 2235.41 | 115.464 | 9 |
| HP | 6 | 0.9578 | 0.955232 | 0.891588 | 0.024539 | 0.770995 | 0.106796 | 0.096326 | 0.009709 | 0.009709 | 2597.31 | 477.3643 | 64 |
| HT | 6 | 0.9605 | 0.952063 | 1.433798 | 0.030735 | 1.290792 | 0.106796 | 0.096326 | 0.009709 | 0.009709 | 2675.549 | 555.6034 | 69 |
| PT | 6 | 0.9401 | 0.927327 | 1.317094 | 0.038505 | 1.066055 | 0.145631 | 0.125642 | 0.009709 | 0.009709 | 2338.402 | 218.4557 | 10 |
| LQH | 6 | 0.9562 | 0.955016 | 1.060679 | 0.025048 | 0.932576 | 0.106796 | 0.096326 | 0.009709 | 0.009709 | 2264.653 | 144.7068 | 13 |
| LQP | 6 | 0.9463 | 0.944579 | 1.14212 | 0.029773 | 0.95796 | 0.106796 | 0.096326 | 0.009709 | 0.009709 | 2288.627 | 168.6813 | 8 |
| LQT | 6 | 0.9587 | 0.953579 | 1.171423 | 0.027273 | 1.03792 | 0.126214 | 0.111365 | 0.009709 | 0.009709 | 2244.477 | 124.5316 | 9 |
| LHP | 6 | 0.9672 | 0.965365 | 0.573474 | 0.018557 | 0.506437 | 0.106796 | 0.096326 | 0.009709 | 0.009709 | 2252.56 | 132.614 | 24 |
| LHT | 6 | 0.9574 | 0.948722 | 1.453072 | 0.032205 | 1.287962 | 0.106796 | 0.096326 | 0.009709 | 0.009709 | 2530.839 | 410.893 | 58 |
| LPT | 6 | 0.9699 | 0.958034 | 1.118606 | 0.026783 | 1.036036 | 0.145631 | 0.125642 | 0.009709 | 0.009709 | 2207.68 | 87.73375 | 13 |
| QHP | 6 | 0.9514 | 0.949837 | 1.233967 | 0.028443 | 1.067199 | 0.106796 | 0.096326 | 0.009709 | 0.009709 | 2269.753 | 149.8067 | 16 |
| QHT | 6 | 0.9562 | 0.950779 | 1.317197 | 0.029455 | 1.159208 | 0.116505 | 0.103941 | 0.009709 | 0.009709 | 2247.497 | 127.551 | 16 |
| QPT | 6 | 0.9548 | 0.948948 | 1.3419 | 0.030307 | 1.172418 | 0.116505 | 0.103941 | 0.009709 | 0.009709 | 2238.835 | 118.8893 | 11 |
| HPT | 6 | 0.9604 | 0.952504 | 1.328172 | 0.028717 | 1.200346 | 0.116505 | 0.103941 | 0.009709 | 0.009709 | 2632.492 | 512.5457 | 68 |
| LQHP | 6 | 0.9628 | 0.960083 | 0.918628 | 0.022384 | 0.823416 | 0.106796 | 0.096326 | 0.009709 | 0.009709 | 2227.115 | 107.1688 | 18 |
| LQHT | 6 | 0.9581 | 0.95367 | 1.195502 | 0.027248 | 1.059769 | 0.106796 | 0.096326 | 0.009709 | 0.009709 | 2245.675 | 125.7293 | 13 |
| LQPT | 6 | 0.9658 | 0.95991 | 1.036059 | 0.024268 | 0.940198 | 0.126214 | 0.111365 | 0.009709 | 0.009709 | 2183.359 | 63.41346 | 10 |
| LHPT | 6 | 0.9698 | 0.961887 | 0.913795 | 0.023301 | 0.840003 | 0.116505 | 0.103941 | 0.009709 | 0.009709 | 2216.429 | 96.48331 | 20 |
| QHPT | 6 | 0.9553 | 0.949921 | 1.325452 | 0.029844 | 1.16048 | 0.106796 | 0.096326 | 0.009709 | 0.009709 | 2243.097 | 123.1511 | 15 |
| LQHPT | 6 | 0.9661 | 0.960444 | 1.033951 | 0.023939 | 0.940381 | 0.126214 | 0.111365 | 0.009709 | 0.009709 | 2184.371 | 64.42517 | 11 |
| L | 6.5 | 0.8061 | 0.800944 | 1.920391 | 0.067828 | 0.694896 | 0.106796 | 0.096326 | 0.009709 | 0.009709 | 2560.191 | 440.2456 | 3 |
| Q | 6.5 | 0.8962 | 0.89521 | 2.268976 | 0.050819 | 1.619514 | 0.106796 | 0.096326 | 0.009709 | 0.009709 | 2437.003 | 317.0575 | 3 |
| H | 6.5 | 0.9599 | 0.957495 | 0.939641 | 0.025459 | 0.810549 | 0.106796 | 0.096326 | 0.009709 | 0.009709 | 2500.159 | 380.2134 | 52 |
| P | 6.5 | 0.7553 | 0.754704 | 6.100846 | 0.117635 | 2.239545 | 0.106796 | 0.096326 | 0.009709 | 0.009709 | 2553.223 | 433.2768 | 3 |
| T | 6.5 | 0.9393 | 0.925428 | 1.118788 | 0.03759 | 0.887787 | 0.145631 | 0.125642 | 0.009709 | 0.009709 | 2396.781 | 276.8349 | 10 |
| LQ | 6.5 | 0.9456 | 0.944396 | 1.169387 | 0.028532 | 0.993874 | 0.106796 | 0.096326 | 0.009709 | 0.009709 | 2314.16 | 194.2142 | 6 |
| LH | 6.5 | 0.9504 | 0.946698 | 1.056454 | 0.029505 | 0.883261 | 0.116505 | 0.103941 | 0.009709 | 0.009709 | 2497.978 | 378.0321 | 48 |
| LP | 6.5 | 0.8855 | 0.883286 | 1.314177 | 0.052628 | 0.557235 | 0.116505 | 0.103941 | 0.009709 | 0.009709 | 2446.139 | 326.1934 | 6 |
| LT | 6.5 | 0.9213 | 0.90621 | 1.134849 | 0.040007 | 0.869196 | 0.135922 | 0.118599 | 0.009709 | 0.009709 | 2404.145 | 284.1994 | 11 |
| QH | 6.5 | 0.9527 | 0.95135 | 1.210369 | 0.027204 | 1.05187 | 0.106796 | 0.096326 | 0.009709 | 0.009709 | 2268.181 | 148.2352 | 12 |
| QP | 6.5 | 0.9102 | 0.907715 | 2.321802 | 0.049771 | 1.792221 | 0.106796 | 0.096326 | 0.009709 | 0.009709 | 2413.018 | 293.0723 | 6 |
| QT | 6.5 | 0.9552 | 0.949336 | 1.317933 | 0.030045 | 1.151752 | 0.116505 | 0.103941 | 0.009709 | 0.009709 | 2239.196 | 119.2501 | 8 |
| HP | 6.5 | 0.9564 | 0.953559 | 0.916213 | 0.025019 | 0.793207 | 0.106796 | 0.096326 | 0.009709 | 0.009709 | 2389.765 | 269.8191 | 39 |
| HT | 6.5 | 0.96 | 0.95157 | 1.433333 | 0.030895 | 1.285595 | 0.106796 | 0.096326 | 0.009709 | 0.009709 | 2389.36 | 269.4139 | 39 |
| PT | 6.5 | 0.9388 | 0.92589 | 1.308194 | 0.039267 | 1.042765 | 0.145631 | 0.125642 | 0.009709 | 0.009709 | 2345.166 | 225.2203 | 10 |
| LQH | 6.5 | 0.9559 | 0.954607 | 1.0662 | 0.025145 | 0.936341 | 0.106796 | 0.096326 | 0.009709 | 0.009709 | 2267.34 | 147.3942 | 12 |
| LQP | 6.5 | 0.9458 | 0.944002 | 1.150768 | 0.030067 | 0.96118 | 0.106796 | 0.096326 | 0.009709 | 0.009709 | 2291.35 | 171.4041 | 8 |
| LQT | 6.5 | 0.9582 | 0.953237 | 1.16766 | 0.027089 | 1.03343 | 0.126214 | 0.111365 | 0.009709 | 0.009709 | 2248.81 | 128.8642 | 8 |
| LHP | 6.5 | 0.9665 | 0.964458 | 0.581146 | 0.018732 | 0.513718 | 0.106796 | 0.096326 | 0.009709 | 0.009709 | 2267.68 | 147.7343 | 25 |
| LHT | 6.5 | 0.9539 | 0.945121 | 1.43266 | 0.033641 | 1.241822 | 0.106796 | 0.096326 | 0.009709 | 0.009709 | 2490.094 | 370.1481 | 51 |
| LPT | 6.5 | 0.9694 | 0.957725 | 1.111358 | 0.02668 | 1.028758 | 0.135922 | 0.118599 | 0.009709 | 0.009709 | 2218.112 | 98.16639 | 13 |
| QHP | 6.5 | 0.9506 | 0.949126 | 1.250877 | 0.028786 | 1.078388 | 0.106796 | 0.096326 | 0.009709 | 0.009709 | 2273.402 | 153.4564 | 15 |
| QHT | 6.5 | 0.9557 | 0.950288 | 1.319129 | 0.029613 | 1.157601 | 0.116505 | 0.103941 | 0.009709 | 0.009709 | 2250.845 | 130.8988 | 15 |
| QPT | 6.5 | 0.954 | 0.948711 | 1.355545 | 0.030296 | 1.183291 | 0.106796 | 0.096326 | 0.009709 | 0.009709 | 2242.647 | 122.7009 | 10 |
| HPT | 6.5 | 0.9598 | 0.951662 | 1.33631 | 0.028795 | 1.20914 | 0.116505 | 0.103941 | 0.009709 | 0.009709 | 2980.804 | 860.858 | 80 |
| LQHP | 6.5 | 0.9607 | 0.957898 | 0.974195 | 0.023492 | 0.869232 | 0.106796 | 0.096326 | 0.009709 | 0.009709 | 2230.768 | 110.822 | 14 |
| LQHT | 6.5 | 0.9577 | 0.953324 | 1.190084 | 0.027148 | 1.053497 | 0.116505 | 0.103941 | 0.009709 | 0.009709 | 2248.436 | 128.4903 | 12 |
| LQPT | 6.5 | 0.9644 | 0.958784 | 1.056001 | 0.024784 | 0.953781 | 0.126214 | 0.111365 | 0.009709 | 0.009709 | 2193.497 | 73.5514 | 10 |
| LHPT | 6.5 | 0.9692 | 0.961405 | 0.92258 | 0.023477 | 0.847551 | 0.116505 | 0.103941 | 0.009709 | 0.009709 | 2227.093 | 107.1474 | 20 |
| QHPT | 6.5 | 0.9543 | 0.949141 | 1.343697 | 0.030157 | 1.174061 | 0.106796 | 0.096326 | 0.009709 | 0.009709 | 2244.273 | 124.3272 | 13 |
| LQHPT | 6.5 | 0.9648 | 0.959281 | 1.053594 | 0.024533 | 0.953479 | 0.126214 | 0.111365 | 0.009709 | 0.009709 | 2195.848 | 75.90197 | 12 |
| L | 7 | 0.8059 | 0.800839 | 1.952484 | 0.068435 | 0.706908 | 0.106796 | 0.096326 | 0.009709 | 0.009709 | 2560.285 | 440.3393 | 3 |
| Q | 7 | 0.8962 | 0.895175 | 2.267835 | 0.050809 | 1.618497 | 0.106796 | 0.096326 | 0.009709 | 0.009709 | 2437.106 | 317.1603 | 3 |
| H | 7 | 0.9592 | 0.956739 | 0.939204 | 0.025793 | 0.804024 | 0.106796 | 0.096326 | 0.009709 | 0.009709 | 2555.403 | 435.4573 | 56 |
| P | 7 | 0.7554 | 0.754763 | 6.093797 | 0.117579 | 2.236999 | 0.106796 | 0.096326 | 0.009709 | 0.009709 | 2553.258 | 433.3125 | 3 |
| T | 7 | 0.9185 | 0.901067 | 0.870056 | 0.033059 | 0.822143 | 0.126214 | 0.111365 | 0.009709 | 0.009709 | 2405.104 | 285.1581 | 8 |
| LQ | 7 | 0.9455 | 0.9443 | 1.171878 | 0.028589 | 0.995888 | 0.106796 | 0.096326 | 0.009709 | 0.009709 | 2314.704 | 194.758 | 6 |
| LH | 7 | 0.949 | 0.943232 | 1.037499 | 0.029741 | 0.87508 | 0.116505 | 0.103941 | 0.009709 | 0.009709 | 2513.112 | 393.1662 | 48 |
| LP | 7 | 0.885 | 0.882697 | 1.330793 | 0.053071 | 0.561239 | 0.106796 | 0.096326 | 0.009709 | 0.009709 | 2446.607 | 326.6609 | 6 |
| LT | 7 | 0.9188 | 0.904146 | 1.134018 | 0.041435 | 0.839201 | 0.135922 | 0.118599 | 0.009709 | 0.009709 | 2411.776 | 291.8298 | 11 |
| QH | 7 | 0.952 | 0.950571 | 1.224986 | 0.027582 | 1.061067 | 0.106796 | 0.096326 | 0.009709 | 0.009709 | 2273.707 | 153.7614 | 12 |
| QP | 7 | 0.9101 | 0.907608 | 2.320049 | 0.049718 | 1.791336 | 0.106796 | 0.096326 | 0.009709 | 0.009709 | 2413.205 | 293.2594 | 6 |
| QT | 7 | 0.9545 | 0.948673 | 1.325784 | 0.030331 | 1.154589 | 0.116505 | 0.103941 | 0.009709 | 0.009709 | 2245.547 | 125.6013 | 8 |
| HP | 7 | 0.9546 | 0.95148 | 0.916339 | 0.025116 | 0.793087 | 0.106796 | 0.096326 | 0.009709 | 0.009709 | 2425.699 | 305.7534 | 43 |
| HT | 7 | 0.9591 | 0.950457 | 1.43633 | 0.031506 | 1.278928 | 0.106796 | 0.096326 | 0.009709 | 0.009709 | 2424.639 | 304.6929 | 42 |
| PT | 7 | 0.937 | 0.923792 | 1.33082 | 0.04045 | 1.045585 | 0.145631 | 0.125642 | 0.009709 | 0.009709 | 2349.216 | 229.2701 | 9 |
| LQH | 7 | 0.9555 | 0.954247 | 1.068647 | 0.025201 | 0.937414 | 0.116505 | 0.103941 | 0.009709 | 0.009709 | 2278.276 | 158.3301 | 14 |
| LQP | 7 | 0.9452 | 0.943467 | 1.165794 | 0.030315 | 0.972682 | 0.106796 | 0.096326 | 0.009709 | 0.009709 | 2293.83 | 173.8843 | 8 |
| LQT | 7 | 0.9573 | 0.952466 | 1.159295 | 0.027044 | 1.022057 | 0.116505 | 0.103941 | 0.009709 | 0.009709 | 2259.521 | 139.5748 | 9 |
| LHP | 7 | 0.9657 | 0.963774 | 0.596613 | 0.018869 | 0.528026 | 0.106796 | 0.096326 | 0.009709 | 0.009709 | 2269.274 | 149.3284 | 22 |
| LHT | 7 | 0.9525 | 0.944034 | 1.419479 | 0.034098 | 1.224728 | 0.116505 | 0.103941 | 0.009709 | 0.009709 | 2423.084 | 303.1382 | 38 |
| LPT | 7 | 0.9689 | 0.957367 | 1.105592 | 0.026615 | 1.022649 | 0.145631 | 0.125642 | 0.009709 | 0.009709 | 2225.669 | 105.7234 | 12 |
| QHP | 7 | 0.9498 | 0.948297 | 1.266326 | 0.029114 | 1.089233 | 0.106796 | 0.096326 | 0.009709 | 0.009709 | 2279.485 | 159.5392 | 15 |
| QHT | 7 | 0.9549 | 0.949666 | 1.323982 | 0.029868 | 1.158183 | 0.106796 | 0.096326 | 0.009709 | 0.009709 | 2253.843 | 133.8971 | 14 |
| QPT | 7 | 0.9536 | 0.948456 | 1.371118 | 0.030435 | 1.196158 | 0.106796 | 0.096326 | 0.009709 | 0.009709 | 2247.756 | 127.8097 | 10 |
| HPT | 7 | 0.9591 | 0.950373 | 1.339485 | 0.028992 | 1.213243 | 0.116505 | 0.103941 | 0.009709 | 0.009709 | 2493.77 | 373.8237 | 56 |
| LQHP | 7 | 0.9588 | 0.956142 | 1.022458 | 0.024381 | 0.907464 | 0.106796 | 0.096326 | 0.009709 | 0.009709 | 2241.824 | 121.8779 | 14 |
| LQHT | 7 | 0.9572 | 0.95284 | 1.191883 | 0.027156 | 1.052749 | 0.116505 | 0.103941 | 0.009709 | 0.009709 | 2253.905 | 133.959 | 12 |
| LQPT | 7 | 0.9627 | 0.957619 | 1.072336 | 0.025256 | 0.963145 | 0.126214 | 0.111365 | 0.009709 | 0.009709 | 2204.264 | 84.3178 | 10 |
| LHPT | 7 | 0.9686 | 0.961104 | 0.918936 | 0.023505 | 0.843042 | 0.116505 | 0.103941 | 0.009709 | 0.009709 | 2231.136 | 111.1898 | 18 |
| QHPT | 7 | 0.9533 | 0.948666 | 1.351496 | 0.030261 | 1.178789 | 0.106796 | 0.096326 | 0.009709 | 0.009709 | 2245.915 | 125.9694 | 11 |
| LQHPT | 7 | 0.9632 | 0.957964 | 1.070869 | 0.025107 | 0.963447 | 0.126214 | 0.111365 | 0.009709 | 0.009709 | 2208.691 | 88.74482 | 13 |
| L | 7.5 | 0.8058 | 0.800704 | 1.986149 | 0.069048 | 0.719329 | 0.106796 | 0.096326 | 0.009709 | 0.009709 | 2560.387 | 440.4408 | 3 |
| Q | 7.5 | 0.8962 | 0.895159 | 2.26599 | 0.050775 | 1.616912 | 0.106796 | 0.096326 | 0.009709 | 0.009709 | 2437.216 | 317.27 | 3 |
| H | 7.5 | 0.9582 | 0.955329 | 0.943453 | 0.026534 | 0.799038 | 0.106796 | 0.096326 | 0.009709 | 0.009709 | 2441.191 | 321.2448 | 37 |
| P | 7.5 | 0.7555 | 0.754833 | 6.0863 | 0.117517 | 2.233896 | 0.106796 | 0.096326 | 0.009709 | 0.009709 | 2553.297 | 433.3509 | 3 |
| T | 7.5 | 0.9124 | 0.900778 | 0.832827 | 0.023881 | 0.791847 | 0.116505 | 0.103941 | 0.009709 | 0.009709 | 2406.127 | 286.1815 | 6 |
| LQ | 7.5 | 0.9455 | 0.944228 | 1.173934 | 0.028645 | 0.997394 | 0.106796 | 0.096326 | 0.009709 | 0.009709 | 2315.259 | 195.3132 | 6 |
| LH | 7.5 | 0.9448 | 0.938868 | 1.001186 | 0.029287 | 0.845548 | 0.116505 | 0.103941 | 0.009709 | 0.009709 | 2551.312 | 431.3659 | 51 |
| LP | 7.5 | 0.8844 | 0.882138 | 1.346178 | 0.053458 | 0.564902 | 0.106796 | 0.096326 | 0.009709 | 0.009709 | 2447.115 | 327.169 | 6 |
| LT | 7.5 | 0.9163 | 0.902327 | 1.126464 | 0.042464 | 0.808073 | 0.135922 | 0.118599 | 0.009709 | 0.009709 | 2416.774 | 296.8286 | 10 |
| QH | 7.5 | 0.9511 | 0.949655 | 1.240309 | 0.028069 | 1.069827 | 0.106796 | 0.096326 | 0.009709 | 0.009709 | 2284.99 | 165.0444 | 14 |
| QP | 7.5 | 0.91 | 0.907499 | 2.317836 | 0.049658 | 1.790201 | 0.106796 | 0.096326 | 0.009709 | 0.009709 | 2413.397 | 293.451 | 6 |
| QT | 7.5 | 0.9536 | 0.947848 | 1.333805 | 0.03068 | 1.156414 | 0.116505 | 0.103941 | 0.009709 | 0.009709 | 2252.289 | 132.3431 | 8 |
| HP | 7.5 | 0.9523 | 0.948814 | 0.916039 | 0.025205 | 0.790321 | 0.106796 | 0.096326 | 0.009709 | 0.009709 | 2396.515 | 276.5693 | 35 |
| HT | 7.5 | 0.9574 | 0.948628 | 1.423655 | 0.03224 | 1.252688 | 0.106796 | 0.096326 | 0.009709 | 0.009709 | 2403.471 | 283.5253 | 34 |
| PT | 7.5 | 0.9346 | 0.921059 | 1.357743 | 0.041955 | 1.047989 | 0.145631 | 0.125642 | 0.009709 | 0.009709 | 2358.341 | 238.3954 | 10 |
| LQH | 7.5 | 0.9539 | 0.952228 | 1.09072 | 0.026014 | 0.949956 | 0.116505 | 0.103941 | 0.009709 | 0.009709 | 2291.896 | 171.9501 | 15 |
| LQP | 7.5 | 0.9448 | 0.942961 | 1.180586 | 0.030575 | 0.983328 | 0.106796 | 0.096326 | 0.009709 | 0.009709 | 2296.049 | 176.1028 | 8 |
| LQT | 7.5 | 0.9556 | 0.95153 | 1.148513 | 0.02698 | 1.006221 | 0.106796 | 0.096326 | 0.009709 | 0.009709 | 2264.887 | 144.9412 | 8 |
| LHP | 7.5 | 0.9649 | 0.962568 | 0.609651 | 0.019282 | 0.539267 | 0.106796 | 0.096326 | 0.009709 | 0.009709 | 2270.538 | 150.5925 | 19 |
| LHT | 7.5 | 0.9523 | 0.942749 | 1.4162 | 0.034516 | 1.220903 | 0.116505 | 0.103941 | 0.009709 | 0.009709 | 2466.156 | 346.2099 | 43 |
| LPT | 7.5 | 0.9684 | 0.956809 | 1.101303 | 0.026601 | 1.018258 | 0.126214 | 0.111365 | 0.009709 | 0.009709 | 2235.912 | 115.9658 | 12 |
| QHP | 7.5 | 0.9491 | 0.947884 | 1.27604 | 0.029232 | 1.095493 | 0.106796 | 0.096326 | 0.009709 | 0.009709 | 2279.87 | 159.9245 | 13 |
| QHT | 7.5 | 0.9542 | 0.949045 | 1.32445 | 0.03007 | 1.153777 | 0.106796 | 0.096326 | 0.009709 | 0.009709 | 2265.262 | 145.3161 | 16 |
| QPT | 7.5 | 0.9532 | 0.948225 | 1.384057 | 0.030597 | 1.206593 | 0.106796 | 0.096326 | 0.009709 | 0.009709 | 2249.967 | 130.0214 | 9 |
| HPT | 7.5 | 0.958 | 0.948745 | 1.337111 | 0.029207 | 1.211212 | 0.116505 | 0.103941 | 0.009709 | 0.009709 | 2479.053 | 359.107 | 53 |
| LQHP | 7.5 | 0.9571 | 0.954378 | 1.059814 | 0.025303 | 0.935184 | 0.116505 | 0.103941 | 0.009709 | 0.009709 | 2249.808 | 129.8619 | 13 |
| LQHT | 7.5 | 0.9558 | 0.951875 | 1.17931 | 0.0272 | 1.036164 | 0.116505 | 0.103941 | 0.009709 | 0.009709 | 2258.59 | 138.6442 | 10 |
| LQPT | 7.5 | 0.9612 | 0.956593 | 1.081513 | 0.025602 | 0.966586 | 0.116505 | 0.103941 | 0.009709 | 0.009709 | 2210.775 | 90.82865 | 9 |
| LHPT | 7.5 | 0.9683 | 0.960699 | 0.926271 | 0.023624 | 0.849635 | 0.116505 | 0.103941 | 0.009709 | 0.009709 | 2231.031 | 111.0849 | 15 |
| QHPT | 7.5 | 0.953 | 0.948466 | 1.361262 | 0.030335 | 1.186334 | 0.106796 | 0.096326 | 0.009709 | 0.009709 | 2257.488 | 137.5417 | 14 |
| LQHPT | 7.5 | 0.9614 | 0.956689 | 1.083565 | 0.02557 | 0.969567 | 0.116505 | 0.103941 | 0.009709 | 0.009709 | 2212.114 | 92.16805 | 10 |
| L | 8 | 0.8055 | 0.800524 | 2.021658 | 0.06969 | 0.732251 | 0.116505 | 0.103941 | 0.009709 | 0.009709 | 2560.496 | 440.5499 | 3 |
| Q | 8 | 0.8962 | 0.895136 | 2.264758 | 0.050764 | 1.615471 | 0.106796 | 0.096326 | 0.009709 | 0.009709 | 2437.332 | 317.3864 | 3 |
| H | 8 | 0.9565 | 0.952995 | 0.934122 | 0.02773 | 0.773509 | 0.106796 | 0.096326 | 0.009709 | 0.009709 | 2447.016 | 327.07 | 34 |
| P | 8 | 0.7556 | 0.754928 | 6.077891 | 0.117428 | 2.23012 | 0.106796 | 0.096326 | 0.009709 | 0.009709 | 2553.338 | 433.3919 | 3 |
| T | 8 | 0.9124 | 0.901445 | 0.831498 | 0.023047 | 0.78059 | 0.106796 | 0.096326 | 0.009709 | 0.009709 | 2411.08 | 291.1338 | 6 |
| LQ | 8 | 0.9454 | 0.944184 | 1.175052 | 0.028677 | 0.998047 | 0.106796 | 0.096326 | 0.009709 | 0.009709 | 2315.841 | 195.8953 | 6 |
| LH | 8 | 0.939 | 0.932701 | 0.975207 | 0.029163 | 0.814905 | 0.116505 | 0.103941 | 0.009709 | 0.009709 | 2499.533 | 379.5875 | 41 |
| LP | 8 | 0.8838 | 0.881658 | 1.362063 | 0.05381 | 0.5686 | 0.106796 | 0.096326 | 0.009709 | 0.009709 | 2447.634 | 327.6876 | 6 |
| LT | 8 | 0.914 | 0.9002 | 1.120989 | 0.043397 | 0.784296 | 0.135922 | 0.118599 | 0.009709 | 0.009709 | 2423.903 | 303.9568 | 10 |
| QH | 8 | 0.9503 | 0.948804 | 1.256148 | 0.028544 | 1.078476 | 0.106796 | 0.096326 | 0.009709 | 0.009709 | 2287.989 | 168.0433 | 13 |
| QP | 8 | 0.9099 | 0.907397 | 2.31575 | 0.0496 | 1.788841 | 0.106796 | 0.096326 | 0.009709 | 0.009709 | 2413.61 | 293.664 | 6 |
| QT | 8 | 0.9526 | 0.946971 | 1.335817 | 0.030978 | 1.151759 | 0.116505 | 0.103941 | 0.009709 | 0.009709 | 2259.386 | 139.4405 | 8 |
| HP | 8 | 0.9492 | 0.945999 | 0.913199 | 0.025301 | 0.778345 | 0.106796 | 0.096326 | 0.009709 | 0.009709 | 2377.046 | 257.1005 | 27 |
| HT | 8 | 0.9545 | 0.944914 | 1.432431 | 0.034435 | 1.230688 | 0.106796 | 0.096326 | 0.009709 | 0.009709 | 2387.423 | 267.4775 | 25 |
| PT | 8 | 0.9316 | 0.91815 | 1.388258 | 0.043497 | 1.050997 | 0.145631 | 0.125642 | 0.009709 | 0.009709 | 2365.811 | 245.8653 | 10 |
| LQH | 8 | 0.951 | 0.948398 | 1.120087 | 0.027166 | 0.961223 | 0.106796 | 0.096326 | 0.009709 | 0.009709 | 2305.055 | 185.1091 | 14 |
| LQP | 8 | 0.9443 | 0.942526 | 1.190387 | 0.030758 | 0.990171 | 0.106796 | 0.096326 | 0.009709 | 0.009709 | 2298.137 | 178.1916 | 8 |
| LQT | 8 | 0.9542 | 0.950295 | 1.141375 | 0.027175 | 0.99304 | 0.106796 | 0.096326 | 0.009709 | 0.009709 | 2271.867 | 151.9215 | 8 |
| LHP | 8 | 0.9636 | 0.961438 | 0.618171 | 0.019571 | 0.545032 | 0.106796 | 0.096326 | 0.009709 | 0.009709 | 2288.74 | 168.7942 | 21 |
| LHT | 8 | 0.9505 | 0.939458 | 1.390894 | 0.03514 | 1.199129 | 0.116505 | 0.103941 | 0.009709 | 0.009709 | 2501.21 | 381.2643 | 46 |
| LPT | 8 | 0.9675 | 0.955941 | 1.097244 | 0.026748 | 1.013236 | 0.126214 | 0.111365 | 0.009709 | 0.009709 | 2246.675 | 126.7293 | 12 |
| QHP | 8 | 0.9488 | 0.947567 | 1.288006 | 0.029449 | 1.104595 | 0.106796 | 0.096326 | 0.009709 | 0.009709 | 2286.107 | 166.161 | 14 |
| QHT | 8 | 0.9534 | 0.948399 | 1.324885 | 0.030294 | 1.148533 | 0.106796 | 0.096326 | 0.009709 | 0.009709 | 2265.422 | 145.476 | 14 |
| QPT | 8 | 0.953 | 0.947957 | 1.395658 | 0.030768 | 1.215601 | 0.106796 | 0.096326 | 0.009709 | 0.009709 | 2254.676 | 134.7306 | 9 |
| HPT | 8 | 0.9562 | 0.946606 | 1.327814 | 0.029308 | 1.201692 | 0.106796 | 0.096326 | 0.009709 | 0.009709 | 2397.028 | 277.0825 | 39 |
| LQHP | 8 | 0.9553 | 0.952733 | 1.081563 | 0.026083 | 0.947402 | 0.116505 | 0.103941 | 0.009709 | 0.009709 | 2268.59 | 148.6437 | 16 |
| LQHT | 8 | 0.9547 | 0.950887 | 1.166802 | 0.027379 | 1.018232 | 0.116505 | 0.103941 | 0.009709 | 0.009709 | 2268.762 | 148.8162 | 11 |
| LQPT | 8 | 0.96 | 0.955639 | 1.092634 | 0.025906 | 0.972254 | 0.116505 | 0.103941 | 0.009709 | 0.009709 | 2218.233 | 98.28745 | 9 |
| LHPT | 8 | 0.9679 | 0.96011 | 0.93446 | 0.023828 | 0.857103 | 0.126214 | 0.111365 | 0.009709 | 0.009709 | 2245.432 | 125.4861 | 17 |
| QHPT | 8 | 0.9526 | 0.948183 | 1.370286 | 0.03048 | 1.1931 | 0.106796 | 0.096326 | 0.009709 | 0.009709 | 2261.332 | 141.3865 | 14 |
| LQHPT | 8 | 0.9603 | 0.955855 | 1.088528 | 0.02581 | 0.969744 | 0.116505 | 0.103941 | 0.009709 | 0.009709 | 2217.113 | 97.16678 | 9 |
| L | 8.5 | 0.8053 | 0.800278 | 2.057402 | 0.070338 | 0.745662 | 0.116505 | 0.103941 | 0.009709 | 0.009709 | 2560.612 | 440.6666 | 3 |
| Q | 8.5 | 0.8962 | 0.895095 | 2.264012 | 0.050756 | 1.614752 | 0.106796 | 0.096326 | 0.009709 | 0.009709 | 2437.455 | 317.5094 | 3 |
| H | 8.5 | 0.952 | 0.947293 | 0.936899 | 0.030695 | 0.744078 | 0.106796 | 0.096326 | 0.009709 | 0.009709 | 2443.916 | 323.9705 | 28 |
| P | 8.5 | 0.7556 | 0.755021 | 6.069273 | 0.117345 | 2.226391 | 0.106796 | 0.096326 | 0.009709 | 0.009709 | 2553.381 | 433.4355 | 3 |
| T | 8.5 | 0.9124 | 0.902135 | 0.829859 | 0.022465 | 0.779733 | 0.097087 | 0.088521 | 0.009709 | 0.009709 | 2416.236 | 296.29 | 6 |
| LQ | 8.5 | 0.9453 | 0.944113 | 1.176347 | 0.028718 | 0.998838 | 0.106796 | 0.096326 | 0.009709 | 0.009709 | 2316.459 | 196.5129 | 6 |
| LH | 8.5 | 0.9318 | 0.924365 | 0.951367 | 0.029904 | 0.776491 | 0.116505 | 0.103941 | 0.009709 | 0.009709 | 2516.888 | 396.9418 | 41 |
| LP | 8.5 | 0.8833 | 0.88115 | 1.378171 | 0.054182 | 0.572809 | 0.106796 | 0.096326 | 0.009709 | 0.009709 | 2445.784 | 325.8383 | 5 |
| LT | 8.5 | 0.9115 | 0.898184 | 1.110747 | 0.044035 | 0.759671 | 0.135922 | 0.118599 | 0.009709 | 0.009709 | 2428.648 | 308.7018 | 9 |
| QH | 8.5 | 0.9494 | 0.947834 | 1.271981 | 0.029076 | 1.086307 | 0.106796 | 0.096326 | 0.009709 | 0.009709 | 2299.565 | 179.6193 | 15 |
| QP | 8.5 | 0.9098 | 0.907294 | 2.313759 | 0.049545 | 1.787718 | 0.106796 | 0.096326 | 0.009709 | 0.009709 | 2413.832 | 293.8865 | 6 |
| QT | 8.5 | 0.9515 | 0.946201 | 1.338167 | 0.031205 | 1.146363 | 0.126214 | 0.111365 | 0.009709 | 0.009709 | 2262.992 | 143.0456 | 7 |
| HP | 8.5 | 0.9461 | 0.942584 | 0.926929 | 0.026059 | 0.775792 | 0.106796 | 0.096326 | 0.009709 | 0.009709 | 2380.532 | 260.5862 | 24 |
| HT | 8.5 | 0.949 | 0.93946 | 1.431498 | 0.036443 | 1.197506 | 0.106796 | 0.096326 | 0.009709 | 0.009709 | 2377.329 | 257.3836 | 15 |
| PT | 8.5 | 0.9284 | 0.915195 | 1.42483 | 0.045076 | 1.057342 | 0.145631 | 0.125642 | 0.009709 | 0.009709 | 2373.393 | 253.4469 | 10 |
| LQH | 8.5 | 0.9496 | 0.9477 | 1.125125 | 0.027408 | 0.963974 | 0.106796 | 0.096326 | 0.009709 | 0.009709 | 2307.801 | 187.8549 | 12 |
| LQP | 8.5 | 0.9439 | 0.942056 | 1.207579 | 0.031019 | 1.003891 | 0.106796 | 0.096326 | 0.009709 | 0.009709 | 2299.957 | 180.0111 | 8 |
| LQT | 8.5 | 0.9527 | 0.949783 | 1.138548 | 0.027141 | 0.986645 | 0.106796 | 0.096326 | 0.009709 | 0.009709 | 2275.578 | 155.6318 | 7 |
| LHP | 8.5 | 0.9624 | 0.9599 | 0.638249 | 0.020023 | 0.562125 | 0.106796 | 0.096326 | 0.009709 | 0.009709 | 2289.529 | 169.5835 | 18 |
| LHT | 8.5 | 0.9471 | 0.932874 | 1.311194 | 0.035707 | 1.133381 | 0.116505 | 0.103941 | 0.009709 | 0.009709 | 2471.301 | 351.3549 | 38 |
| LPT | 8.5 | 0.9666 | 0.955 | 1.093429 | 0.026886 | 1.008131 | 0.126214 | 0.111365 | 0.009709 | 0.009709 | 2254.635 | 134.6889 | 11 |
| QHP | 8.5 | 0.9485 | 0.947231 | 1.302353 | 0.029696 | 1.115906 | 0.106796 | 0.096326 | 0.009709 | 0.009709 | 2286.823 | 166.8774 | 13 |
| QHT | 8.5 | 0.9525 | 0.947629 | 1.327452 | 0.030602 | 1.144612 | 0.106796 | 0.096326 | 0.009709 | 0.009709 | 2268.662 | 148.7162 | 13 |
| QPT | 8.5 | 0.9527 | 0.947651 | 1.410952 | 0.030983 | 1.228184 | 0.106796 | 0.096326 | 0.009709 | 0.009709 | 2259.445 | 139.4992 | 9 |
| HPT | 8.5 | 0.9536 | 0.943719 | 1.317175 | 0.02949 | 1.186732 | 0.116505 | 0.103941 | 0.009709 | 0.009709 | 2395.83 | 275.8846 | 36 |
| LQHP | 8.5 | 0.9536 | 0.95112 | 1.103648 | 0.026791 | 0.959988 | 0.116505 | 0.103941 | 0.009709 | 0.009709 | 2269.841 | 149.8947 | 13 |
| LQHT | 8.5 | 0.9532 | 0.949578 | 1.158016 | 0.027686 | 1.002763 | 0.116505 | 0.103941 | 0.009709 | 0.009709 | 2277.155 | 157.2093 | 11 |
| LQPT | 8.5 | 0.9588 | 0.954643 | 1.103749 | 0.026197 | 0.978222 | 0.116505 | 0.103941 | 0.009709 | 0.009709 | 2225.64 | 105.6937 | 9 |
| LHPT | 8.5 | 0.9675 | 0.959514 | 0.943518 | 0.024045 | 0.865124 | 0.126214 | 0.111365 | 0.009709 | 0.009709 | 2256.713 | 136.7674 | 18 |
| QHPT | 8.5 | 0.9522 | 0.947849 | 1.381152 | 0.030652 | 1.201638 | 0.106796 | 0.096326 | 0.009709 | 0.009709 | 2265.205 | 145.2587 | 14 |
| LQHPT | 8.5 | 0.9592 | 0.954925 | 1.101143 | 0.02615 | 0.976798 | 0.116505 | 0.103941 | 0.009709 | 0.009709 | 2224.107 | 104.1614 | 9 |
| L | 9 | 0.8051 | 0.800098 | 2.091498 | 0.070934 | 0.757969 | 0.106796 | 0.096326 | 0.009709 | 0.009709 | 2560.737 | 440.7908 | 3 |
| Q | 9 | 0.8961 | 0.895073 | 2.262484 | 0.050731 | 1.613433 | 0.116505 | 0.103941 | 0.009709 | 0.009709 | 2437.585 | 317.6391 | 3 |
| H | 9 | 0.9455 | 0.939325 | 0.913739 | 0.032288 | 0.697418 | 0.106796 | 0.096326 | 0.009709 | 0.009709 | 2442.949 | 323.0033 | 21 |
| P | 9 | 0.7557 | 0.755092 | 6.062269 | 0.117289 | 2.22359 | 0.106796 | 0.096326 | 0.009709 | 0.009709 | 2553.428 | 433.4816 | 3 |
| T | 9 | 0.9124 | 0.902135 | 0.829859 | 0.022465 | 0.779733 | 0.097087 | 0.088521 | 0.009709 | 0.009709 | 2421.487 | 301.5412 | 6 |
| LQ | 9 | 0.9452 | 0.944046 | 1.178384 | 0.028764 | 1.000342 | 0.106796 | 0.096326 | 0.009709 | 0.009709 | 2317.073 | 197.1272 | 6 |
| LH | 9 | 0.9236 | 0.914977 | 0.913567 | 0.03183 | 0.706984 | 0.106796 | 0.096326 | 0.009709 | 0.009709 | 2445.818 | 325.8723 | 20 |
| LP | 9 | 0.8827 | 0.880539 | 1.396405 | 0.054626 | 0.578676 | 0.106796 | 0.096326 | 0.009709 | 0.009709 | 2446.215 | 326.2688 | 5 |
| LT | 9 | 0.9093 | 0.896425 | 1.096503 | 0.044509 | 0.73629 | 0.135922 | 0.118599 | 0.009709 | 0.009709 | 2435.645 | 315.6996 | 9 |
| QH | 9 | 0.9484 | 0.946663 | 1.286445 | 0.029652 | 1.092107 | 0.106796 | 0.096326 | 0.009709 | 0.009709 | 2308.114 | 188.168 | 16 |
| QP | 9 | 0.9098 | 0.907219 | 2.311025 | 0.049469 | 1.786142 | 0.106796 | 0.096326 | 0.009709 | 0.009709 | 2414.063 | 294.1171 | 6 |
| QT | 9 | 0.9505 | 0.945247 | 1.345963 | 0.031598 | 1.146001 | 0.116505 | 0.103941 | 0.009709 | 0.009709 | 2267.607 | 147.6614 | 7 |
| HP | 9 | 0.9422 | 0.938199 | 0.930143 | 0.02783 | 0.751676 | 0.106796 | 0.096326 | 0.009709 | 0.009709 | 2395.204 | 275.2586 | 24 |
| HT | 9 | 0.9452 | 0.934919 | 1.395365 | 0.037834 | 1.154212 | 0.106796 | 0.096326 | 0.009709 | 0.009709 | 2390.734 | 270.7884 | 13 |
| PT | 9 | 0.9256 | 0.912687 | 1.458011 | 0.046336 | 1.062792 | 0.145631 | 0.125642 | 0.009709 | 0.009709 | 2377.534 | 257.5886 | 9 |
| LQH | 9 | 0.9492 | 0.947196 | 1.132508 | 0.027591 | 0.969926 | 0.106796 | 0.096326 | 0.009709 | 0.009709 | 2310.08 | 190.1336 | 11 |
| LQP | 9 | 0.9435 | 0.941687 | 1.217183 | 0.03118 | 1.011261 | 0.106796 | 0.096326 | 0.009709 | 0.009709 | 2301.767 | 181.8209 | 8 |
| LQT | 9 | 0.9522 | 0.949378 | 1.139054 | 0.027263 | 0.984669 | 0.106796 | 0.096326 | 0.009709 | 0.009709 | 2278.927 | 158.9808 | 7 |
| LHP | 9 | 0.9608 | 0.957938 | 0.648894 | 0.020462 | 0.570321 | 0.106796 | 0.096326 | 0.009709 | 0.009709 | 2304.172 | 184.2257 | 19 |
| LHT | 9 | 0.9395 | 0.925244 | 1.140388 | 0.03549 | 0.960091 | 0.116505 | 0.103941 | 0.009709 | 0.009709 | 2439.355 | 319.4093 | 27 |
| LPT | 9 | 0.9654 | 0.953835 | 1.08305 | 0.027026 | 0.996645 | 0.126214 | 0.111365 | 0.009709 | 0.009709 | 2265.184 | 145.2377 | 11 |
| QHP | 9 | 0.9483 | 0.946897 | 1.317318 | 0.029967 | 1.127801 | 0.106796 | 0.096326 | 0.009709 | 0.009709 | 2295.716 | 175.7701 | 15 |
| QHT | 9 | 0.9514 | 0.946827 | 1.327543 | 0.030878 | 1.137677 | 0.106796 | 0.096326 | 0.009709 | 0.009709 | 2267.095 | 147.1487 | 10 |
| QPT | 9 | 0.9525 | 0.947388 | 1.422464 | 0.031156 | 1.237189 | 0.106796 | 0.096326 | 0.009709 | 0.009709 | 2261.875 | 141.9291 | 8 |
| HPT | 9 | 0.9501 | 0.940404 | 1.31543 | 0.029951 | 1.171607 | 0.116505 | 0.103941 | 0.009709 | 0.009709 | 2368.798 | 248.8516 | 26 |
| LQHP | 9 | 0.9521 | 0.949449 | 1.123604 | 0.027521 | 0.971224 | 0.106796 | 0.096326 | 0.009709 | 0.009709 | 2283.554 | 163.6082 | 15 |
| LQHT | 9 | 0.9517 | 0.948906 | 1.151474 | 0.027685 | 0.992992 | 0.116505 | 0.103941 | 0.009709 | 0.009709 | 2279.654 | 159.7079 | 9 |
| LQPT | 9 | 0.9581 | 0.954219 | 1.108656 | 0.02634 | 0.979358 | 0.116505 | 0.103941 | 0.009709 | 0.009709 | 2228.539 | 108.5936 | 8 |
| LHPT | 9 | 0.9671 | 0.958733 | 0.953378 | 0.024384 | 0.87317 | 0.126214 | 0.111365 | 0.009709 | 0.009709 | 2254.583 | 134.6374 | 14 |
| QHPT | 9 | 0.9519 | 0.947416 | 1.398207 | 0.030969 | 1.215587 | 0.106796 | 0.096326 | 0.009709 | 0.009709 | 2266.447 | 146.5012 | 13 |
| LQHPT | 9 | 0.9581 | 0.954222 | 1.108811 | 0.026337 | 0.979673 | 0.116505 | 0.103941 | 0.009709 | 0.009709 | 2231.081 | 111.1351 | 9 |
| L | 9.5 | 0.8048 | 0.799871 | 2.124942 | 0.071526 | 0.77021 | 0.116505 | 0.103941 | 0.009709 | 0.009709 | 2560.868 | 440.9224 | 3 |
| Q | 9.5 | 0.8961 | 0.895037 | 2.261132 | 0.050719 | 1.612214 | 0.116505 | 0.103941 | 0.009709 | 0.009709 | 2437.721 | 317.7752 | 3 |
| H | 9.5 | 0.9357 | 0.925042 | 0.787316 | 0.029621 | 0.632094 | 0.106796 | 0.096326 | 0.009709 | 0.009709 | 2441.964 | 322.0181 | 14 |
| P | 9.5 | 0.7558 | 0.755181 | 6.054587 | 0.117204 | 2.221049 | 0.106796 | 0.096326 | 0.009709 | 0.009709 | 2553.476 | 433.5304 | 3 |
| T | 9.5 | 0.9124 | 0.902135 | 0.829859 | 0.022465 | 0.779733 | 0.097087 | 0.088521 | 0.009709 | 0.009709 | 2426.931 | 306.9855 | 6 |
| LQ | 9.5 | 0.9452 | 0.944003 | 1.179877 | 0.0288 | 1.001273 | 0.106796 | 0.096326 | 0.009709 | 0.009709 | 2315.434 | 195.4884 | 5 |
| LH | 9.5 | 0.913 | 0.905285 | 0.925532 | 0.035134 | 0.652169 | 0.126214 | 0.111365 | 0.009709 | 0.009709 | 2450.979 | 331.0332 | 15 |
| LP | 9.5 | 0.8821 | 0.87991 | 1.414871 | 0.055062 | 0.584664 | 0.106796 | 0.096326 | 0.009709 | 0.009709 | 2446.667 | 326.7215 | 5 |
| LT | 9.5 | 0.9079 | 0.89555 | 1.096394 | 0.045045 | 0.72315 | 0.135922 | 0.118599 | 0.009709 | 0.009709 | 2439.498 | 319.5521 | 8 |
| QH | 9.5 | 0.9471 | 0.945214 | 1.30496 | 0.030383 | 1.099685 | 0.106796 | 0.096326 | 0.009709 | 0.009709 | 2320.486 | 200.5405 | 18 |
| QP | 9.5 | 0.9097 | 0.907089 | 2.309897 | 0.049429 | 1.785807 | 0.106796 | 0.096326 | 0.009709 | 0.009709 | 2414.311 | 294.3655 | 6 |
| QT | 9.5 | 0.9493 | 0.944259 | 1.355081 | 0.031992 | 1.147806 | 0.106796 | 0.096326 | 0.009709 | 0.009709 | 2272.325 | 152.3787 | 7 |
| HP | 9.5 | 0.9373 | 0.932652 | 0.948493 | 0.030972 | 0.724569 | 0.106796 | 0.096326 | 0.009709 | 0.009709 | 2391.08 | 271.1337 | 18 |
| HT | 9.5 | 0.9393 | 0.927731 | 1.320445 | 0.037738 | 1.085649 | 0.116505 | 0.103941 | 0.009709 | 0.009709 | 2404.688 | 284.7426 | 11 |
| PT | 9.5 | 0.9228 | 0.910284 | 1.486906 | 0.047478 | 1.06395 | 0.145631 | 0.125642 | 0.009709 | 0.009709 | 2384.144 | 264.1981 | 9 |
| LQH | 9.5 | 0.9487 | 0.946781 | 1.137994 | 0.027698 | 0.974034 | 0.106796 | 0.096326 | 0.009709 | 0.009709 | 2312.035 | 192.0887 | 10 |
| LQP | 9.5 | 0.9432 | 0.941317 | 1.231743 | 0.031386 | 1.023013 | 0.106796 | 0.096326 | 0.009709 | 0.009709 | 2303.221 | 183.2753 | 8 |
| LQT | 9.5 | 0.9516 | 0.948964 | 1.138868 | 0.027382 | 0.982013 | 0.116505 | 0.103941 | 0.009709 | 0.009709 | 2282.374 | 162.428 | 7 |
| LHP | 9.5 | 0.959 | 0.955988 | 0.66561 | 0.020911 | 0.581376 | 0.106796 | 0.096326 | 0.009709 | 0.009709 | 2309.554 | 189.6081 | 17 |
| LHT | 9.5 | 0.9295 | 0.915729 | 1.062824 | 0.036758 | 0.848396 | 0.116505 | 0.103941 | 0.009709 | 0.009709 | 2409.856 | 289.9105 | 12 |
| LPT | 9.5 | 0.964 | 0.95281 | 1.068333 | 0.027037 | 0.980045 | 0.126214 | 0.111365 | 0.009709 | 0.009709 | 2273.14 | 153.1937 | 10 |
| QHP | 9.5 | 0.948 | 0.946673 | 1.329976 | 0.030145 | 1.137528 | 0.106796 | 0.096326 | 0.009709 | 0.009709 | 2296.342 | 176.3964 | 14 |
| QHT | 9.5 | 0.9504 | 0.946057 | 1.325185 | 0.03106 | 1.129 | 0.116505 | 0.103941 | 0.009709 | 0.009709 | 2270.82 | 150.874 | 9 |
| QPT | 9.5 | 0.9522 | 0.94712 | 1.435312 | 0.031327 | 1.247116 | 0.106796 | 0.096326 | 0.009709 | 0.009709 | 2266.859 | 146.9131 | 8 |
| HPT | 9.5 | 0.9463 | 0.93608 | 1.317694 | 0.031505 | 1.148361 | 0.126214 | 0.111365 | 0.009709 | 0.009709 | 2361.291 | 241.3455 | 19 |
| LQHP | 9.5 | 0.9504 | 0.94804 | 1.14248 | 0.02808 | 0.982412 | 0.106796 | 0.096326 | 0.009709 | 0.009709 | 2291.997 | 172.0507 | 15 |
| LQHT | 9.5 | 0.9513 | 0.948572 | 1.14904 | 0.027725 | 0.9893 | 0.116505 | 0.103941 | 0.009709 | 0.009709 | 2284.53 | 164.5836 | 9 |
| LQPT | 9.5 | 0.9574 | 0.953747 | 1.114909 | 0.026484 | 0.982401 | 0.116505 | 0.103941 | 0.009709 | 0.009709 | 2233.506 | 113.5604 | 8 |
| LHPT | 9.5 | 0.9664 | 0.957716 | 0.974855 | 0.024828 | 0.892427 | 0.116505 | 0.103941 | 0.009709 | 0.009709 | 2272.241 | 152.2955 | 17 |
| QHPT | 9.5 | 0.9516 | 0.947166 | 1.406668 | 0.031114 | 1.221683 | 0.106796 | 0.096326 | 0.009709 | 0.009709 | 2267.676 | 147.7302 | 12 |
| LQHPT | 9.5 | 0.9574 | 0.953768 | 1.114632 | 0.026465 | 0.98225 | 0.116505 | 0.103941 | 0.009709 | 0.009709 | 2233.659 | 113.7134 | 8 |
| L | 10 | 0.8045 | 0.799586 | 2.157855 | 0.07212 | 0.782735 | 0.106796 | 0.096326 | 0.009709 | 0.009709 | 2561.007 | 441.0613 | 3 |
| Q | 10 | 0.896 | 0.894976 | 2.260001 | 0.05073 | 1.611047 | 0.116505 | 0.103941 | 0.009709 | 0.009709 | 2437.864 | 317.9177 | 3 |
| H | 10 | 0.9219 | 0.920387 | 0.726745 | 0.024857 | 0.569003 | 0.106796 | 0.096326 | 0.009709 | 0.009709 | 2444.089 | 324.1434 | 10 |
| P | 10 | 0.7559 | 0.755247 | 6.048515 | 0.117156 | 2.21924 | 0.106796 | 0.096326 | 0.009709 | 0.009709 | 2553.528 | 433.5817 | 3 |
| T | 10 | 0.9124 | 0.902135 | 0.829859 | 0.022465 | 0.779733 | 0.097087 | 0.088521 | 0.009709 | 0.009709 | 2432.563 | 312.617 | 6 |
| LQ | 10 | 0.9451 | 0.943995 | 1.181771 | 0.028829 | 1.002635 | 0.106796 | 0.096326 | 0.009709 | 0.009709 | 2315.89 | 195.9439 | 5 |
| LH | 10 | 0.9019 | 0.893939 | 0.998307 | 0.039521 | 0.629691 | 0.116505 | 0.103941 | 0.009709 | 0.009709 | 2461.184 | 341.2381 | 12 |
| LP | 10 | 0.8815 | 0.879294 | 1.434222 | 0.055498 | 0.591024 | 0.106796 | 0.096326 | 0.009709 | 0.009709 | 2447.142 | 327.1958 | 5 |
| LT | 10 | 0.9068 | 0.894501 | 1.097343 | 0.045594 | 0.711841 | 0.135922 | 0.118599 | 0.009709 | 0.009709 | 2445.347 | 325.4008 | 8 |
| QH | 10 | 0.9457 | 0.943722 | 1.321127 | 0.031138 | 1.103468 | 0.106796 | 0.096326 | 0.009709 | 0.009709 | 2321.351 | 201.405 | 16 |
| QP | 10 | 0.9095 | 0.906981 | 2.306674 | 0.049361 | 1.783412 | 0.106796 | 0.096326 | 0.009709 | 0.009709 | 2414.577 | 294.6307 | 6 |
| QT | 10 | 0.9482 | 0.94373 | 1.362794 | 0.03213 | 1.150128 | 0.106796 | 0.096326 | 0.009709 | 0.009709 | 2274.755 | 154.8091 | 6 |
| HP | 10 | 0.9309 | 0.925934 | 0.987295 | 0.034761 | 0.700077 | 0.135922 | 0.118599 | 0.009709 | 0.009709 | 2396.188 | 276.2419 | 14 |
| HT | 10 | 0.934 | 0.906592 | 1.105815 | 0.036459 | 1.022496 | 0.116505 | 0.103941 | 0.009709 | 0.009709 | 2413.343 | 293.3968 | 8 |
| PT | 10 | 0.9202 | 0.908528 | 1.514613 | 0.048235 | 1.069946 | 0.135922 | 0.118599 | 0.009709 | 0.009709 | 2388.395 | 268.4494 | 8 |
| LQH | 10 | 0.9482 | 0.94643 | 1.143951 | 0.027772 | 0.978251 | 0.106796 | 0.096326 | 0.009709 | 0.009709 | 2312.517 | 192.5715 | 9 |
| LQP | 10 | 0.9429 | 0.941229 | 1.235669 | 0.031436 | 1.025941 | 0.106796 | 0.096326 | 0.009709 | 0.009709 | 2304.65 | 184.704 | 8 |
| LQT | 10 | 0.951 | 0.948643 | 1.138645 | 0.027446 | 0.97973 | 0.116505 | 0.103941 | 0.009709 | 0.009709 | 2285.811 | 165.8647 | 7 |
| LHP | 10 | 0.9569 | 0.953742 | 0.678892 | 0.021507 | 0.587966 | 0.106796 | 0.096326 | 0.009709 | 0.009709 | 2317.402 | 197.4564 | 16 |
| LHT | 10 | 0.9183 | 0.905743 | 1.01419 | 0.039152 | 0.748869 | 0.135922 | 0.118599 | 0.009709 | 0.009709 | 2420.884 | 300.9379 | 9 |
| LPT | 10 | 0.9628 | 0.951898 | 1.052778 | 0.02711 | 0.961061 | 0.126214 | 0.111365 | 0.009709 | 0.009709 | 2279.545 | 159.5993 | 9 |
| QHP | 10 | 0.9478 | 0.946329 | 1.344773 | 0.030408 | 1.149342 | 0.106796 | 0.096326 | 0.009709 | 0.009709 | 2302.607 | 182.6615 | 15 |
| QHT | 10 | 0.9493 | 0.945021 | 1.325559 | 0.031411 | 1.122241 | 0.116505 | 0.103941 | 0.009709 | 0.009709 | 2277.152 | 157.2064 | 9 |
| QPT | 10 | 0.9519 | 0.946877 | 1.447701 | 0.031499 | 1.256495 | 0.106796 | 0.096326 | 0.009709 | 0.009709 | 2267.891 | 147.9453 | 7 |
| HPT | 10 | 0.9416 | 0.930286 | 1.329488 | 0.034883 | 1.115002 | 0.126214 | 0.111365 | 0.009709 | 0.009709 | 2366.357 | 246.4116 | 15 |
| LQHP | 10 | 0.9493 | 0.946954 | 1.153642 | 0.028566 | 0.985853 | 0.116505 | 0.103941 | 0.009709 | 0.009709 | 2295.686 | 175.74 | 14 |
| LQHT | 10 | 0.951 | 0.94851 | 1.142746 | 0.027571 | 0.98278 | 0.116505 | 0.103941 | 0.009709 | 0.009709 | 2284.693 | 164.747 | 7 |
| LQPT | 10 | 0.9567 | 0.953169 | 1.120734 | 0.026706 | 0.984931 | 0.116505 | 0.103941 | 0.009709 | 0.009709 | 2238.697 | 118.7511 | 8 |
| LHPT | 10 | 0.9655 | 0.956564 | 0.980842 | 0.025245 | 0.896143 | 0.116505 | 0.103941 | 0.009709 | 0.009709 | 2282.07 | 162.1241 | 17 |
| QHPT | 10 | 0.9513 | 0.946879 | 1.415514 | 0.03128 | 1.228096 | 0.106796 | 0.096326 | 0.009709 | 0.009709 | 2269.045 | 149.0989 | 11 |
| LQHPT | 10 | 0.9568 | 0.953178 | 1.121292 | 0.026704 | 0.985496 | 0.116505 | 0.103941 | 0.009709 | 0.009709 | 2238.646 | 118.7002 | 8 |

**Table S4.4.** Raw model evaluation outputs from ENMeval (Muscarella et al. 2014) of the preliminary models using partial occurrences (D*_n_* + D*_s_*).

| **Features** | **RM** | **Full AUC** | **Mean AUC** | **Var AUC** | **Mean AUC**  **DIFF** | **Var AUC**  **DIFF** | **Mean**  **OR10** | **Var**  **OR10** | **Mean**  **ORmin** | **Var**  **ORmin** | **AICc** | **Delta**  **AICc** | **# Par** |
| --- | --- | --- | --- | --- | --- | --- | --- | --- | --- | --- | --- | --- | --- |
| L | 0.5 | 0.8064 | 0.800833 | 1.564904 | 0.060466 | 0.559499 | 0.106796 | 0.096326 | 0.009709 | 0.009709 | 2559.713 | 439.7675 | 3 |
| Q | 0.5 | 0.8967 | 0.895609 | 2.270158 | 0.050571 | 1.629139 | 0.106796 | 0.096326 | 0.009709 | 0.009709 | 2436.734 | 316.7877 | 3 |
| H | 0.5 | 0.9792 | 0.976022 | 0.445704 | 0.014269 | 0.415131 | 0.106796 | 0.096326 | 0.009709 | 0.009709 | 3065.729 | 945.7829 | 85 |
| P | 0.5 | 0.7549 | 0.754321 | 6.133358 | 0.117958 | 2.249614 | 0.106796 | 0.096326 | 0.009709 | 0.009709 | 2553.062 | 433.1158 | 3 |
| T | 0.5 | 0.9785 | 0.955973 | 1.751469 | 0.034085 | 1.676121 | 0.165049 | 0.139159 | 0.009709 | 0.009709 | 2613.922 | 493.9761 | 75 |
| LQ | 0.5 | 0.9459 | 0.944762 | 1.135217 | 0.027909 | 0.965198 | 0.106796 | 0.096326 | 0.009709 | 0.009709 | 2310.91 | 190.9645 | 6 |
| LH | 0.5 | 0.9795 | 0.975994 | 0.447052 | 0.014313 | 0.416317 | 0.106796 | 0.096326 | 0.009709 | 0.009709 | 2704.329 | 584.3826 | 78 |
| LP | 0.5 | 0.89 | 0.887909 | 1.15416 | 0.048451 | 0.510127 | 0.106796 | 0.096326 | 0.009709 | 0.009709 | 2443.424 | 323.4778 | 6 |
| LT | 0.5 | 0.9785 | 0.955973 | 1.751469 | 0.034085 | 1.676121 | 0.165049 | 0.139159 | 0.009709 | 0.009709 | 2613.922 | 493.9761 | 75 |
| QH | 0.5 | 0.9793 | 0.975577 | 0.458759 | 0.014444 | 0.428217 | 0.106796 | 0.096326 | 0.009709 | 0.009709 | 2416.02 | 296.0738 | 66 |
| QP | 0.5 | 0.9099 | 0.907473 | 2.359163 | 0.05054 | 1.811429 | 0.106796 | 0.096326 | 0.009709 | 0.009709 | 2412.5 | 292.5542 | 6 |
| QT | 0.5 | 0.9794 | 0.957235 | 1.619609 | 0.03314 | 1.551376 | 0.165049 | 0.139159 | 0.009709 | 0.009709 | 2620.025 | 500.079 | 75 |
| HP | 0.5 | 0.9795 | 0.976401 | 0.424116 | 0.013945 | 0.394768 | 0.106796 | 0.096326 | 0.009709 | 0.009709 | 2822.292 | 702.3464 | 81 |
| HT | 0.5 | 0.9822 | 0.962794 | 1.063416 | 0.028343 | 1.014615 | 0.165049 | 0.139159 | 0.009709 | 0.009709 | 2527.219 | 407.2729 | 72 |
| PT | 0.5 | 0.9785 | 0.955972 | 1.751461 | 0.034085 | 1.676121 | 0.165049 | 0.139159 | 0.009709 | 0.009709 | 2613.922 | 493.9761 | 75 |
| LQH | 0.5 | 0.9793 | 0.975482 | 0.471393 | 0.014566 | 0.440679 | 0.106796 | 0.096326 | 0.009709 | 0.009709 | 2278.763 | 158.8173 | 55 |
| LQP | 0.5 | 0.9554 | 0.953167 | 0.790971 | 0.024297 | 0.67021 | 0.106796 | 0.096326 | 0.009709 | 0.009709 | 2249.69 | 129.7445 | 9 |
| LQT | 0.5 | 0.9794 | 0.957235 | 1.619609 | 0.03314 | 1.551376 | 0.165049 | 0.139159 | 0.009709 | 0.009709 | 2620.025 | 500.079 | 75 |
| LHP | 0.5 | 0.9799 | 0.976314 | 0.42894 | 0.013964 | 0.399843 | 0.106796 | 0.096326 | 0.009709 | 0.009709 | 2458.736 | 338.7898 | 69 |
| LHT | 0.5 | 0.9822 | 0.962794 | 1.063416 | 0.028343 | 1.014615 | 0.165049 | 0.139159 | 0.009709 | 0.009709 | 2527.219 | 407.2729 | 72 |
| LPT | 0.5 | 0.9785 | 0.955972 | 1.751461 | 0.034085 | 1.676121 | 0.165049 | 0.139159 | 0.009709 | 0.009709 | 2613.922 | 493.9761 | 75 |
| QHP | 0.5 | 0.9802 | 0.976473 | 0.407314 | 0.013781 | 0.378967 | 0.106796 | 0.096326 | 0.009709 | 0.009709 | 2661.873 | 541.9274 | 77 |
| QHT | 0.5 | 0.9822 | 0.962794 | 1.063416 | 0.028343 | 1.014615 | 0.165049 | 0.139159 | 0.009709 | 0.009709 | 2527.219 | 407.2729 | 72 |
| QPT | 0.5 | 0.9794 | 0.957235 | 1.619609 | 0.03314 | 1.551376 | 0.165049 | 0.139159 | 0.009709 | 0.009709 | 2620.025 | 500.079 | 75 |
| HPT | 0.5 | 0.9822 | 0.962792 | 1.063405 | 0.028343 | 1.014615 | 0.165049 | 0.139159 | 0.009709 | 0.009709 | 2527.219 | 407.2729 | 72 |
| LQHP | 0.5 | 0.9801 | 0.976275 | 0.429805 | 0.013978 | 0.401124 | 0.106796 | 0.096326 | 0.009709 | 0.009709 | 2359.979 | 240.0329 | 63 |
| LQHT | 0.5 | 0.9822 | 0.962794 | 1.063416 | 0.028343 | 1.014615 | 0.165049 | 0.139159 | 0.009709 | 0.009709 | 2527.219 | 407.2729 | 72 |
| LQPT | 0.5 | 0.9794 | 0.957235 | 1.619609 | 0.03314 | 1.551376 | 0.165049 | 0.139159 | 0.009709 | 0.009709 | 2620.025 | 500.079 | 75 |
| LHPT | 0.5 | 0.9822 | 0.962792 | 1.063405 | 0.028343 | 1.014615 | 0.165049 | 0.139159 | 0.009709 | 0.009709 | 2527.219 | 407.2729 | 72 |
| QHPT | 0.5 | 0.9822 | 0.96279 | 1.063391 | 0.028343 | 1.014615 | 0.165049 | 0.139159 | 0.009709 | 0.009709 | 2527.219 | 407.2729 | 72 |
| LQHPT | 0.5 | 0.9822 | 0.96279 | 1.063391 | 0.028343 | 1.014615 | 0.165049 | 0.139159 | 0.009709 | 0.009709 | 2527.219 | 407.2729 | 72 |
| L | 1 | 0.8064 | 0.800837 | 1.574355 | 0.060688 | 0.563392 | 0.106796 | 0.096326 | 0.009709 | 0.009709 | 2559.714 | 439.7683 | 3 |
| Q | 1 | 0.8966 | 0.89559 | 2.269841 | 0.050578 | 1.628525 | 0.106796 | 0.096326 | 0.009709 | 0.009709 | 2436.725 | 316.779 | 3 |
| H | 1 | 0.9767 | 0.974593 | 0.489915 | 0.014889 | 0.452674 | 0.106796 | 0.096326 | 0.009709 | 0.009709 | 4482.584 | 2362.638 | 94 |
| P | 1 | 0.7549 | 0.754321 | 6.133358 | 0.117958 | 2.249614 | 0.106796 | 0.096326 | 0.009709 | 0.009709 | 2553.062 | 433.1158 | 3 |
| T | 1 | 0.9766 | 0.957582 | 1.648864 | 0.031755 | 1.574322 | 0.184466 | 0.151913 | 0.009709 | 0.009709 | 2173.102 | 53.15574 | 50 |
| LQ | 1 | 0.9459 | 0.94476 | 1.135532 | 0.027912 | 0.965573 | 0.106796 | 0.096326 | 0.009709 | 0.009709 | 2310.935 | 190.9886 | 6 |
| LH | 1 | 0.9767 | 0.974582 | 0.491656 | 0.014913 | 0.454319 | 0.106796 | 0.096326 | 0.009709 | 0.009709 | NA | NA | 102 |
| LP | 1 | 0.8899 | 0.887832 | 1.157562 | 0.048541 | 0.511079 | 0.106796 | 0.096326 | 0.009709 | 0.009709 | 2443.436 | 323.4905 | 6 |
| LT | 1 | 0.9771 | 0.958465 | 1.576088 | 0.030948 | 1.505424 | 0.194175 | 0.158005 | 0.009709 | 0.009709 | 2188.334 | 68.38788 | 52 |
| QH | 1 | 0.9763 | 0.972891 | 0.757783 | 0.016258 | 0.717658 | 0.106796 | 0.096326 | 0.009709 | 0.009709 | 2397.276 | 277.3301 | 63 |
| QP | 1 | 0.9099 | 0.907484 | 2.357746 | 0.050517 | 1.810771 | 0.106796 | 0.096326 | 0.009709 | 0.009709 | 2412.47 | 292.5241 | 6 |
| QT | 1 | 0.9748 | 0.960679 | 1.376384 | 0.02876 | 1.296969 | 0.165049 | 0.139159 | 0.009709 | 0.009709 | 2135.219 | 15.27326 | 41 |
| HP | 1 | 0.9773 | 0.974649 | 0.464033 | 0.014816 | 0.427726 | 0.116505 | 0.103941 | 0.009709 | 0.009709 | NA | NA | 102 |
| HT | 1 | 0.9775 | 0.963335 | 1.23552 | 0.02658 | 1.174762 | 0.174757 | 0.145631 | 0.009709 | 0.009709 | 4108.782 | 1988.836 | 93 |
| PT | 1 | 0.9777 | 0.961058 | 1.31323 | 0.028915 | 1.24517 | 0.184466 | 0.151913 | 0.009709 | 0.009709 | 2162.013 | 42.06752 | 47 |
| LQH | 1 | 0.9762 | 0.972716 | 0.768211 | 0.016317 | 0.727883 | 0.106796 | 0.096326 | 0.009709 | 0.009709 | 2397.679 | 277.7326 | 63 |
| LQP | 1 | 0.9552 | 0.953035 | 0.799563 | 0.024384 | 0.677776 | 0.106796 | 0.096326 | 0.009709 | 0.009709 | 2248.399 | 128.4527 | 8 |
| LQT | 1 | 0.9749 | 0.961114 | 1.339105 | 0.028339 | 1.261925 | 0.15534 | 0.132496 | 0.009709 | 0.009709 | 2134.003 | 14.05725 | 41 |
| LHP | 1 | 0.9778 | 0.975408 | 0.452482 | 0.0141 | 0.418329 | 0.116505 | 0.103941 | 0.009709 | 0.009709 | 2180.457 | 60.51129 | 39 |
| LHT | 1 | 0.9774 | 0.963332 | 1.235695 | 0.026586 | 1.174957 | 0.174757 | 0.145631 | 0.009709 | 0.009709 | 5976.576 | 3856.631 | 97 |
| LPT | 1 | 0.9776 | 0.962253 | 1.246133 | 0.027842 | 1.17667 | 0.174757 | 0.145631 | 0.009709 | 0.009709 | 2135.308 | 15.3622 | 41 |
| QHP | 1 | 0.977 | 0.973404 | 0.631623 | 0.015773 | 0.592524 | 0.116505 | 0.103941 | 0.009709 | 0.009709 | 3160.513 | 1040.567 | 86 |
| QHT | 1 | 0.9761 | 0.964551 | 1.147034 | 0.025195 | 1.085743 | 0.165049 | 0.139159 | 0.009709 | 0.009709 | 2174.971 | 55.02537 | 48 |
| QPT | 1 | 0.9752 | 0.96128 | 1.396612 | 0.028392 | 1.320174 | 0.15534 | 0.132496 | 0.009709 | 0.009709 | 2132.911 | 12.96462 | 41 |
| HPT | 1 | 0.979 | 0.96648 | 0.944684 | 0.024189 | 0.890409 | 0.165049 | 0.139159 | 0.009709 | 0.009709 | 2827.828 | 707.8822 | 82 |
| LQHP | 1 | 0.9783 | 0.975062 | 0.52102 | 0.014393 | 0.487469 | 0.116505 | 0.103941 | 0.009709 | 0.009709 | 2224.263 | 104.3168 | 47 |
| LQHT | 1 | 0.9762 | 0.964551 | 1.146797 | 0.025202 | 1.085373 | 0.165049 | 0.139159 | 0.009709 | 0.009709 | 2197.653 | 77.70666 | 51 |
| LQPT | 1 | 0.9764 | 0.962593 | 1.30337 | 0.027416 | 1.232174 | 0.145631 | 0.125642 | 0.009709 | 0.009709 | 2131.225 | 11.27886 | 41 |
| LHPT | 1 | 0.9792 | 0.96745 | 0.900575 | 0.023334 | 0.849869 | 0.174757 | 0.145631 | 0.009709 | 0.009709 | 2275.67 | 155.7239 | 60 |
| QHPT | 1 | 0.9771 | 0.965222 | 1.148498 | 0.024934 | 1.090019 | 0.145631 | 0.125642 | 0.009709 | 0.009709 | 2202.562 | 82.61575 | 52 |
| LQHPT | 1 | 0.9779 | 0.966314 | 1.026133 | 0.023962 | 0.97063 | 0.145631 | 0.125642 | 0.009709 | 0.009709 | 2176.972 | 57.02585 | 49 |
| L | 1.5 | 0.8064 | 0.800881 | 1.604631 | 0.061388 | 0.575654 | 0.106796 | 0.096326 | 0.009709 | 0.009709 | 2559.715 | 439.7689 | 3 |
| Q | 1.5 | 0.8966 | 0.89555 | 2.269753 | 0.050596 | 1.627642 | 0.106796 | 0.096326 | 0.009709 | 0.009709 | 2436.704 | 316.7582 | 3 |
| H | 1.5 | 0.9749 | 0.972871 | 0.539331 | 0.015942 | 0.494998 | 0.116505 | 0.103941 | 0.009709 | 0.009709 | NA | NA | 145 |
| P | 1.5 | 0.7549 | 0.75432 | 6.133323 | 0.117958 | 2.249616 | 0.106796 | 0.096326 | 0.009709 | 0.009709 | 2553.062 | 433.116 | 3 |
| T | 1.5 | 0.9759 | 0.95614 | 1.715875 | 0.03259 | 1.636179 | 0.184466 | 0.151913 | 0.009709 | 0.009709 | 2149.061 | 29.11553 | 39 |
| LQ | 1.5 | 0.9459 | 0.944778 | 1.138886 | 0.027946 | 0.968588 | 0.106796 | 0.096326 | 0.009709 | 0.009709 | 2311.037 | 191.0913 | 6 |
| LH | 1.5 | 0.9749 | 0.972823 | 0.532855 | 0.015961 | 0.488558 | 0.116505 | 0.103941 | 0.009709 | 0.009709 | NA | NA | 145 |
| LP | 1.5 | 0.8895 | 0.887471 | 1.17065 | 0.048885 | 0.515036 | 0.106796 | 0.096326 | 0.009709 | 0.009709 | 2443.498 | 323.5517 | 6 |
| LT | 1.5 | 0.9759 | 0.957432 | 1.584747 | 0.031342 | 1.508154 | 0.184466 | 0.151913 | 0.009709 | 0.009709 | 2172.147 | 52.20134 | 43 |
| QH | 1.5 | 0.9726 | 0.970367 | 0.856252 | 0.017542 | 0.803021 | 0.106796 | 0.096326 | 0.009709 | 0.009709 | 2468.94 | 348.994 | 66 |
| QP | 1.5 | 0.91 | 0.907546 | 2.353459 | 0.050433 | 1.808674 | 0.106796 | 0.096326 | 0.009709 | 0.009709 | 2412.433 | 292.487 | 6 |
| QT | 1.5 | 0.9735 | 0.961641 | 1.225634 | 0.027007 | 1.147304 | 0.145631 | 0.125642 | 0.009709 | 0.009709 | 2124.875 | 4.929095 | 30 |
| HP | 1.5 | 0.9754 | 0.972666 | 0.490416 | 0.015899 | 0.446903 | 0.106796 | 0.096326 | 0.009709 | 0.009709 | NA | NA | 113 |
| HT | 1.5 | 0.9754 | 0.965333 | 1.091999 | 0.023946 | 1.031534 | 0.145631 | 0.125642 | 0.009709 | 0.009709 | 5327.386 | 3207.44 | 96 |
| PT | 1.5 | 0.9762 | 0.960552 | 1.255594 | 0.028703 | 1.183653 | 0.15534 | 0.132496 | 0.009709 | 0.009709 | 2141.75 | 21.80382 | 37 |
| LQH | 1.5 | 0.9722 | 0.970502 | 0.832462 | 0.017379 | 0.779612 | 0.106796 | 0.096326 | 0.009709 | 0.009709 | 2275.313 | 155.3671 | 48 |
| LQP | 1.5 | 0.9551 | 0.952801 | 0.824206 | 0.024543 | 0.70095 | 0.106796 | 0.096326 | 0.009709 | 0.009709 | 2252.604 | 132.6586 | 9 |
| LQT | 1.5 | 0.9736 | 0.961603 | 1.225238 | 0.02703 | 1.147263 | 0.145631 | 0.125642 | 0.009709 | 0.009709 | 2128.815 | 8.869149 | 31 |
| LHP | 1.5 | 0.9758 | 0.973353 | 0.478315 | 0.015263 | 0.437017 | 0.106796 | 0.096326 | 0.009709 | 0.009709 | 2188.657 | 68.71127 | 37 |
| LHT | 1.5 | 0.9754 | 0.965336 | 1.091755 | 0.023944 | 1.031258 | 0.145631 | 0.125642 | 0.009709 | 0.009709 | 5327.386 | 3207.44 | 96 |
| LPT | 1.5 | 0.9768 | 0.963112 | 1.062433 | 0.026324 | 0.996251 | 0.145631 | 0.125642 | 0.009709 | 0.009709 | 2127.497 | 7.550768 | 33 |
| QHP | 1.5 | 0.9736 | 0.969762 | 0.858474 | 0.018332 | 0.804657 | 0.106796 | 0.096326 | 0.009709 | 0.009709 | 2786.618 | 666.6717 | 79 |
| QHT | 1.5 | 0.9749 | 0.966199 | 1.022152 | 0.022866 | 0.962735 | 0.15534 | 0.132496 | 0.009709 | 0.009709 | 2328.877 | 208.9313 | 60 |
| QPT | 1.5 | 0.9737 | 0.961301 | 1.282642 | 0.027417 | 1.203831 | 0.135922 | 0.118599 | 0.009709 | 0.009709 | 2129.981 | 10.03484 | 33 |
| HPT | 1.5 | 0.9774 | 0.966397 | 0.904882 | 0.023316 | 0.8498 | 0.145631 | 0.125642 | 0.009709 | 0.009709 | 2729.284 | 609.3378 | 79 |
| LQHP | 1.5 | 0.9757 | 0.972581 | 0.60554 | 0.015987 | 0.562026 | 0.116505 | 0.103941 | 0.009709 | 0.009709 | 2203.251 | 83.30528 | 39 |
| LQHT | 1.5 | 0.9749 | 0.966223 | 1.022106 | 0.022832 | 0.962721 | 0.145631 | 0.125642 | 0.009709 | 0.009709 | 2341.186 | 221.2405 | 61 |
| LQPT | 1.5 | 0.9763 | 0.963128 | 1.183669 | 0.026192 | 1.114953 | 0.126214 | 0.111365 | 0.009709 | 0.009709 | 2128.07 | 8.124409 | 34 |
| LHPT | 1.5 | 0.978 | 0.967865 | 0.834812 | 0.021922 | 0.784325 | 0.145631 | 0.125642 | 0.009709 | 0.009709 | 2215.753 | 95.80725 | 49 |
| QHPT | 1.5 | 0.9757 | 0.965443 | 1.061365 | 0.023688 | 1.002004 | 0.135922 | 0.118599 | 0.009709 | 0.009709 | 2306.022 | 186.0763 | 59 |
| LQHPT | 1.5 | 0.9775 | 0.967666 | 0.895116 | 0.021847 | 0.84556 | 0.116505 | 0.103941 | 0.009709 | 0.009709 | 2167.887 | 47.9414 | 43 |
| L | 2 | 0.8065 | 0.800879 | 1.634799 | 0.062085 | 0.587706 | 0.106796 | 0.096326 | 0.009709 | 0.009709 | 2559.723 | 439.7773 | 3 |
| Q | 2 | 0.8966 | 0.895485 | 2.269972 | 0.05064 | 1.627054 | 0.106796 | 0.096326 | 0.009709 | 0.009709 | 2436.699 | 316.7534 | 3 |
| H | 2 | 0.973 | 0.970575 | 0.601332 | 0.017328 | 0.548763 | 0.106796 | 0.096326 | 0.009709 | 0.009709 | NA | NA | 130 |
| P | 2 | 0.7549 | 0.754312 | 6.133631 | 0.117969 | 2.249774 | 0.106796 | 0.096326 | 0.009709 | 0.009709 | 2553.065 | 433.119 | 3 |
| T | 2 | 0.975 | 0.955258 | 1.719246 | 0.032602 | 1.637756 | 0.184466 | 0.151913 | 0.009709 | 0.009709 | 2171.466 | 51.51978 | 36 |
| LQ | 2 | 0.9459 | 0.944783 | 1.141558 | 0.02799 | 0.970898 | 0.106796 | 0.096326 | 0.009709 | 0.009709 | 2311.189 | 191.2431 | 6 |
| LH | 2 | 0.973 | 0.970665 | 0.599468 | 0.017284 | 0.547003 | 0.106796 | 0.096326 | 0.009709 | 0.009709 | NA | NA | 130 |
| LP | 2 | 0.8892 | 0.887111 | 1.184553 | 0.049249 | 0.519487 | 0.106796 | 0.096326 | 0.009709 | 0.009709 | 2443.615 | 323.6687 | 6 |
| LT | 2 | 0.9742 | 0.956954 | 1.579699 | 0.030899 | 1.499079 | 0.174757 | 0.145631 | 0.009709 | 0.009709 | 2171.897 | 51.95127 | 36 |
| QH | 2 | 0.9694 | 0.967558 | 0.912786 | 0.019088 | 0.845941 | 0.116505 | 0.103941 | 0.009709 | 0.009709 | 2409.618 | 289.6718 | 60 |
| QP | 2 | 0.9101 | 0.907612 | 2.351084 | 0.050383 | 1.807621 | 0.106796 | 0.096326 | 0.009709 | 0.009709 | 2412.427 | 292.4814 | 6 |
| QT | 2 | 0.9722 | 0.961457 | 1.195329 | 0.026326 | 1.113215 | 0.135922 | 0.118599 | 0.009709 | 0.009709 | 2141.83 | 21.88444 | 25 |
| HP | 2 | 0.9731 | 0.970378 | 0.549587 | 0.017289 | 0.497018 | 0.106796 | 0.096326 | 0.009709 | 0.009709 | NA | NA | 105 |
| HT | 2 | 0.974 | 0.964742 | 1.134517 | 0.024303 | 1.067335 | 0.145631 | 0.125642 | 0.009709 | 0.009709 | 2765.394 | 645.4483 | 79 |
| PT | 2 | 0.9747 | 0.9595 | 1.254313 | 0.028863 | 1.177721 | 0.174757 | 0.145631 | 0.009709 | 0.009709 | 2155.778 | 35.83193 | 33 |
| LQH | 2 | 0.9696 | 0.968147 | 0.881059 | 0.01878 | 0.816395 | 0.106796 | 0.096326 | 0.009709 | 0.009709 | 2223.826 | 103.8798 | 35 |
| LQP | 2 | 0.954 | 0.951729 | 0.871137 | 0.025279 | 0.741403 | 0.116505 | 0.103941 | 0.009709 | 0.009709 | 2257.754 | 137.8086 | 9 |
| LQT | 2 | 0.9722 | 0.961573 | 1.174149 | 0.026208 | 1.092316 | 0.135922 | 0.118599 | 0.009709 | 0.009709 | 2141.83 | 21.88444 | 25 |
| LHP | 2 | 0.9744 | 0.971687 | 0.493465 | 0.016085 | 0.447649 | 0.106796 | 0.096326 | 0.009709 | 0.009709 | 2199.198 | 79.25182 | 36 |
| LHT | 2 | 0.9742 | 0.964648 | 1.146396 | 0.02441 | 1.079318 | 0.145631 | 0.125642 | 0.009709 | 0.009709 | 2902.013 | 782.0671 | 82 |
| LPT | 2 | 0.9762 | 0.962931 | 0.997539 | 0.026043 | 0.929036 | 0.15534 | 0.132496 | 0.009709 | 0.009709 | 2121.944 | 1.997624 | 25 |
| QHP | 2 | 0.9704 | 0.966823 | 0.935241 | 0.019951 | 0.866356 | 0.106796 | 0.096326 | 0.009709 | 0.009709 | 2423.301 | 303.3551 | 62 |
| QHT | 2 | 0.9733 | 0.965588 | 1.030356 | 0.022752 | 0.964415 | 0.135922 | 0.118599 | 0.009709 | 0.009709 | 2241.803 | 121.8571 | 47 |
| QPT | 2 | 0.9719 | 0.961151 | 1.243568 | 0.02667 | 1.159844 | 0.126214 | 0.111365 | 0.009709 | 0.009709 | 2136.525 | 16.57901 | 26 |
| HPT | 2 | 0.9759 | 0.96488 | 0.995169 | 0.024291 | 0.932265 | 0.145631 | 0.125642 | 0.009709 | 0.009709 | 2950.18 | 830.2343 | 83 |
| LQHP | 2 | 0.9746 | 0.9718 | 0.621433 | 0.015951 | 0.576491 | 0.116505 | 0.103941 | 0.009709 | 0.009709 | 2158.773 | 38.8273 | 24 |
| LQHT | 2 | 0.9732 | 0.965596 | 1.021246 | 0.022725 | 0.95517 | 0.135922 | 0.118599 | 0.009709 | 0.009709 | 2256.013 | 136.0674 | 49 |
| LQPT | 2 | 0.9763 | 0.964001 | 1.097806 | 0.024726 | 1.032494 | 0.126214 | 0.111365 | 0.009709 | 0.009709 | 2119.946 | 0 | 24 |
| LHPT | 2 | 0.9774 | 0.967593 | 0.794497 | 0.021774 | 0.741691 | 0.165049 | 0.139159 | 0.009709 | 0.009709 | 2163.327 | 43.38075 | 37 |
| QHPT | 2 | 0.973 | 0.964718 | 1.074119 | 0.023386 | 1.005874 | 0.135922 | 0.118599 | 0.009709 | 0.009709 | 2237.522 | 117.5764 | 48 |
| LQHPT | 2 | 0.9759 | 0.967501 | 0.942294 | 0.021504 | 0.888125 | 0.126214 | 0.111365 | 0.009709 | 0.009709 | 2148.669 | 28.72299 | 33 |
| L | 2.5 | 0.8064 | 0.800916 | 1.664934 | 0.062755 | 0.599634 | 0.106796 | 0.096326 | 0.009709 | 0.009709 | 2559.739 | 439.7935 | 3 |
| Q | 2.5 | 0.8965 | 0.89546 | 2.270366 | 0.050679 | 1.62619 | 0.106796 | 0.096326 | 0.009709 | 0.009709 | 2436.698 | 316.7521 | 3 |
| H | 2.5 | 0.9708 | 0.968338 | 0.66394 | 0.018673 | 0.60111 | 0.116505 | 0.103941 | 0.009709 | 0.009709 | NA | NA | 119 |
| P | 2.5 | 0.7549 | 0.754315 | 6.133248 | 0.117966 | 2.249578 | 0.106796 | 0.096326 | 0.009709 | 0.009709 | 2553.069 | 433.1232 | 3 |
| T | 2.5 | 0.9747 | 0.953844 | 1.795407 | 0.033501 | 1.710703 | 0.174757 | 0.145631 | 0.009709 | 0.009709 | 2171.523 | 51.57714 | 27 |
| LQ | 2.5 | 0.9459 | 0.944761 | 1.14374 | 0.028028 | 0.972979 | 0.106796 | 0.096326 | 0.009709 | 0.009709 | 2311.367 | 191.4208 | 6 |
| LH | 2.5 | 0.9709 | 0.968311 | 0.666424 | 0.018729 | 0.603154 | 0.116505 | 0.103941 | 0.009709 | 0.009709 | NA | NA | 116 |
| LP | 2.5 | 0.8888 | 0.886724 | 1.198776 | 0.049627 | 0.52399 | 0.106796 | 0.096326 | 0.009709 | 0.009709 | 2443.743 | 323.7968 | 6 |
| LT | 2.5 | 0.9732 | 0.956038 | 1.52123 | 0.03071 | 1.440386 | 0.184466 | 0.151913 | 0.009709 | 0.009709 | 2198.032 | 78.08586 | 34 |
| QH | 2.5 | 0.9661 | 0.964255 | 0.970053 | 0.020766 | 0.888822 | 0.116505 | 0.103941 | 0.009709 | 0.009709 | 2487.911 | 367.9648 | 64 |
| QP | 2.5 | 0.9101 | 0.907671 | 2.348233 | 0.050332 | 1.806262 | 0.106796 | 0.096326 | 0.009709 | 0.009709 | 2412.424 | 292.478 | 6 |
| QT | 2.5 | 0.9703 | 0.960294 | 1.198052 | 0.026463 | 1.107439 | 0.135922 | 0.118599 | 0.009709 | 0.009709 | 2148.206 | 28.2602 | 18 |
| HP | 2.5 | 0.9706 | 0.967906 | 0.598839 | 0.018765 | 0.534065 | 0.106796 | 0.096326 | 0.009709 | 0.009709 | NA | NA | 119 |
| HT | 2.5 | 0.9732 | 0.963494 | 1.186579 | 0.024937 | 1.115243 | 0.15534 | 0.132496 | 0.009709 | 0.009709 | 2983.028 | 863.0821 | 83 |
| PT | 2.5 | 0.9738 | 0.958794 | 1.238551 | 0.028913 | 1.158837 | 0.165049 | 0.139159 | 0.009709 | 0.009709 | 2156.626 | 36.6805 | 25 |
| LQH | 2.5 | 0.9673 | 0.966776 | 0.903302 | 0.019436 | 0.833917 | 0.106796 | 0.096326 | 0.009709 | 0.009709 | 2198.516 | 78.5705 | 23 |
| LQP | 2.5 | 0.9531 | 0.95071 | 0.920046 | 0.025949 | 0.784304 | 0.116505 | 0.103941 | 0.009709 | 0.009709 | 2262.365 | 142.4191 | 9 |
| LQT | 2.5 | 0.9703 | 0.960364 | 1.157778 | 0.02636 | 1.067756 | 0.135922 | 0.118599 | 0.009709 | 0.009709 | 2151.519 | 31.57315 | 19 |
| LHP | 2.5 | 0.9726 | 0.970135 | 0.493962 | 0.016802 | 0.441228 | 0.106796 | 0.096326 | 0.009709 | 0.009709 | 2203.774 | 83.82799 | 33 |
| LHT | 2.5 | 0.9732 | 0.963322 | 1.194911 | 0.025107 | 1.123336 | 0.15534 | 0.132496 | 0.009709 | 0.009709 | 2983.028 | 863.0821 | 83 |
| LPT | 2.5 | 0.9749 | 0.961254 | 1.037664 | 0.026886 | 0.96388 | 0.145631 | 0.125642 | 0.009709 | 0.009709 | 2146.359 | 26.41318 | 25 |
| QHP | 2.5 | 0.9654 | 0.962702 | 1.02646 | 0.022171 | 0.935068 | 0.116505 | 0.103941 | 0.009709 | 0.009709 | 2417.202 | 297.2559 | 59 |
| QHT | 2.5 | 0.9713 | 0.963995 | 1.063298 | 0.023376 | 0.987519 | 0.126214 | 0.111365 | 0.009709 | 0.009709 | 2210.185 | 90.23943 | 37 |
| QPT | 2.5 | 0.9701 | 0.960089 | 1.228332 | 0.026747 | 1.136481 | 0.126214 | 0.111365 | 0.009709 | 0.009709 | 2153.003 | 33.05708 | 22 |
| HPT | 2.5 | 0.9741 | 0.963166 | 1.083696 | 0.025091 | 1.014228 | 0.145631 | 0.125642 | 0.009709 | 0.009709 | 2422.44 | 302.4943 | 64 |
| LQHP | 2.5 | 0.9752 | 0.972664 | 0.595254 | 0.015157 | 0.552757 | 0.126214 | 0.111365 | 0.009709 | 0.009709 | 2147.399 | 27.45357 | 21 |
| LQHT | 2.5 | 0.9713 | 0.964241 | 1.026818 | 0.023132 | 0.951622 | 0.126214 | 0.111365 | 0.009709 | 0.009709 | 2195.94 | 75.99402 | 34 |
| LQPT | 2.5 | 0.9748 | 0.963836 | 1.032473 | 0.024097 | 0.964471 | 0.135922 | 0.118599 | 0.009709 | 0.009709 | 2137.537 | 17.59078 | 22 |
| LHPT | 2.5 | 0.976 | 0.965672 | 0.837175 | 0.022787 | 0.778276 | 0.145631 | 0.125642 | 0.009709 | 0.009709 | 2164.469 | 44.52283 | 32 |
| QHPT | 2.5 | 0.9707 | 0.962985 | 1.108407 | 0.024096 | 1.029011 | 0.126214 | 0.111365 | 0.009709 | 0.009709 | 2233.812 | 113.8663 | 43 |
| LQHPT | 2.5 | 0.9747 | 0.967086 | 0.890695 | 0.021158 | 0.831774 | 0.116505 | 0.103941 | 0.009709 | 0.009709 | 2172.785 | 52.8388 | 32 |
| L | 3 | 0.8064 | 0.800945 | 1.695398 | 0.063421 | 0.611491 | 0.106796 | 0.096326 | 0.009709 | 0.009709 | 2559.763 | 439.8174 | 3 |
| Q | 3 | 0.8965 | 0.895439 | 2.270609 | 0.050706 | 1.625395 | 0.106796 | 0.096326 | 0.009709 | 0.009709 | 2436.7 | 316.7538 | 3 |
| H | 3 | 0.9682 | 0.965632 | 0.742965 | 0.020396 | 0.666075 | 0.126214 | 0.111365 | 0.009709 | 0.009709 | NA | NA | 115 |
| P | 3 | 0.7549 | 0.754319 | 6.132423 | 0.117958 | 2.249301 | 0.106796 | 0.096326 | 0.009709 | 0.009709 | 2553.074 | 433.1285 | 3 |
| T | 3 | 0.9744 | 0.954023 | 1.605979 | 0.033025 | 1.520542 | 0.203883 | 0.163906 | 0.009709 | 0.009709 | 2208.207 | 88.26134 | 26 |
| LQ | 3 | 0.9459 | 0.944738 | 1.146574 | 0.02807 | 0.975527 | 0.106796 | 0.096326 | 0.009709 | 0.009709 | 2311.568 | 191.6221 | 6 |
| LH | 3 | 0.9683 | 0.965536 | 0.756444 | 0.020494 | 0.678502 | 0.126214 | 0.111365 | 0.009709 | 0.009709 | NA | NA | 113 |
| LP | 3 | 0.8884 | 0.88631 | 1.213156 | 0.050006 | 0.528734 | 0.106796 | 0.096326 | 0.009709 | 0.009709 | 2443.909 | 323.963 | 6 |
| LT | 3 | 0.9723 | 0.955126 | 1.442216 | 0.030638 | 1.359615 | 0.213592 | 0.169617 | 0.009709 | 0.009709 | 2192.545 | 72.59912 | 23 |
| QH | 3 | 0.9626 | 0.960979 | 1.026004 | 0.02249 | 0.927075 | 0.106796 | 0.096326 | 0.009709 | 0.009709 | 2338.468 | 218.522 | 46 |
| QP | 3 | 0.9102 | 0.907728 | 2.344954 | 0.050271 | 1.804618 | 0.106796 | 0.096326 | 0.009709 | 0.009709 | 2412.424 | 292.4778 | 6 |
| QT | 3 | 0.9676 | 0.958865 | 1.204812 | 0.026791 | 1.102028 | 0.126214 | 0.111365 | 0.009709 | 0.009709 | 2168.03 | 48.08441 | 15 |
| HP | 3 | 0.9679 | 0.964743 | 0.703821 | 0.020711 | 0.62296 | 0.116505 | 0.103941 | 0.009709 | 0.009709 | NA | NA | 108 |
| HT | 3 | 0.9718 | 0.96201 | 1.262854 | 0.025825 | 1.184721 | 0.15534 | 0.132496 | 0.009709 | 0.009709 | 2581.247 | 461.3011 | 71 |
| PT | 3 | 0.9725 | 0.957151 | 1.251322 | 0.029447 | 1.168494 | 0.174757 | 0.145631 | 0.009709 | 0.009709 | 2182.36 | 62.41425 | 23 |
| LQH | 3 | 0.9672 | 0.96566 | 0.92274 | 0.019983 | 0.848008 | 0.106796 | 0.096326 | 0.009709 | 0.009709 | 2193.526 | 73.58052 | 19 |
| LQP | 3 | 0.9521 | 0.949605 | 0.973053 | 0.026649 | 0.830504 | 0.106796 | 0.096326 | 0.009709 | 0.009709 | 2266.454 | 146.5085 | 9 |
| LQT | 3 | 0.9677 | 0.959046 | 1.148727 | 0.026546 | 1.047769 | 0.126214 | 0.111365 | 0.009709 | 0.009709 | 2175.063 | 55.1167 | 17 |
| LHP | 3 | 0.9712 | 0.968538 | 0.518429 | 0.017513 | 0.459686 | 0.106796 | 0.096326 | 0.009709 | 0.009709 | 2199.587 | 79.64159 | 28 |
| LHT | 3 | 0.9718 | 0.961917 | 1.270486 | 0.025921 | 1.192322 | 0.15534 | 0.132496 | 0.009709 | 0.009709 | 3071.138 | 951.1916 | 84 |
| LPT | 3 | 0.9735 | 0.959935 | 1.061524 | 0.027277 | 0.983199 | 0.15534 | 0.132496 | 0.009709 | 0.009709 | 2156.94 | 36.99454 | 21 |
| QHP | 3 | 0.9612 | 0.959139 | 1.092797 | 0.024293 | 0.979345 | 0.106796 | 0.096326 | 0.009709 | 0.009709 | 2291.823 | 171.8769 | 39 |
| QHT | 3 | 0.9687 | 0.962005 | 1.10688 | 0.024399 | 1.0163 | 0.126214 | 0.111365 | 0.009709 | 0.009709 | 2209.298 | 89.35193 | 31 |
| QPT | 3 | 0.9677 | 0.958825 | 1.227688 | 0.026902 | 1.125706 | 0.135922 | 0.118599 | 0.009709 | 0.009709 | 2156.918 | 36.97246 | 15 |
| HPT | 3 | 0.9718 | 0.961075 | 1.151373 | 0.026081 | 1.073433 | 0.145631 | 0.125642 | 0.009709 | 0.009709 | 2496.972 | 377.0265 | 67 |
| LQHP | 3 | 0.9746 | 0.971413 | 0.608678 | 0.015964 | 0.561949 | 0.116505 | 0.103941 | 0.009709 | 0.009709 | 2145.905 | 25.95877 | 19 |
| LQHT | 3 | 0.969 | 0.962537 | 1.054102 | 0.023921 | 0.966055 | 0.135922 | 0.118599 | 0.009709 | 0.009709 | 2207.129 | 87.18265 | 30 |
| LQPT | 3 | 0.9741 | 0.96377 | 0.981504 | 0.023557 | 0.912083 | 0.126214 | 0.111365 | 0.009709 | 0.009709 | 2145.026 | 25.08019 | 19 |
| LHPT | 3 | 0.9743 | 0.963395 | 0.868857 | 0.023888 | 0.802446 | 0.15534 | 0.132496 | 0.009709 | 0.009709 | 2180.606 | 60.65986 | 30 |
| QHPT | 3 | 0.9681 | 0.96084 | 1.159576 | 0.0254 | 1.063647 | 0.126214 | 0.111365 | 0.009709 | 0.009709 | 2223.329 | 103.3832 | 36 |
| LQHPT | 3 | 0.9752 | 0.966639 | 0.892951 | 0.021069 | 0.832053 | 0.126214 | 0.111365 | 0.009709 | 0.009709 | 2134.427 | 14.48081 | 20 |
| L | 3.5 | 0.8065 | 0.80092 | 1.727128 | 0.064108 | 0.624086 | 0.106796 | 0.096326 | 0.009709 | 0.009709 | 2559.796 | 439.8498 | 3 |
| Q | 3.5 | 0.8965 | 0.895381 | 2.271332 | 0.050753 | 1.62501 | 0.106796 | 0.096326 | 0.009709 | 0.009709 | 2436.704 | 316.7584 | 3 |
| H | 3.5 | 0.9648 | 0.962133 | 0.853462 | 0.022761 | 0.754592 | 0.106796 | 0.096326 | 0.009709 | 0.009709 | 2982.187 | 862.2412 | 81 |
| P | 3.5 | 0.7549 | 0.754306 | 6.132489 | 0.11797 | 2.249259 | 0.106796 | 0.096326 | 0.009709 | 0.009709 | 2553.081 | 433.1349 | 3 |
| T | 3.5 | 0.9742 | 0.952333 | 1.624392 | 0.033679 | 1.542706 | 0.203883 | 0.163906 | 0.009709 | 0.009709 | 2229.229 | 109.2827 | 21 |
| LQ | 3.5 | 0.9458 | 0.944694 | 1.150526 | 0.028143 | 0.978914 | 0.106796 | 0.096326 | 0.009709 | 0.009709 | 2311.795 | 191.8493 | 6 |
| LH | 3.5 | 0.9648 | 0.962233 | 0.827537 | 0.022608 | 0.729403 | 0.106796 | 0.096326 | 0.009709 | 0.009709 | 3733.097 | 1613.151 | 90 |
| LP | 3.5 | 0.888 | 0.885876 | 1.22755 | 0.050407 | 0.532966 | 0.106796 | 0.096326 | 0.009709 | 0.009709 | 2444.109 | 324.1636 | 6 |
| LT | 3.5 | 0.9715 | 0.954575 | 1.384193 | 0.030554 | 1.299719 | 0.213592 | 0.169617 | 0.009709 | 0.009709 | 2216.85 | 96.90373 | 20 |
| QH | 3.5 | 0.9591 | 0.957394 | 1.088305 | 0.024537 | 0.966302 | 0.106796 | 0.096326 | 0.009709 | 0.009709 | 2284.008 | 164.0616 | 31 |
| QP | 3.5 | 0.9102 | 0.907761 | 2.341772 | 0.050214 | 1.802969 | 0.106796 | 0.096326 | 0.009709 | 0.009709 | 2412.429 | 292.483 | 6 |
| QT | 3.5 | 0.9645 | 0.956933 | 1.226437 | 0.027398 | 1.108324 | 0.135922 | 0.118599 | 0.009709 | 0.009709 | 2178.704 | 58.75773 | 10 |
| HP | 3.5 | 0.965 | 0.962243 | 0.75434 | 0.022046 | 0.660509 | 0.106796 | 0.096326 | 0.009709 | 0.009709 | 4592.34 | 2472.394 | 94 |
| HT | 3.5 | 0.9697 | 0.960491 | 1.306459 | 0.026572 | 1.220011 | 0.135922 | 0.118599 | 0.009709 | 0.009709 | 2679.487 | 559.5411 | 74 |
| PT | 3.5 | 0.9704 | 0.954796 | 1.244328 | 0.030071 | 1.155244 | 0.174757 | 0.145631 | 0.009709 | 0.009709 | 2214.267 | 94.32131 | 22 |
| LQH | 3.5 | 0.9649 | 0.963466 | 0.95441 | 0.021208 | 0.868217 | 0.116505 | 0.103941 | 0.009709 | 0.009709 | 2204.54 | 84.59426 | 16 |
| LQP | 3.5 | 0.951 | 0.948682 | 0.999159 | 0.027208 | 0.849547 | 0.106796 | 0.096326 | 0.009709 | 0.009709 | 2271.149 | 151.2031 | 9 |
| LQT | 3.5 | 0.9649 | 0.957352 | 1.158161 | 0.027083 | 1.043805 | 0.126214 | 0.111365 | 0.009709 | 0.009709 | 2190.453 | 70.50673 | 13 |
| LHP | 3.5 | 0.9705 | 0.96809 | 0.539118 | 0.017708 | 0.47834 | 0.106796 | 0.096326 | 0.009709 | 0.009709 | 2188.907 | 68.96104 | 22 |
| LHT | 3.5 | 0.9697 | 0.960409 | 1.309782 | 0.026653 | 1.223385 | 0.135922 | 0.118599 | 0.009709 | 0.009709 | 3034.891 | 914.9447 | 83 |
| LPT | 3.5 | 0.9727 | 0.95959 | 1.071892 | 0.027173 | 0.992427 | 0.145631 | 0.125642 | 0.009709 | 0.009709 | 2164.42 | 44.47394 | 18 |
| QHP | 3.5 | 0.9573 | 0.955724 | 1.149494 | 0.026129 | 1.014927 | 0.106796 | 0.096326 | 0.009709 | 0.009709 | 2235.682 | 115.7366 | 19 |
| QHT | 3.5 | 0.9653 | 0.958834 | 1.175396 | 0.02606 | 1.064466 | 0.135922 | 0.118599 | 0.009709 | 0.009709 | 2193.431 | 73.48499 | 19 |
| QPT | 3.5 | 0.9644 | 0.956627 | 1.240205 | 0.027385 | 1.123472 | 0.135922 | 0.118599 | 0.009709 | 0.009709 | 2173.268 | 53.32201 | 12 |
| HPT | 3.5 | 0.9695 | 0.95906 | 1.202369 | 0.026896 | 1.114185 | 0.126214 | 0.111365 | 0.009709 | 0.009709 | 3423.488 | 1303.542 | 88 |
| LQHP | 3.5 | 0.9732 | 0.970051 | 0.665679 | 0.016899 | 0.612804 | 0.106796 | 0.096326 | 0.009709 | 0.009709 | 2152.243 | 32.29663 | 17 |
| LQHT | 3.5 | 0.966 | 0.959787 | 1.107455 | 0.025252 | 1.003438 | 0.126214 | 0.111365 | 0.009709 | 0.009709 | 2227.713 | 107.7676 | 28 |
| LQPT | 3.5 | 0.9731 | 0.963878 | 0.941325 | 0.0231 | 0.871207 | 0.145631 | 0.125642 | 0.009709 | 0.009709 | 2139.904 | 19.95824 | 13 |
| LHPT | 3.5 | 0.9732 | 0.962519 | 0.901494 | 0.024293 | 0.832257 | 0.126214 | 0.111365 | 0.009709 | 0.009709 | 2184.61 | 64.66424 | 27 |
| QHPT | 3.5 | 0.9648 | 0.957926 | 1.207842 | 0.026705 | 1.093053 | 0.126214 | 0.111365 | 0.009709 | 0.009709 | 2210.159 | 90.21302 | 27 |
| LQHPT | 3.5 | 0.9749 | 0.967047 | 0.883967 | 0.020782 | 0.824511 | 0.135922 | 0.118599 | 0.009709 | 0.009709 | 2144.605 | 24.65928 | 20 |
| L | 4 | 0.8064 | 0.800983 | 1.756719 | 0.064696 | 0.634792 | 0.106796 | 0.096326 | 0.009709 | 0.009709 | 2559.841 | 439.8955 | 3 |
| Q | 4 | 0.8964 | 0.895328 | 2.272028 | 0.050793 | 1.624836 | 0.106796 | 0.096326 | 0.009709 | 0.009709 | 2436.712 | 316.7659 | 3 |
| H | 4 | 0.9624 | 0.960497 | 0.894117 | 0.02368 | 0.784625 | 0.106796 | 0.096326 | 0.009709 | 0.009709 | 3108.377 | 988.4311 | 83 |
| P | 4 | 0.755 | 0.754348 | 6.128748 | 0.117939 | 2.248274 | 0.106796 | 0.096326 | 0.009709 | 0.009709 | 2553.098 | 433.1518 | 3 |
| T | 4 | 0.9719 | 0.953637 | 1.487235 | 0.032115 | 1.394361 | 0.213592 | 0.169617 | 0.009709 | 0.009709 | 2255.731 | 135.7847 | 18 |
| LQ | 4 | 0.9458 | 0.944647 | 1.153632 | 0.028202 | 0.981505 | 0.106796 | 0.096326 | 0.009709 | 0.009709 | 2312.074 | 192.1281 | 6 |
| LH | 4 | 0.9626 | 0.960424 | 0.873765 | 0.023642 | 0.76437 | 0.106796 | 0.096326 | 0.009709 | 0.009709 | 3108.715 | 988.7696 | 83 |
| LP | 4 | 0.8876 | 0.88554 | 1.240495 | 0.05071 | 0.536888 | 0.106796 | 0.096326 | 0.009709 | 0.009709 | 2444.367 | 324.4213 | 6 |
| LT | 4 | 0.9705 | 0.952975 | 1.327745 | 0.030851 | 1.242198 | 0.194175 | 0.158005 | 0.009709 | 0.009709 | 2253.065 | 133.1195 | 20 |
| QH | 4 | 0.9557 | 0.95461 | 1.131906 | 0.025799 | 0.994102 | 0.106796 | 0.096326 | 0.009709 | 0.009709 | 2260.996 | 141.0504 | 19 |
| QP | 4 | 0.9103 | 0.907828 | 2.33929 | 0.050148 | 1.801914 | 0.106796 | 0.096326 | 0.009709 | 0.009709 | 2412.462 | 292.5164 | 6 |
| QT | 4 | 0.961 | 0.954156 | 1.263004 | 0.028388 | 1.126737 | 0.126214 | 0.111365 | 0.009709 | 0.009709 | 2200.308 | 80.36235 | 10 |
| HP | 4 | 0.9633 | 0.960577 | 0.787181 | 0.022766 | 0.685431 | 0.106796 | 0.096326 | 0.009709 | 0.009709 | NA | NA | 113 |
| HT | 4 | 0.9676 | 0.958529 | 1.326494 | 0.02746 | 1.227595 | 0.135922 | 0.118599 | 0.009709 | 0.009709 | 2868.059 | 748.1128 | 79 |
| PT | 4 | 0.9689 | 0.953685 | 1.276199 | 0.030176 | 1.183974 | 0.174757 | 0.145631 | 0.009709 | 0.009709 | 2233.85 | 113.9044 | 19 |
| LQH | 4 | 0.9621 | 0.960784 | 0.99163 | 0.022608 | 0.89129 | 0.106796 | 0.096326 | 0.009709 | 0.009709 | 2220.657 | 100.7116 | 14 |
| LQP | 4 | 0.9499 | 0.947663 | 1.048577 | 0.02787 | 0.891235 | 0.106796 | 0.096326 | 0.009709 | 0.009709 | 2275.699 | 155.7536 | 9 |
| LQT | 4 | 0.9618 | 0.955159 | 1.17688 | 0.027697 | 1.049621 | 0.116505 | 0.103941 | 0.009709 | 0.009709 | 2212.32 | 92.37388 | 12 |
| LHP | 4 | 0.9696 | 0.967594 | 0.530372 | 0.017857 | 0.468304 | 0.106796 | 0.096326 | 0.009709 | 0.009709 | 2225.893 | 105.9467 | 29 |
| LHT | 4 | 0.9676 | 0.958495 | 1.327865 | 0.027492 | 1.229188 | 0.135922 | 0.118599 | 0.009709 | 0.009709 | 4055.657 | 1935.711 | 92 |
| LPT | 4 | 0.9722 | 0.959311 | 1.083078 | 0.02706 | 1.003134 | 0.145631 | 0.125642 | 0.009709 | 0.009709 | 2171.107 | 51.16146 | 16 |
| QHP | 4 | 0.9549 | 0.953288 | 1.173809 | 0.026913 | 1.027449 | 0.116505 | 0.103941 | 0.009709 | 0.009709 | 2245 | 125.0541 | 17 |
| QHT | 4 | 0.9607 | 0.954417 | 1.263975 | 0.028085 | 1.12597 | 0.126214 | 0.111365 | 0.009709 | 0.009709 | 2227.751 | 107.805 | 21 |
| QPT | 4 | 0.9609 | 0.953745 | 1.264687 | 0.028433 | 1.128675 | 0.126214 | 0.111365 | 0.009709 | 0.009709 | 2198.667 | 78.7213 | 13 |
| HPT | 4 | 0.9661 | 0.956398 | 1.257881 | 0.027879 | 1.154784 | 0.126214 | 0.111365 | 0.009709 | 0.009709 | 3451.7 | 1331.754 | 88 |
| LQHP | 4 | 0.971 | 0.968453 | 0.707389 | 0.017875 | 0.649024 | 0.106796 | 0.096326 | 0.009709 | 0.009709 | 2182.118 | 62.17205 | 21 |
| LQHT | 4 | 0.9625 | 0.956553 | 1.173063 | 0.026659 | 1.051251 | 0.135922 | 0.118599 | 0.009709 | 0.009709 | 2232.216 | 112.2701 | 21 |
| LQPT | 4 | 0.9719 | 0.963496 | 0.952504 | 0.023064 | 0.87845 | 0.126214 | 0.111365 | 0.009709 | 0.009709 | 2147.436 | 27.48985 | 12 |
| LHPT | 4 | 0.9726 | 0.962105 | 0.908999 | 0.024098 | 0.839564 | 0.126214 | 0.111365 | 0.009709 | 0.009709 | 2186.589 | 66.64328 | 24 |
| QHPT | 4 | 0.9603 | 0.953377 | 1.270797 | 0.028629 | 1.130233 | 0.135922 | 0.118599 | 0.009709 | 0.009709 | 2232.047 | 112.1008 | 25 |
| LQHPT | 4 | 0.9735 | 0.966053 | 0.915229 | 0.02127 | 0.849487 | 0.126214 | 0.111365 | 0.009709 | 0.009709 | 2150.905 | 30.95937 | 18 |
| L | 4.5 | 0.8065 | 0.800995 | 1.78867 | 0.065329 | 0.646663 | 0.106796 | 0.096326 | 0.009709 | 0.009709 | 2559.895 | 439.9494 | 3 |
| Q | 4.5 | 0.8964 | 0.895298 | 2.271957 | 0.050823 | 1.623699 | 0.106796 | 0.096326 | 0.009709 | 0.009709 | 2436.722 | 316.7764 | 3 |
| H | 4.5 | 0.9619 | 0.960121 | 0.904518 | 0.02391 | 0.791516 | 0.106796 | 0.096326 | 0.009709 | 0.009709 | 2794.372 | 674.4264 | 75 |
| P | 4.5 | 0.755 | 0.754402 | 6.123438 | 0.117891 | 2.246628 | 0.106796 | 0.096326 | 0.009709 | 0.009709 | 2553.119 | 433.1728 | 3 |
| T | 4.5 | 0.968 | 0.948768 | 1.453788 | 0.033063 | 1.356324 | 0.184466 | 0.151913 | 0.009709 | 0.009709 | 2285.937 | 165.9916 | 16 |
| LQ | 4.5 | 0.9458 | 0.944595 | 1.157502 | 0.02827 | 0.984853 | 0.106796 | 0.096326 | 0.009709 | 0.009709 | 2312.37 | 192.4243 | 6 |
| LH | 4.5 | 0.962 | 0.959949 | 0.891563 | 0.023908 | 0.779158 | 0.106796 | 0.096326 | 0.009709 | 0.009709 | 3122.893 | 1002.947 | 83 |
| LP | 4.5 | 0.8873 | 0.885116 | 1.257734 | 0.051101 | 0.543728 | 0.106796 | 0.096326 | 0.009709 | 0.009709 | 2444.658 | 324.7116 | 6 |
| LT | 4.5 | 0.9674 | 0.94943 | 1.25912 | 0.031487 | 1.16906 | 0.203883 | 0.163906 | 0.009709 | 0.009709 | 2289.129 | 169.1834 | 18 |
| QH | 4.5 | 0.9551 | 0.953972 | 1.14699 | 0.026051 | 1.005546 | 0.106796 | 0.096326 | 0.009709 | 0.009709 | 2269.305 | 149.359 | 20 |
| QP | 4.5 | 0.9103 | 0.907879 | 2.336943 | 0.05009 | 1.800652 | 0.106796 | 0.096326 | 0.009709 | 0.009709 | 2412.51 | 292.5639 | 6 |
| QT | 4.5 | 0.9575 | 0.951708 | 1.290536 | 0.02918 | 1.138981 | 0.116505 | 0.103941 | 0.009709 | 0.009709 | 2217.7 | 97.75426 | 9 |
| HP | 4.5 | 0.9614 | 0.958719 | 0.811839 | 0.02341 | 0.702257 | 0.106796 | 0.096326 | 0.009709 | 0.009709 | 3169.287 | 1049.342 | 84 |
| HT | 4.5 | 0.9645 | 0.955246 | 1.384014 | 0.029324 | 1.266023 | 0.116505 | 0.103941 | 0.009709 | 0.009709 | 2683.283 | 563.3371 | 72 |
| PT | 4.5 | 0.9669 | 0.951364 | 1.28639 | 0.030045 | 1.197944 | 0.174757 | 0.145631 | 0.009709 | 0.009709 | 2252.803 | 132.857 | 16 |
| LQH | 4.5 | 0.9589 | 0.957178 | 1.038328 | 0.024383 | 0.919135 | 0.106796 | 0.096326 | 0.009709 | 0.009709 | 2251.515 | 131.5694 | 17 |
| LQP | 4.5 | 0.949 | 0.946819 | 1.082751 | 0.028398 | 0.918226 | 0.106796 | 0.096326 | 0.009709 | 0.009709 | 2279.682 | 159.736 | 9 |
| LQT | 4.5 | 0.9595 | 0.953956 | 1.182973 | 0.027831 | 1.049067 | 0.126214 | 0.111365 | 0.009709 | 0.009709 | 2226.416 | 106.4697 | 10 |
| LHP | 4.5 | 0.9688 | 0.966978 | 0.541385 | 0.01816 | 0.477047 | 0.106796 | 0.096326 | 0.009709 | 0.009709 | 2226.429 | 106.4828 | 26 |
| LHT | 4.5 | 0.9645 | 0.955227 | 1.38589 | 0.029369 | 1.267961 | 0.116505 | 0.103941 | 0.009709 | 0.009709 | 2639.291 | 519.3451 | 70 |
| LPT | 4.5 | 0.9716 | 0.95897 | 1.104876 | 0.027029 | 1.023775 | 0.145631 | 0.125642 | 0.009709 | 0.009709 | 2179.954 | 60.0081 | 15 |
| QHP | 4.5 | 0.9536 | 0.952291 | 1.189385 | 0.027303 | 1.036345 | 0.116505 | 0.103941 | 0.009709 | 0.009709 | 2253.682 | 133.7364 | 17 |
| QHT | 4.5 | 0.9577 | 0.952391 | 1.299757 | 0.028849 | 1.150205 | 0.116505 | 0.103941 | 0.009709 | 0.009709 | 2224.813 | 104.8673 | 14 |
| QPT | 4.5 | 0.957 | 0.950672 | 1.296474 | 0.029695 | 1.139983 | 0.135922 | 0.118599 | 0.009709 | 0.009709 | 2220.081 | 100.1354 | 12 |
| HPT | 4.5 | 0.9619 | 0.952994 | 1.31722 | 0.02943 | 1.190732 | 0.116505 | 0.103941 | 0.009709 | 0.009709 | 2598.879 | 478.9327 | 68 |
| LQHP | 4.5 | 0.9691 | 0.966076 | 0.799721 | 0.01938 | 0.732209 | 0.106796 | 0.096326 | 0.009709 | 0.009709 | 2188.942 | 68.99567 | 19 |
| LQHT | 4.5 | 0.9592 | 0.954258 | 1.210285 | 0.027584 | 1.076023 | 0.116505 | 0.103941 | 0.009709 | 0.009709 | 2233.587 | 113.6409 | 14 |
| LQPT | 4.5 | 0.9703 | 0.962576 | 0.98433 | 0.023415 | 0.904527 | 0.126214 | 0.111365 | 0.009709 | 0.009709 | 2155.997 | 36.05154 | 11 |
| LHPT | 4.5 | 0.9719 | 0.962208 | 0.914874 | 0.023693 | 0.844437 | 0.126214 | 0.111365 | 0.009709 | 0.009709 | 2187.435 | 67.48875 | 21 |
| QHPT | 4.5 | 0.9577 | 0.951747 | 1.284762 | 0.029125 | 1.134046 | 0.135922 | 0.118599 | 0.009709 | 0.009709 | 2238.357 | 118.4111 | 21 |
| LQHPT | 4.5 | 0.9711 | 0.964551 | 0.932503 | 0.021831 | 0.861556 | 0.135922 | 0.118599 | 0.009709 | 0.009709 | 2168.055 | 48.10899 | 18 |
| L | 5 | 0.8065 | 0.80097 | 1.823453 | 0.06602 | 0.659959 | 0.106796 | 0.096326 | 0.009709 | 0.009709 | 2559.957 | 440.0115 | 3 |
| Q | 5 | 0.8963 | 0.895259 | 2.272304 | 0.050868 | 1.622687 | 0.106796 | 0.096326 | 0.009709 | 0.009709 | 2436.736 | 316.7906 | 3 |
| H | 5 | 0.9611 | 0.959531 | 0.911061 | 0.024277 | 0.793917 | 0.106796 | 0.096326 | 0.009709 | 0.009709 | 3137.193 | 1017.247 | 83 |
| P | 5 | 0.7551 | 0.754488 | 6.118659 | 0.117819 | 2.245263 | 0.106796 | 0.096326 | 0.009709 | 0.009709 | 2553.141 | 433.1956 | 3 |
| T | 5 | 0.9671 | 0.938017 | 1.398498 | 0.034111 | 1.358664 | 0.165049 | 0.139159 | 0.009709 | 0.009709 | 2321.814 | 201.868 | 14 |
| LQ | 5 | 0.9457 | 0.944592 | 1.160814 | 0.02832 | 0.987443 | 0.106796 | 0.096326 | 0.009709 | 0.009709 | 2312.683 | 192.7375 | 6 |
| LH | 5 | 0.9605 | 0.95818 | 0.908617 | 0.024847 | 0.785586 | 0.106796 | 0.096326 | 0.009709 | 0.009709 | 2653.643 | 533.6967 | 68 |
| LP | 5 | 0.8868 | 0.884751 | 1.270451 | 0.051439 | 0.546689 | 0.116505 | 0.103941 | 0.009709 | 0.009709 | 2444.98 | 325.0341 | 6 |
| LT | 5 | 0.9614 | 0.943033 | 1.176565 | 0.031894 | 1.083717 | 0.15534 | 0.132496 | 0.009709 | 0.009709 | 2325.073 | 205.1271 | 15 |
| QH | 5 | 0.9545 | 0.953366 | 1.162057 | 0.026287 | 1.017015 | 0.106796 | 0.096326 | 0.009709 | 0.009709 | 2257.383 | 137.4366 | 14 |
| QP | 5 | 0.9104 | 0.907951 | 2.333106 | 0.049999 | 1.798292 | 0.106796 | 0.096326 | 0.009709 | 0.009709 | 2412.559 | 292.6131 | 6 |
| QT | 5 | 0.957 | 0.951186 | 1.298915 | 0.029413 | 1.143642 | 0.116505 | 0.103941 | 0.009709 | 0.009709 | 2223.125 | 103.1794 | 9 |
| HP | 5 | 0.9595 | 0.957461 | 0.83898 | 0.023692 | 0.723645 | 0.106796 | 0.096326 | 0.009709 | 0.009709 | 2975.417 | 855.4714 | 80 |
| HT | 5 | 0.9615 | 0.953435 | 1.400288 | 0.029998 | 1.266308 | 0.116505 | 0.103941 | 0.009709 | 0.009709 | 2547.819 | 427.8728 | 63 |
| PT | 5 | 0.9611 | 0.944564 | 1.288854 | 0.030306 | 1.193854 | 0.165049 | 0.139159 | 0.009709 | 0.009709 | 2282.105 | 162.1592 | 15 |
| LQH | 5 | 0.957 | 0.95579 | 1.050994 | 0.024792 | 0.926461 | 0.106796 | 0.096326 | 0.009709 | 0.009709 | 2256.56 | 136.6138 | 14 |
| LQP | 5 | 0.9481 | 0.946035 | 1.100305 | 0.02888 | 0.929137 | 0.106796 | 0.096326 | 0.009709 | 0.009709 | 2283.828 | 163.8817 | 9 |
| LQT | 5 | 0.9592 | 0.953942 | 1.179176 | 0.027595 | 1.0458 | 0.126214 | 0.111365 | 0.009709 | 0.009709 | 2230.578 | 110.6323 | 9 |
| LHP | 5 | 0.9683 | 0.966538 | 0.549073 | 0.018279 | 0.483512 | 0.106796 | 0.096326 | 0.009709 | 0.009709 | 2230.093 | 110.147 | 24 |
| LHT | 5 | 0.9614 | 0.953375 | 1.401618 | 0.030042 | 1.267781 | 0.116505 | 0.103941 | 0.009709 | 0.009709 | 2576.912 | 456.9656 | 65 |
| LPT | 5 | 0.971 | 0.958666 | 1.115202 | 0.026963 | 1.033337 | 0.145631 | 0.125642 | 0.009709 | 0.009709 | 2191.257 | 71.31093 | 15 |
| QHP | 5 | 0.9529 | 0.951489 | 1.205859 | 0.027705 | 1.048209 | 0.116505 | 0.103941 | 0.009709 | 0.009709 | 2257.06 | 137.1141 | 16 |
| QHT | 5 | 0.9571 | 0.951811 | 1.309113 | 0.029073 | 1.156641 | 0.116505 | 0.103941 | 0.009709 | 0.009709 | 2233.037 | 113.0912 | 15 |
| QPT | 5 | 0.9563 | 0.950149 | 1.308662 | 0.029868 | 1.148132 | 0.126214 | 0.111365 | 0.009709 | 0.009709 | 2227.739 | 107.7926 | 12 |
| HPT | 5 | 0.9611 | 0.952869 | 1.327081 | 0.029212 | 1.198322 | 0.116505 | 0.103941 | 0.009709 | 0.009709 | 4619.723 | 2499.777 | 94 |
| LQHP | 5 | 0.9669 | 0.964189 | 0.820292 | 0.0203 | 0.743947 | 0.106796 | 0.096326 | 0.009709 | 0.009709 | 2194.287 | 74.34073 | 16 |
| LQHT | 5 | 0.959 | 0.954214 | 1.204692 | 0.02745 | 1.069647 | 0.126214 | 0.111365 | 0.009709 | 0.009709 | 2238.622 | 118.6759 | 14 |
| LQPT | 5 | 0.9687 | 0.961743 | 1.0032 | 0.02368 | 0.917859 | 0.135922 | 0.118599 | 0.009709 | 0.009709 | 2166.704 | 46.75784 | 11 |
| LHPT | 5 | 0.971 | 0.962282 | 0.907499 | 0.023414 | 0.835752 | 0.116505 | 0.103941 | 0.009709 | 0.009709 | 2189.99 | 70.04451 | 18 |
| QHPT | 5 | 0.9569 | 0.951138 | 1.295066 | 0.029357 | 1.140169 | 0.116505 | 0.103941 | 0.009709 | 0.009709 | 2235.323 | 115.3769 | 17 |
| LQHPT | 5 | 0.9694 | 0.963037 | 0.971335 | 0.022572 | 0.892207 | 0.126214 | 0.111365 | 0.009709 | 0.009709 | 2164.958 | 45.01232 | 12 |
| L | 5.5 | 0.8063 | 0.801082 | 1.855697 | 0.066596 | 0.670941 | 0.106796 | 0.096326 | 0.009709 | 0.009709 | 2560.027 | 440.0816 | 3 |
| Q | 5.5 | 0.8963 | 0.895232 | 2.271118 | 0.050855 | 1.621643 | 0.106796 | 0.096326 | 0.009709 | 0.009709 | 2436.818 | 316.8725 | 3 |
| H | 5.5 | 0.9608 | 0.95888 | 0.921759 | 0.024577 | 0.801781 | 0.106796 | 0.096326 | 0.009709 | 0.009709 | 3214.151 | 1094.205 | 84 |
| P | 5.5 | 0.7552 | 0.754567 | 6.112925 | 0.117749 | 2.243473 | 0.106796 | 0.096326 | 0.009709 | 0.009709 | 2553.166 | 433.2202 | 3 |
| T | 5.5 | 0.9555 | 0.924279 | 1.40646 | 0.04174 | 1.256774 | 0.145631 | 0.125642 | 0.009709 | 0.009709 | 2366.975 | 247.0296 | 12 |
| LQ | 5.5 | 0.9457 | 0.944553 | 1.163943 | 0.028385 | 0.989866 | 0.106796 | 0.096326 | 0.009709 | 0.009709 | 2313.156 | 193.2096 | 6 |
| LH | 5.5 | 0.9581 | 0.955096 | 0.968138 | 0.026331 | 0.828818 | 0.106796 | 0.096326 | 0.009709 | 0.009709 | 2571.649 | 451.7033 | 61 |
| LP | 5.5 | 0.8864 | 0.884293 | 1.283271 | 0.051806 | 0.549751 | 0.116505 | 0.103941 | 0.009709 | 0.009709 | 2445.331 | 325.3852 | 6 |
| LT | 5.5 | 0.9455 | 0.925552 | 1.108646 | 0.033712 | 0.986772 | 0.15534 | 0.132496 | 0.009709 | 0.009709 | 2363.59 | 243.6439 | 13 |
| QH | 5.5 | 0.9539 | 0.95272 | 1.17629 | 0.026551 | 1.027361 | 0.106796 | 0.096326 | 0.009709 | 0.009709 | 2268.34 | 148.394 | 16 |
| QP | 5.5 | 0.9103 | 0.907871 | 2.329271 | 0.049918 | 1.796464 | 0.106796 | 0.096326 | 0.009709 | 0.009709 | 2412.696 | 292.7498 | 6 |
| QT | 5.5 | 0.9564 | 0.950572 | 1.305866 | 0.029684 | 1.146741 | 0.116505 | 0.103941 | 0.009709 | 0.009709 | 2229.072 | 109.1263 | 9 |
| HP | 5.5 | 0.9586 | 0.956569 | 0.867926 | 0.024084 | 0.74958 | 0.106796 | 0.096326 | 0.009709 | 0.009709 | 2600.26 | 480.3141 | 65 |
| HT | 5.5 | 0.9611 | 0.952994 | 1.398273 | 0.03018 | 1.260196 | 0.116505 | 0.103941 | 0.009709 | 0.009709 | 2562.141 | 442.1956 | 63 |
| PT | 5.5 | 0.9502 | 0.93243 | 1.320518 | 0.036508 | 1.141139 | 0.145631 | 0.125642 | 0.009709 | 0.009709 | 2317.593 | 197.6469 | 13 |
| LQH | 5.5 | 0.9566 | 0.955361 | 1.058486 | 0.024961 | 0.931904 | 0.106796 | 0.096326 | 0.009709 | 0.009709 | 2264.68 | 144.7341 | 15 |
| LQP | 5.5 | 0.9472 | 0.945275 | 1.119639 | 0.029335 | 0.941357 | 0.106796 | 0.096326 | 0.009709 | 0.009709 | 2287.618 | 167.6718 | 9 |
| LQT | 5.5 | 0.959 | 0.953752 | 1.17591 | 0.027459 | 1.042666 | 0.116505 | 0.103941 | 0.009709 | 0.009709 | 2237.25 | 117.3038 | 9 |
| LHP | 5.5 | 0.9678 | 0.966045 | 0.559978 | 0.018393 | 0.493601 | 0.106796 | 0.096326 | 0.009709 | 0.009709 | 2234.436 | 114.4905 | 22 |
| LHT | 5.5 | 0.9599 | 0.951292 | 1.438258 | 0.031166 | 1.290668 | 0.106796 | 0.096326 | 0.009709 | 0.009709 | 2597.365 | 477.4187 | 65 |
| LPT | 5.5 | 0.9702 | 0.95833 | 1.122908 | 0.026894 | 1.040273 | 0.145631 | 0.125642 | 0.009709 | 0.009709 | 2200.633 | 80.68736 | 14 |
| QHP | 5.5 | 0.9521 | 0.95069 | 1.219511 | 0.028062 | 1.057672 | 0.106796 | 0.096326 | 0.009709 | 0.009709 | 2257.796 | 137.8503 | 14 |
| QHT | 5.5 | 0.9567 | 0.951332 | 1.315522 | 0.02925 | 1.160344 | 0.116505 | 0.103941 | 0.009709 | 0.009709 | 2238.895 | 118.949 | 15 |
| QPT | 5.5 | 0.9556 | 0.949621 | 1.325584 | 0.030037 | 1.160429 | 0.116505 | 0.103941 | 0.009709 | 0.009709 | 2231.584 | 111.638 | 11 |
| HPT | 5.5 | 0.9609 | 0.952775 | 1.326666 | 0.028962 | 1.198506 | 0.116505 | 0.103941 | 0.009709 | 0.009709 | 2811.715 | 691.7693 | 76 |
| LQHP | 5.5 | 0.9649 | 0.96214 | 0.888254 | 0.021383 | 0.80161 | 0.106796 | 0.096326 | 0.009709 | 0.009709 | 2213.378 | 93.43214 | 18 |
| LQHT | 5.5 | 0.9586 | 0.953971 | 1.202185 | 0.027355 | 1.066936 | 0.106796 | 0.096326 | 0.009709 | 0.009709 | 2235.475 | 115.5295 | 11 |
| LQPT | 5.5 | 0.9671 | 0.960919 | 1.019727 | 0.023902 | 0.929349 | 0.126214 | 0.111365 | 0.009709 | 0.009709 | 2174.086 | 54.14032 | 10 |
| LHPT | 5.5 | 0.9704 | 0.9622 | 0.907683 | 0.023259 | 0.834994 | 0.116505 | 0.103941 | 0.009709 | 0.009709 | 2197.419 | 77.4732 | 17 |
| QHPT | 5.5 | 0.9562 | 0.950592 | 1.310593 | 0.029601 | 1.150476 | 0.106796 | 0.096326 | 0.009709 | 0.009709 | 2242.022 | 122.0758 | 17 |
| LQHPT | 5.5 | 0.9672 | 0.961597 | 1.012493 | 0.023395 | 0.924659 | 0.126214 | 0.111365 | 0.009709 | 0.009709 | 2178.804 | 58.85839 | 12 |
| L | 6 | 0.8062 | 0.801056 | 1.889304 | 0.067228 | 0.683147 | 0.116505 | 0.103941 | 0.009709 | 0.009709 | 2560.106 | 440.1596 | 3 |
| Q | 6 | 0.8963 | 0.895212 | 2.270189 | 0.050843 | 1.620714 | 0.106796 | 0.096326 | 0.009709 | 0.009709 | 2436.907 | 316.9615 | 3 |
| H | 6 | 0.9605 | 0.958295 | 0.932866 | 0.024954 | 0.808874 | 0.106796 | 0.096326 | 0.009709 | 0.009709 | 2740.814 | 620.8682 | 71 |
| P | 6 | 0.7552 | 0.754637 | 6.107628 | 0.117694 | 2.24186 | 0.106796 | 0.096326 | 0.009709 | 0.009709 | 2553.192 | 433.2466 | 3 |
| T | 6 | 0.9387 | 0.922946 | 1.373079 | 0.040441 | 1.131353 | 0.135922 | 0.118599 | 0.009709 | 0.009709 | 2380.639 | 260.6934 | 10 |
| LQ | 6 | 0.9456 | 0.944474 | 1.166952 | 0.028465 | 0.992072 | 0.106796 | 0.096326 | 0.009709 | 0.009709 | 2313.64 | 193.6943 | 6 |
| LH | 6 | 0.9547 | 0.950594 | 1.035596 | 0.028374 | 0.875464 | 0.106796 | 0.096326 | 0.009709 | 0.009709 | 2636.927 | 516.9816 | 64 |
| LP | 6 | 0.886 | 0.883789 | 1.298737 | 0.052229 | 0.553631 | 0.116505 | 0.103941 | 0.009709 | 0.009709 | 2445.716 | 325.7702 | 6 |
| LT | 6 | 0.9237 | 0.908135 | 1.136394 | 0.038439 | 0.899831 | 0.135922 | 0.118599 | 0.009709 | 0.009709 | 2396.671 | 276.7248 | 11 |
| QH | 6 | 0.9533 | 0.952113 | 1.192557 | 0.026817 | 1.039149 | 0.106796 | 0.096326 | 0.009709 | 0.009709 | 2268.217 | 148.2713 | 14 |
| QP | 6 | 0.9103 | 0.907784 | 2.326364 | 0.049867 | 1.795014 | 0.106796 | 0.096326 | 0.009709 | 0.009709 | 2412.845 | 292.8988 | 6 |
| QT | 6 | 0.9558 | 0.950013 | 1.311568 | 0.029848 | 1.149061 | 0.116505 | 0.103941 | 0.009709 | 0.009709 | 2235.41 | 115.464 | 9 |
| HP | 6 | 0.9578 | 0.955232 | 0.891588 | 0.024539 | 0.770995 | 0.106796 | 0.096326 | 0.009709 | 0.009709 | 2597.31 | 477.3643 | 64 |
| HT | 6 | 0.9605 | 0.952063 | 1.433798 | 0.030735 | 1.290792 | 0.106796 | 0.096326 | 0.009709 | 0.009709 | 2675.549 | 555.6034 | 69 |
| PT | 6 | 0.9401 | 0.927327 | 1.317094 | 0.038505 | 1.066055 | 0.145631 | 0.125642 | 0.009709 | 0.009709 | 2338.402 | 218.4557 | 10 |
| LQH | 6 | 0.9562 | 0.955016 | 1.060679 | 0.025048 | 0.932576 | 0.106796 | 0.096326 | 0.009709 | 0.009709 | 2264.653 | 144.7068 | 13 |
| LQP | 6 | 0.9463 | 0.944579 | 1.14212 | 0.029773 | 0.95796 | 0.106796 | 0.096326 | 0.009709 | 0.009709 | 2288.627 | 168.6813 | 8 |
| LQT | 6 | 0.9587 | 0.953579 | 1.171423 | 0.027273 | 1.03792 | 0.126214 | 0.111365 | 0.009709 | 0.009709 | 2244.477 | 124.5316 | 9 |
| LHP | 6 | 0.9672 | 0.965365 | 0.573474 | 0.018557 | 0.506437 | 0.106796 | 0.096326 | 0.009709 | 0.009709 | 2252.56 | 132.614 | 24 |
| LHT | 6 | 0.9574 | 0.948722 | 1.453072 | 0.032205 | 1.287962 | 0.106796 | 0.096326 | 0.009709 | 0.009709 | 2530.839 | 410.893 | 58 |
| LPT | 6 | 0.9699 | 0.958034 | 1.118606 | 0.026783 | 1.036036 | 0.145631 | 0.125642 | 0.009709 | 0.009709 | 2207.68 | 87.73375 | 13 |
| QHP | 6 | 0.9514 | 0.949837 | 1.233967 | 0.028443 | 1.067199 | 0.106796 | 0.096326 | 0.009709 | 0.009709 | 2269.753 | 149.8067 | 16 |
| QHT | 6 | 0.9562 | 0.950779 | 1.317197 | 0.029455 | 1.159208 | 0.116505 | 0.103941 | 0.009709 | 0.009709 | 2247.497 | 127.551 | 16 |
| QPT | 6 | 0.9548 | 0.948948 | 1.3419 | 0.030307 | 1.172418 | 0.116505 | 0.103941 | 0.009709 | 0.009709 | 2238.835 | 118.8893 | 11 |
| HPT | 6 | 0.9604 | 0.952504 | 1.328172 | 0.028717 | 1.200346 | 0.116505 | 0.103941 | 0.009709 | 0.009709 | 2632.492 | 512.5457 | 68 |
| LQHP | 6 | 0.9628 | 0.960083 | 0.918628 | 0.022384 | 0.823416 | 0.106796 | 0.096326 | 0.009709 | 0.009709 | 2227.115 | 107.1688 | 18 |
| LQHT | 6 | 0.9581 | 0.95367 | 1.195502 | 0.027248 | 1.059769 | 0.106796 | 0.096326 | 0.009709 | 0.009709 | 2245.675 | 125.7293 | 13 |
| LQPT | 6 | 0.9658 | 0.95991 | 1.036059 | 0.024268 | 0.940198 | 0.126214 | 0.111365 | 0.009709 | 0.009709 | 2183.359 | 63.41346 | 10 |
| LHPT | 6 | 0.9698 | 0.961887 | 0.913795 | 0.023301 | 0.840003 | 0.116505 | 0.103941 | 0.009709 | 0.009709 | 2216.429 | 96.48331 | 20 |
| QHPT | 6 | 0.9553 | 0.949921 | 1.325452 | 0.029844 | 1.16048 | 0.106796 | 0.096326 | 0.009709 | 0.009709 | 2243.097 | 123.1511 | 15 |
| LQHPT | 6 | 0.9661 | 0.960444 | 1.033951 | 0.023939 | 0.940381 | 0.126214 | 0.111365 | 0.009709 | 0.009709 | 2184.371 | 64.42517 | 11 |
| L | 6.5 | 0.8061 | 0.800944 | 1.920391 | 0.067828 | 0.694896 | 0.106796 | 0.096326 | 0.009709 | 0.009709 | 2560.191 | 440.2456 | 3 |
| Q | 6.5 | 0.8962 | 0.89521 | 2.268976 | 0.050819 | 1.619514 | 0.106796 | 0.096326 | 0.009709 | 0.009709 | 2437.003 | 317.0575 | 3 |
| H | 6.5 | 0.9599 | 0.957495 | 0.939641 | 0.025459 | 0.810549 | 0.106796 | 0.096326 | 0.009709 | 0.009709 | 2500.159 | 380.2134 | 52 |
| P | 6.5 | 0.7553 | 0.754704 | 6.100846 | 0.117635 | 2.239545 | 0.106796 | 0.096326 | 0.009709 | 0.009709 | 2553.223 | 433.2768 | 3 |
| T | 6.5 | 0.9393 | 0.925428 | 1.118788 | 0.03759 | 0.887787 | 0.145631 | 0.125642 | 0.009709 | 0.009709 | 2396.781 | 276.8349 | 10 |
| LQ | 6.5 | 0.9456 | 0.944396 | 1.169387 | 0.028532 | 0.993874 | 0.106796 | 0.096326 | 0.009709 | 0.009709 | 2314.16 | 194.2142 | 6 |
| LH | 6.5 | 0.9504 | 0.946698 | 1.056454 | 0.029505 | 0.883261 | 0.116505 | 0.103941 | 0.009709 | 0.009709 | 2497.978 | 378.0321 | 48 |
| LP | 6.5 | 0.8855 | 0.883286 | 1.314177 | 0.052628 | 0.557235 | 0.116505 | 0.103941 | 0.009709 | 0.009709 | 2446.139 | 326.1934 | 6 |
| LT | 6.5 | 0.9213 | 0.90621 | 1.134849 | 0.040007 | 0.869196 | 0.135922 | 0.118599 | 0.009709 | 0.009709 | 2404.145 | 284.1994 | 11 |
| QH | 6.5 | 0.9527 | 0.95135 | 1.210369 | 0.027204 | 1.05187 | 0.106796 | 0.096326 | 0.009709 | 0.009709 | 2268.181 | 148.2352 | 12 |
| QP | 6.5 | 0.9102 | 0.907715 | 2.321802 | 0.049771 | 1.792221 | 0.106796 | 0.096326 | 0.009709 | 0.009709 | 2413.018 | 293.0723 | 6 |
| QT | 6.5 | 0.9552 | 0.949336 | 1.317933 | 0.030045 | 1.151752 | 0.116505 | 0.103941 | 0.009709 | 0.009709 | 2239.196 | 119.2501 | 8 |
| HP | 6.5 | 0.9564 | 0.953559 | 0.916213 | 0.025019 | 0.793207 | 0.106796 | 0.096326 | 0.009709 | 0.009709 | 2389.765 | 269.8191 | 39 |
| HT | 6.5 | 0.96 | 0.95157 | 1.433333 | 0.030895 | 1.285595 | 0.106796 | 0.096326 | 0.009709 | 0.009709 | 2389.36 | 269.4139 | 39 |
| PT | 6.5 | 0.9388 | 0.92589 | 1.308194 | 0.039267 | 1.042765 | 0.145631 | 0.125642 | 0.009709 | 0.009709 | 2345.166 | 225.2203 | 10 |
| LQH | 6.5 | 0.9559 | 0.954607 | 1.0662 | 0.025145 | 0.936341 | 0.106796 | 0.096326 | 0.009709 | 0.009709 | 2267.34 | 147.3942 | 12 |
| LQP | 6.5 | 0.9458 | 0.944002 | 1.150768 | 0.030067 | 0.96118 | 0.106796 | 0.096326 | 0.009709 | 0.009709 | 2291.35 | 171.4041 | 8 |
| LQT | 6.5 | 0.9582 | 0.953237 | 1.16766 | 0.027089 | 1.03343 | 0.126214 | 0.111365 | 0.009709 | 0.009709 | 2248.81 | 128.8642 | 8 |
| LHP | 6.5 | 0.9665 | 0.964458 | 0.581146 | 0.018732 | 0.513718 | 0.106796 | 0.096326 | 0.009709 | 0.009709 | 2267.68 | 147.7343 | 25 |
| LHT | 6.5 | 0.9539 | 0.945121 | 1.43266 | 0.033641 | 1.241822 | 0.106796 | 0.096326 | 0.009709 | 0.009709 | 2490.094 | 370.1481 | 51 |
| LPT | 6.5 | 0.9694 | 0.957725 | 1.111358 | 0.02668 | 1.028758 | 0.135922 | 0.118599 | 0.009709 | 0.009709 | 2218.112 | 98.16639 | 13 |
| QHP | 6.5 | 0.9506 | 0.949126 | 1.250877 | 0.028786 | 1.078388 | 0.106796 | 0.096326 | 0.009709 | 0.009709 | 2273.402 | 153.4564 | 15 |
| QHT | 6.5 | 0.9557 | 0.950288 | 1.319129 | 0.029613 | 1.157601 | 0.116505 | 0.103941 | 0.009709 | 0.009709 | 2250.845 | 130.8988 | 15 |
| QPT | 6.5 | 0.954 | 0.948711 | 1.355545 | 0.030296 | 1.183291 | 0.106796 | 0.096326 | 0.009709 | 0.009709 | 2242.647 | 122.7009 | 10 |
| HPT | 6.5 | 0.9598 | 0.951662 | 1.33631 | 0.028795 | 1.20914 | 0.116505 | 0.103941 | 0.009709 | 0.009709 | 2980.804 | 860.858 | 80 |
| LQHP | 6.5 | 0.9607 | 0.957898 | 0.974195 | 0.023492 | 0.869232 | 0.106796 | 0.096326 | 0.009709 | 0.009709 | 2230.768 | 110.822 | 14 |
| LQHT | 6.5 | 0.9577 | 0.953324 | 1.190084 | 0.027148 | 1.053497 | 0.116505 | 0.103941 | 0.009709 | 0.009709 | 2248.436 | 128.4903 | 12 |
| LQPT | 6.5 | 0.9644 | 0.958784 | 1.056001 | 0.024784 | 0.953781 | 0.126214 | 0.111365 | 0.009709 | 0.009709 | 2193.497 | 73.5514 | 10 |
| LHPT | 6.5 | 0.9692 | 0.961405 | 0.92258 | 0.023477 | 0.847551 | 0.116505 | 0.103941 | 0.009709 | 0.009709 | 2227.093 | 107.1474 | 20 |
| QHPT | 6.5 | 0.9543 | 0.949141 | 1.343697 | 0.030157 | 1.174061 | 0.106796 | 0.096326 | 0.009709 | 0.009709 | 2244.273 | 124.3272 | 13 |
| LQHPT | 6.5 | 0.9648 | 0.959281 | 1.053594 | 0.024533 | 0.953479 | 0.126214 | 0.111365 | 0.009709 | 0.009709 | 2195.848 | 75.90197 | 12 |
| L | 7 | 0.8059 | 0.800839 | 1.952484 | 0.068435 | 0.706908 | 0.106796 | 0.096326 | 0.009709 | 0.009709 | 2560.285 | 440.3393 | 3 |
| Q | 7 | 0.8962 | 0.895175 | 2.267835 | 0.050809 | 1.618497 | 0.106796 | 0.096326 | 0.009709 | 0.009709 | 2437.106 | 317.1603 | 3 |
| H | 7 | 0.9592 | 0.956739 | 0.939204 | 0.025793 | 0.804024 | 0.106796 | 0.096326 | 0.009709 | 0.009709 | 2555.403 | 435.4573 | 56 |
| P | 7 | 0.7554 | 0.754763 | 6.093797 | 0.117579 | 2.236999 | 0.106796 | 0.096326 | 0.009709 | 0.009709 | 2553.258 | 433.3125 | 3 |
| T | 7 | 0.9185 | 0.901067 | 0.870056 | 0.033059 | 0.822143 | 0.126214 | 0.111365 | 0.009709 | 0.009709 | 2405.104 | 285.1581 | 8 |
| LQ | 7 | 0.9455 | 0.9443 | 1.171878 | 0.028589 | 0.995888 | 0.106796 | 0.096326 | 0.009709 | 0.009709 | 2314.704 | 194.758 | 6 |
| LH | 7 | 0.949 | 0.943232 | 1.037499 | 0.029741 | 0.87508 | 0.116505 | 0.103941 | 0.009709 | 0.009709 | 2513.112 | 393.1662 | 48 |
| LP | 7 | 0.885 | 0.882697 | 1.330793 | 0.053071 | 0.561239 | 0.106796 | 0.096326 | 0.009709 | 0.009709 | 2446.607 | 326.6609 | 6 |
| LT | 7 | 0.9188 | 0.904146 | 1.134018 | 0.041435 | 0.839201 | 0.135922 | 0.118599 | 0.009709 | 0.009709 | 2411.776 | 291.8298 | 11 |
| QH | 7 | 0.952 | 0.950571 | 1.224986 | 0.027582 | 1.061067 | 0.106796 | 0.096326 | 0.009709 | 0.009709 | 2273.707 | 153.7614 | 12 |
| QP | 7 | 0.9101 | 0.907608 | 2.320049 | 0.049718 | 1.791336 | 0.106796 | 0.096326 | 0.009709 | 0.009709 | 2413.205 | 293.2594 | 6 |
| QT | 7 | 0.9545 | 0.948673 | 1.325784 | 0.030331 | 1.154589 | 0.116505 | 0.103941 | 0.009709 | 0.009709 | 2245.547 | 125.6013 | 8 |
| HP | 7 | 0.9546 | 0.95148 | 0.916339 | 0.025116 | 0.793087 | 0.106796 | 0.096326 | 0.009709 | 0.009709 | 2425.699 | 305.7534 | 43 |
| HT | 7 | 0.9591 | 0.950457 | 1.43633 | 0.031506 | 1.278928 | 0.106796 | 0.096326 | 0.009709 | 0.009709 | 2424.639 | 304.6929 | 42 |
| PT | 7 | 0.937 | 0.923792 | 1.33082 | 0.04045 | 1.045585 | 0.145631 | 0.125642 | 0.009709 | 0.009709 | 2349.216 | 229.2701 | 9 |
| LQH | 7 | 0.9555 | 0.954247 | 1.068647 | 0.025201 | 0.937414 | 0.116505 | 0.103941 | 0.009709 | 0.009709 | 2278.276 | 158.3301 | 14 |
| LQP | 7 | 0.9452 | 0.943467 | 1.165794 | 0.030315 | 0.972682 | 0.106796 | 0.096326 | 0.009709 | 0.009709 | 2293.83 | 173.8843 | 8 |
| LQT | 7 | 0.9573 | 0.952466 | 1.159295 | 0.027044 | 1.022057 | 0.116505 | 0.103941 | 0.009709 | 0.009709 | 2259.521 | 139.5748 | 9 |
| LHP | 7 | 0.9657 | 0.963774 | 0.596613 | 0.018869 | 0.528026 | 0.106796 | 0.096326 | 0.009709 | 0.009709 | 2269.274 | 149.3284 | 22 |
| LHT | 7 | 0.9525 | 0.944034 | 1.419479 | 0.034098 | 1.224728 | 0.116505 | 0.103941 | 0.009709 | 0.009709 | 2423.084 | 303.1382 | 38 |
| LPT | 7 | 0.9689 | 0.957367 | 1.105592 | 0.026615 | 1.022649 | 0.145631 | 0.125642 | 0.009709 | 0.009709 | 2225.669 | 105.7234 | 12 |
| QHP | 7 | 0.9498 | 0.948297 | 1.266326 | 0.029114 | 1.089233 | 0.106796 | 0.096326 | 0.009709 | 0.009709 | 2279.485 | 159.5392 | 15 |
| QHT | 7 | 0.9549 | 0.949666 | 1.323982 | 0.029868 | 1.158183 | 0.106796 | 0.096326 | 0.009709 | 0.009709 | 2253.843 | 133.8971 | 14 |
| QPT | 7 | 0.9536 | 0.948456 | 1.371118 | 0.030435 | 1.196158 | 0.106796 | 0.096326 | 0.009709 | 0.009709 | 2247.756 | 127.8097 | 10 |
| HPT | 7 | 0.9591 | 0.950373 | 1.339485 | 0.028992 | 1.213243 | 0.116505 | 0.103941 | 0.009709 | 0.009709 | 2493.77 | 373.8237 | 56 |
| LQHP | 7 | 0.9588 | 0.956142 | 1.022458 | 0.024381 | 0.907464 | 0.106796 | 0.096326 | 0.009709 | 0.009709 | 2241.824 | 121.8779 | 14 |
| LQHT | 7 | 0.9572 | 0.95284 | 1.191883 | 0.027156 | 1.052749 | 0.116505 | 0.103941 | 0.009709 | 0.009709 | 2253.905 | 133.959 | 12 |
| LQPT | 7 | 0.9627 | 0.957619 | 1.072336 | 0.025256 | 0.963145 | 0.126214 | 0.111365 | 0.009709 | 0.009709 | 2204.264 | 84.3178 | 10 |
| LHPT | 7 | 0.9686 | 0.961104 | 0.918936 | 0.023505 | 0.843042 | 0.116505 | 0.103941 | 0.009709 | 0.009709 | 2231.136 | 111.1898 | 18 |
| QHPT | 7 | 0.9533 | 0.948666 | 1.351496 | 0.030261 | 1.178789 | 0.106796 | 0.096326 | 0.009709 | 0.009709 | 2245.915 | 125.9694 | 11 |
| LQHPT | 7 | 0.9632 | 0.957964 | 1.070869 | 0.025107 | 0.963447 | 0.126214 | 0.111365 | 0.009709 | 0.009709 | 2208.691 | 88.74482 | 13 |
| L | 7.5 | 0.8058 | 0.800704 | 1.986149 | 0.069048 | 0.719329 | 0.106796 | 0.096326 | 0.009709 | 0.009709 | 2560.387 | 440.4408 | 3 |
| Q | 7.5 | 0.8962 | 0.895159 | 2.26599 | 0.050775 | 1.616912 | 0.106796 | 0.096326 | 0.009709 | 0.009709 | 2437.216 | 317.27 | 3 |
| H | 7.5 | 0.9582 | 0.955329 | 0.943453 | 0.026534 | 0.799038 | 0.106796 | 0.096326 | 0.009709 | 0.009709 | 2441.191 | 321.2448 | 37 |
| P | 7.5 | 0.7555 | 0.754833 | 6.0863 | 0.117517 | 2.233896 | 0.106796 | 0.096326 | 0.009709 | 0.009709 | 2553.297 | 433.3509 | 3 |
| T | 7.5 | 0.9124 | 0.900778 | 0.832827 | 0.023881 | 0.791847 | 0.116505 | 0.103941 | 0.009709 | 0.009709 | 2406.127 | 286.1815 | 6 |
| LQ | 7.5 | 0.9455 | 0.944228 | 1.173934 | 0.028645 | 0.997394 | 0.106796 | 0.096326 | 0.009709 | 0.009709 | 2315.259 | 195.3132 | 6 |
| LH | 7.5 | 0.9448 | 0.938868 | 1.001186 | 0.029287 | 0.845548 | 0.116505 | 0.103941 | 0.009709 | 0.009709 | 2551.312 | 431.3659 | 51 |
| LP | 7.5 | 0.8844 | 0.882138 | 1.346178 | 0.053458 | 0.564902 | 0.106796 | 0.096326 | 0.009709 | 0.009709 | 2447.115 | 327.169 | 6 |
| LT | 7.5 | 0.9163 | 0.902327 | 1.126464 | 0.042464 | 0.808073 | 0.135922 | 0.118599 | 0.009709 | 0.009709 | 2416.774 | 296.8286 | 10 |
| QH | 7.5 | 0.9511 | 0.949655 | 1.240309 | 0.028069 | 1.069827 | 0.106796 | 0.096326 | 0.009709 | 0.009709 | 2284.99 | 165.0444 | 14 |
| QP | 7.5 | 0.91 | 0.907499 | 2.317836 | 0.049658 | 1.790201 | 0.106796 | 0.096326 | 0.009709 | 0.009709 | 2413.397 | 293.451 | 6 |
| QT | 7.5 | 0.9536 | 0.947848 | 1.333805 | 0.03068 | 1.156414 | 0.116505 | 0.103941 | 0.009709 | 0.009709 | 2252.289 | 132.3431 | 8 |
| HP | 7.5 | 0.9523 | 0.948814 | 0.916039 | 0.025205 | 0.790321 | 0.106796 | 0.096326 | 0.009709 | 0.009709 | 2396.515 | 276.5693 | 35 |
| HT | 7.5 | 0.9574 | 0.948628 | 1.423655 | 0.03224 | 1.252688 | 0.106796 | 0.096326 | 0.009709 | 0.009709 | 2403.471 | 283.5253 | 34 |
| PT | 7.5 | 0.9346 | 0.921059 | 1.357743 | 0.041955 | 1.047989 | 0.145631 | 0.125642 | 0.009709 | 0.009709 | 2358.341 | 238.3954 | 10 |
| LQH | 7.5 | 0.9539 | 0.952228 | 1.09072 | 0.026014 | 0.949956 | 0.116505 | 0.103941 | 0.009709 | 0.009709 | 2291.896 | 171.9501 | 15 |
| LQP | 7.5 | 0.9448 | 0.942961 | 1.180586 | 0.030575 | 0.983328 | 0.106796 | 0.096326 | 0.009709 | 0.009709 | 2296.049 | 176.1028 | 8 |
| LQT | 7.5 | 0.9556 | 0.95153 | 1.148513 | 0.02698 | 1.006221 | 0.106796 | 0.096326 | 0.009709 | 0.009709 | 2264.887 | 144.9412 | 8 |
| LHP | 7.5 | 0.9649 | 0.962568 | 0.609651 | 0.019282 | 0.539267 | 0.106796 | 0.096326 | 0.009709 | 0.009709 | 2270.538 | 150.5925 | 19 |
| LHT | 7.5 | 0.9523 | 0.942749 | 1.4162 | 0.034516 | 1.220903 | 0.116505 | 0.103941 | 0.009709 | 0.009709 | 2466.156 | 346.2099 | 43 |
| LPT | 7.5 | 0.9684 | 0.956809 | 1.101303 | 0.026601 | 1.018258 | 0.126214 | 0.111365 | 0.009709 | 0.009709 | 2235.912 | 115.9658 | 12 |
| QHP | 7.5 | 0.9491 | 0.947884 | 1.27604 | 0.029232 | 1.095493 | 0.106796 | 0.096326 | 0.009709 | 0.009709 | 2279.87 | 159.9245 | 13 |
| QHT | 7.5 | 0.9542 | 0.949045 | 1.32445 | 0.03007 | 1.153777 | 0.106796 | 0.096326 | 0.009709 | 0.009709 | 2265.262 | 145.3161 | 16 |
| QPT | 7.5 | 0.9532 | 0.948225 | 1.384057 | 0.030597 | 1.206593 | 0.106796 | 0.096326 | 0.009709 | 0.009709 | 2249.967 | 130.0214 | 9 |
| HPT | 7.5 | 0.958 | 0.948745 | 1.337111 | 0.029207 | 1.211212 | 0.116505 | 0.103941 | 0.009709 | 0.009709 | 2479.053 | 359.107 | 53 |
| LQHP | 7.5 | 0.9571 | 0.954378 | 1.059814 | 0.025303 | 0.935184 | 0.116505 | 0.103941 | 0.009709 | 0.009709 | 2249.808 | 129.8619 | 13 |
| LQHT | 7.5 | 0.9558 | 0.951875 | 1.17931 | 0.0272 | 1.036164 | 0.116505 | 0.103941 | 0.009709 | 0.009709 | 2258.59 | 138.6442 | 10 |
| LQPT | 7.5 | 0.9612 | 0.956593 | 1.081513 | 0.025602 | 0.966586 | 0.116505 | 0.103941 | 0.009709 | 0.009709 | 2210.775 | 90.82865 | 9 |
| LHPT | 7.5 | 0.9683 | 0.960699 | 0.926271 | 0.023624 | 0.849635 | 0.116505 | 0.103941 | 0.009709 | 0.009709 | 2231.031 | 111.0849 | 15 |
| QHPT | 7.5 | 0.953 | 0.948466 | 1.361262 | 0.030335 | 1.186334 | 0.106796 | 0.096326 | 0.009709 | 0.009709 | 2257.488 | 137.5417 | 14 |
| LQHPT | 7.5 | 0.9614 | 0.956689 | 1.083565 | 0.02557 | 0.969567 | 0.116505 | 0.103941 | 0.009709 | 0.009709 | 2212.114 | 92.16805 | 10 |
| L | 8 | 0.8055 | 0.800524 | 2.021658 | 0.06969 | 0.732251 | 0.116505 | 0.103941 | 0.009709 | 0.009709 | 2560.496 | 440.5499 | 3 |
| Q | 8 | 0.8962 | 0.895136 | 2.264758 | 0.050764 | 1.615471 | 0.106796 | 0.096326 | 0.009709 | 0.009709 | 2437.332 | 317.3864 | 3 |
| H | 8 | 0.9565 | 0.952995 | 0.934122 | 0.02773 | 0.773509 | 0.106796 | 0.096326 | 0.009709 | 0.009709 | 2447.016 | 327.07 | 34 |
| P | 8 | 0.7556 | 0.754928 | 6.077891 | 0.117428 | 2.23012 | 0.106796 | 0.096326 | 0.009709 | 0.009709 | 2553.338 | 433.3919 | 3 |
| T | 8 | 0.9124 | 0.901445 | 0.831498 | 0.023047 | 0.78059 | 0.106796 | 0.096326 | 0.009709 | 0.009709 | 2411.08 | 291.1338 | 6 |
| LQ | 8 | 0.9454 | 0.944184 | 1.175052 | 0.028677 | 0.998047 | 0.106796 | 0.096326 | 0.009709 | 0.009709 | 2315.841 | 195.8953 | 6 |
| LH | 8 | 0.939 | 0.932701 | 0.975207 | 0.029163 | 0.814905 | 0.116505 | 0.103941 | 0.009709 | 0.009709 | 2499.533 | 379.5875 | 41 |
| LP | 8 | 0.8838 | 0.881658 | 1.362063 | 0.05381 | 0.5686 | 0.106796 | 0.096326 | 0.009709 | 0.009709 | 2447.634 | 327.6876 | 6 |
| LT | 8 | 0.914 | 0.9002 | 1.120989 | 0.043397 | 0.784296 | 0.135922 | 0.118599 | 0.009709 | 0.009709 | 2423.903 | 303.9568 | 10 |
| QH | 8 | 0.9503 | 0.948804 | 1.256148 | 0.028544 | 1.078476 | 0.106796 | 0.096326 | 0.009709 | 0.009709 | 2287.989 | 168.0433 | 13 |
| QP | 8 | 0.9099 | 0.907397 | 2.31575 | 0.0496 | 1.788841 | 0.106796 | 0.096326 | 0.009709 | 0.009709 | 2413.61 | 293.664 | 6 |
| QT | 8 | 0.9526 | 0.946971 | 1.335817 | 0.030978 | 1.151759 | 0.116505 | 0.103941 | 0.009709 | 0.009709 | 2259.386 | 139.4405 | 8 |
| HP | 8 | 0.9492 | 0.945999 | 0.913199 | 0.025301 | 0.778345 | 0.106796 | 0.096326 | 0.009709 | 0.009709 | 2377.046 | 257.1005 | 27 |
| HT | 8 | 0.9545 | 0.944914 | 1.432431 | 0.034435 | 1.230688 | 0.106796 | 0.096326 | 0.009709 | 0.009709 | 2387.423 | 267.4775 | 25 |
| PT | 8 | 0.9316 | 0.91815 | 1.388258 | 0.043497 | 1.050997 | 0.145631 | 0.125642 | 0.009709 | 0.009709 | 2365.811 | 245.8653 | 10 |
| LQH | 8 | 0.951 | 0.948398 | 1.120087 | 0.027166 | 0.961223 | 0.106796 | 0.096326 | 0.009709 | 0.009709 | 2305.055 | 185.1091 | 14 |
| LQP | 8 | 0.9443 | 0.942526 | 1.190387 | 0.030758 | 0.990171 | 0.106796 | 0.096326 | 0.009709 | 0.009709 | 2298.137 | 178.1916 | 8 |
| LQT | 8 | 0.9542 | 0.950295 | 1.141375 | 0.027175 | 0.99304 | 0.106796 | 0.096326 | 0.009709 | 0.009709 | 2271.867 | 151.9215 | 8 |
| LHP | 8 | 0.9636 | 0.961438 | 0.618171 | 0.019571 | 0.545032 | 0.106796 | 0.096326 | 0.009709 | 0.009709 | 2288.74 | 168.7942 | 21 |
| LHT | 8 | 0.9505 | 0.939458 | 1.390894 | 0.03514 | 1.199129 | 0.116505 | 0.103941 | 0.009709 | 0.009709 | 2501.21 | 381.2643 | 46 |
| LPT | 8 | 0.9675 | 0.955941 | 1.097244 | 0.026748 | 1.013236 | 0.126214 | 0.111365 | 0.009709 | 0.009709 | 2246.675 | 126.7293 | 12 |
| QHP | 8 | 0.9488 | 0.947567 | 1.288006 | 0.029449 | 1.104595 | 0.106796 | 0.096326 | 0.009709 | 0.009709 | 2286.107 | 166.161 | 14 |
| QHT | 8 | 0.9534 | 0.948399 | 1.324885 | 0.030294 | 1.148533 | 0.106796 | 0.096326 | 0.009709 | 0.009709 | 2265.422 | 145.476 | 14 |
| QPT | 8 | 0.953 | 0.947957 | 1.395658 | 0.030768 | 1.215601 | 0.106796 | 0.096326 | 0.009709 | 0.009709 | 2254.676 | 134.7306 | 9 |
| HPT | 8 | 0.9562 | 0.946606 | 1.327814 | 0.029308 | 1.201692 | 0.106796 | 0.096326 | 0.009709 | 0.009709 | 2397.028 | 277.0825 | 39 |
| LQHP | 8 | 0.9553 | 0.952733 | 1.081563 | 0.026083 | 0.947402 | 0.116505 | 0.103941 | 0.009709 | 0.009709 | 2268.59 | 148.6437 | 16 |
| LQHT | 8 | 0.9547 | 0.950887 | 1.166802 | 0.027379 | 1.018232 | 0.116505 | 0.103941 | 0.009709 | 0.009709 | 2268.762 | 148.8162 | 11 |
| LQPT | 8 | 0.96 | 0.955639 | 1.092634 | 0.025906 | 0.972254 | 0.116505 | 0.103941 | 0.009709 | 0.009709 | 2218.233 | 98.28745 | 9 |
| LHPT | 8 | 0.9679 | 0.96011 | 0.93446 | 0.023828 | 0.857103 | 0.126214 | 0.111365 | 0.009709 | 0.009709 | 2245.432 | 125.4861 | 17 |
| QHPT | 8 | 0.9526 | 0.948183 | 1.370286 | 0.03048 | 1.1931 | 0.106796 | 0.096326 | 0.009709 | 0.009709 | 2261.332 | 141.3865 | 14 |
| LQHPT | 8 | 0.9603 | 0.955855 | 1.088528 | 0.02581 | 0.969744 | 0.116505 | 0.103941 | 0.009709 | 0.009709 | 2217.113 | 97.16678 | 9 |
| L | 8.5 | 0.8053 | 0.800278 | 2.057402 | 0.070338 | 0.745662 | 0.116505 | 0.103941 | 0.009709 | 0.009709 | 2560.612 | 440.6666 | 3 |
| Q | 8.5 | 0.8962 | 0.895095 | 2.264012 | 0.050756 | 1.614752 | 0.106796 | 0.096326 | 0.009709 | 0.009709 | 2437.455 | 317.5094 | 3 |
| H | 8.5 | 0.952 | 0.947293 | 0.936899 | 0.030695 | 0.744078 | 0.106796 | 0.096326 | 0.009709 | 0.009709 | 2443.916 | 323.9705 | 28 |
| P | 8.5 | 0.7556 | 0.755021 | 6.069273 | 0.117345 | 2.226391 | 0.106796 | 0.096326 | 0.009709 | 0.009709 | 2553.381 | 433.4355 | 3 |
| T | 8.5 | 0.9124 | 0.902135 | 0.829859 | 0.022465 | 0.779733 | 0.097087 | 0.088521 | 0.009709 | 0.009709 | 2416.236 | 296.29 | 6 |
| LQ | 8.5 | 0.9453 | 0.944113 | 1.176347 | 0.028718 | 0.998838 | 0.106796 | 0.096326 | 0.009709 | 0.009709 | 2316.459 | 196.5129 | 6 |
| LH | 8.5 | 0.9318 | 0.924365 | 0.951367 | 0.029904 | 0.776491 | 0.116505 | 0.103941 | 0.009709 | 0.009709 | 2516.888 | 396.9418 | 41 |
| LP | 8.5 | 0.8833 | 0.88115 | 1.378171 | 0.054182 | 0.572809 | 0.106796 | 0.096326 | 0.009709 | 0.009709 | 2445.784 | 325.8383 | 5 |
| LT | 8.5 | 0.9115 | 0.898184 | 1.110747 | 0.044035 | 0.759671 | 0.135922 | 0.118599 | 0.009709 | 0.009709 | 2428.648 | 308.7018 | 9 |
| QH | 8.5 | 0.9494 | 0.947834 | 1.271981 | 0.029076 | 1.086307 | 0.106796 | 0.096326 | 0.009709 | 0.009709 | 2299.565 | 179.6193 | 15 |
| QP | 8.5 | 0.9098 | 0.907294 | 2.313759 | 0.049545 | 1.787718 | 0.106796 | 0.096326 | 0.009709 | 0.009709 | 2413.832 | 293.8865 | 6 |
| QT | 8.5 | 0.9515 | 0.946201 | 1.338167 | 0.031205 | 1.146363 | 0.126214 | 0.111365 | 0.009709 | 0.009709 | 2262.992 | 143.0456 | 7 |
| HP | 8.5 | 0.9461 | 0.942584 | 0.926929 | 0.026059 | 0.775792 | 0.106796 | 0.096326 | 0.009709 | 0.009709 | 2380.532 | 260.5862 | 24 |
| HT | 8.5 | 0.949 | 0.93946 | 1.431498 | 0.036443 | 1.197506 | 0.106796 | 0.096326 | 0.009709 | 0.009709 | 2377.329 | 257.3836 | 15 |
| PT | 8.5 | 0.9284 | 0.915195 | 1.42483 | 0.045076 | 1.057342 | 0.145631 | 0.125642 | 0.009709 | 0.009709 | 2373.393 | 253.4469 | 10 |
| LQH | 8.5 | 0.9496 | 0.9477 | 1.125125 | 0.027408 | 0.963974 | 0.106796 | 0.096326 | 0.009709 | 0.009709 | 2307.801 | 187.8549 | 12 |
| LQP | 8.5 | 0.9439 | 0.942056 | 1.207579 | 0.031019 | 1.003891 | 0.106796 | 0.096326 | 0.009709 | 0.009709 | 2299.957 | 180.0111 | 8 |
| LQT | 8.5 | 0.9527 | 0.949783 | 1.138548 | 0.027141 | 0.986645 | 0.106796 | 0.096326 | 0.009709 | 0.009709 | 2275.578 | 155.6318 | 7 |
| LHP | 8.5 | 0.9624 | 0.9599 | 0.638249 | 0.020023 | 0.562125 | 0.106796 | 0.096326 | 0.009709 | 0.009709 | 2289.529 | 169.5835 | 18 |
| LHT | 8.5 | 0.9471 | 0.932874 | 1.311194 | 0.035707 | 1.133381 | 0.116505 | 0.103941 | 0.009709 | 0.009709 | 2471.301 | 351.3549 | 38 |
| LPT | 8.5 | 0.9666 | 0.955 | 1.093429 | 0.026886 | 1.008131 | 0.126214 | 0.111365 | 0.009709 | 0.009709 | 2254.635 | 134.6889 | 11 |
| QHP | 8.5 | 0.9485 | 0.947231 | 1.302353 | 0.029696 | 1.115906 | 0.106796 | 0.096326 | 0.009709 | 0.009709 | 2286.823 | 166.8774 | 13 |
| QHT | 8.5 | 0.9525 | 0.947629 | 1.327452 | 0.030602 | 1.144612 | 0.106796 | 0.096326 | 0.009709 | 0.009709 | 2268.662 | 148.7162 | 13 |
| QPT | 8.5 | 0.9527 | 0.947651 | 1.410952 | 0.030983 | 1.228184 | 0.106796 | 0.096326 | 0.009709 | 0.009709 | 2259.445 | 139.4992 | 9 |
| HPT | 8.5 | 0.9536 | 0.943719 | 1.317175 | 0.02949 | 1.186732 | 0.116505 | 0.103941 | 0.009709 | 0.009709 | 2395.83 | 275.8846 | 36 |
| LQHP | 8.5 | 0.9536 | 0.95112 | 1.103648 | 0.026791 | 0.959988 | 0.116505 | 0.103941 | 0.009709 | 0.009709 | 2269.841 | 149.8947 | 13 |
| LQHT | 8.5 | 0.9532 | 0.949578 | 1.158016 | 0.027686 | 1.002763 | 0.116505 | 0.103941 | 0.009709 | 0.009709 | 2277.155 | 157.2093 | 11 |
| LQPT | 8.5 | 0.9588 | 0.954643 | 1.103749 | 0.026197 | 0.978222 | 0.116505 | 0.103941 | 0.009709 | 0.009709 | 2225.64 | 105.6937 | 9 |
| LHPT | 8.5 | 0.9675 | 0.959514 | 0.943518 | 0.024045 | 0.865124 | 0.126214 | 0.111365 | 0.009709 | 0.009709 | 2256.713 | 136.7674 | 18 |
| QHPT | 8.5 | 0.9522 | 0.947849 | 1.381152 | 0.030652 | 1.201638 | 0.106796 | 0.096326 | 0.009709 | 0.009709 | 2265.205 | 145.2587 | 14 |
| LQHPT | 8.5 | 0.9592 | 0.954925 | 1.101143 | 0.02615 | 0.976798 | 0.116505 | 0.103941 | 0.009709 | 0.009709 | 2224.107 | 104.1614 | 9 |
| L | 9 | 0.8051 | 0.800098 | 2.091498 | 0.070934 | 0.757969 | 0.106796 | 0.096326 | 0.009709 | 0.009709 | 2560.737 | 440.7908 | 3 |
| Q | 9 | 0.8961 | 0.895073 | 2.262484 | 0.050731 | 1.613433 | 0.116505 | 0.103941 | 0.009709 | 0.009709 | 2437.585 | 317.6391 | 3 |
| H | 9 | 0.9455 | 0.939325 | 0.913739 | 0.032288 | 0.697418 | 0.106796 | 0.096326 | 0.009709 | 0.009709 | 2442.949 | 323.0033 | 21 |
| P | 9 | 0.7557 | 0.755092 | 6.062269 | 0.117289 | 2.22359 | 0.106796 | 0.096326 | 0.009709 | 0.009709 | 2553.428 | 433.4816 | 3 |
| T | 9 | 0.9124 | 0.902135 | 0.829859 | 0.022465 | 0.779733 | 0.097087 | 0.088521 | 0.009709 | 0.009709 | 2421.487 | 301.5412 | 6 |
| LQ | 9 | 0.9452 | 0.944046 | 1.178384 | 0.028764 | 1.000342 | 0.106796 | 0.096326 | 0.009709 | 0.009709 | 2317.073 | 197.1272 | 6 |
| LH | 9 | 0.9236 | 0.914977 | 0.913567 | 0.03183 | 0.706984 | 0.106796 | 0.096326 | 0.009709 | 0.009709 | 2445.818 | 325.8723 | 20 |
| LP | 9 | 0.8827 | 0.880539 | 1.396405 | 0.054626 | 0.578676 | 0.106796 | 0.096326 | 0.009709 | 0.009709 | 2446.215 | 326.2688 | 5 |
| LT | 9 | 0.9093 | 0.896425 | 1.096503 | 0.044509 | 0.73629 | 0.135922 | 0.118599 | 0.009709 | 0.009709 | 2435.645 | 315.6996 | 9 |
| QH | 9 | 0.9484 | 0.946663 | 1.286445 | 0.029652 | 1.092107 | 0.106796 | 0.096326 | 0.009709 | 0.009709 | 2308.114 | 188.168 | 16 |
| QP | 9 | 0.9098 | 0.907219 | 2.311025 | 0.049469 | 1.786142 | 0.106796 | 0.096326 | 0.009709 | 0.009709 | 2414.063 | 294.1171 | 6 |
| QT | 9 | 0.9505 | 0.945247 | 1.345963 | 0.031598 | 1.146001 | 0.116505 | 0.103941 | 0.009709 | 0.009709 | 2267.607 | 147.6614 | 7 |
| HP | 9 | 0.9422 | 0.938199 | 0.930143 | 0.02783 | 0.751676 | 0.106796 | 0.096326 | 0.009709 | 0.009709 | 2395.204 | 275.2586 | 24 |
| HT | 9 | 0.9452 | 0.934919 | 1.395365 | 0.037834 | 1.154212 | 0.106796 | 0.096326 | 0.009709 | 0.009709 | 2390.734 | 270.7884 | 13 |
| PT | 9 | 0.9256 | 0.912687 | 1.458011 | 0.046336 | 1.062792 | 0.145631 | 0.125642 | 0.009709 | 0.009709 | 2377.534 | 257.5886 | 9 |
| LQH | 9 | 0.9492 | 0.947196 | 1.132508 | 0.027591 | 0.969926 | 0.106796 | 0.096326 | 0.009709 | 0.009709 | 2310.08 | 190.1336 | 11 |
| LQP | 9 | 0.9435 | 0.941687 | 1.217183 | 0.03118 | 1.011261 | 0.106796 | 0.096326 | 0.009709 | 0.009709 | 2301.767 | 181.8209 | 8 |
| LQT | 9 | 0.9522 | 0.949378 | 1.139054 | 0.027263 | 0.984669 | 0.106796 | 0.096326 | 0.009709 | 0.009709 | 2278.927 | 158.9808 | 7 |
| LHP | 9 | 0.9608 | 0.957938 | 0.648894 | 0.020462 | 0.570321 | 0.106796 | 0.096326 | 0.009709 | 0.009709 | 2304.172 | 184.2257 | 19 |
| LHT | 9 | 0.9395 | 0.925244 | 1.140388 | 0.03549 | 0.960091 | 0.116505 | 0.103941 | 0.009709 | 0.009709 | 2439.355 | 319.4093 | 27 |
| LPT | 9 | 0.9654 | 0.953835 | 1.08305 | 0.027026 | 0.996645 | 0.126214 | 0.111365 | 0.009709 | 0.009709 | 2265.184 | 145.2377 | 11 |
| QHP | 9 | 0.9483 | 0.946897 | 1.317318 | 0.029967 | 1.127801 | 0.106796 | 0.096326 | 0.009709 | 0.009709 | 2295.716 | 175.7701 | 15 |
| QHT | 9 | 0.9514 | 0.946827 | 1.327543 | 0.030878 | 1.137677 | 0.106796 | 0.096326 | 0.009709 | 0.009709 | 2267.095 | 147.1487 | 10 |
| QPT | 9 | 0.9525 | 0.947388 | 1.422464 | 0.031156 | 1.237189 | 0.106796 | 0.096326 | 0.009709 | 0.009709 | 2261.875 | 141.9291 | 8 |
| HPT | 9 | 0.9501 | 0.940404 | 1.31543 | 0.029951 | 1.171607 | 0.116505 | 0.103941 | 0.009709 | 0.009709 | 2368.798 | 248.8516 | 26 |
| LQHP | 9 | 0.9521 | 0.949449 | 1.123604 | 0.027521 | 0.971224 | 0.106796 | 0.096326 | 0.009709 | 0.009709 | 2283.554 | 163.6082 | 15 |
| LQHT | 9 | 0.9517 | 0.948906 | 1.151474 | 0.027685 | 0.992992 | 0.116505 | 0.103941 | 0.009709 | 0.009709 | 2279.654 | 159.7079 | 9 |
| LQPT | 9 | 0.9581 | 0.954219 | 1.108656 | 0.02634 | 0.979358 | 0.116505 | 0.103941 | 0.009709 | 0.009709 | 2228.539 | 108.5936 | 8 |
| LHPT | 9 | 0.9671 | 0.958733 | 0.953378 | 0.024384 | 0.87317 | 0.126214 | 0.111365 | 0.009709 | 0.009709 | 2254.583 | 134.6374 | 14 |
| QHPT | 9 | 0.9519 | 0.947416 | 1.398207 | 0.030969 | 1.215587 | 0.106796 | 0.096326 | 0.009709 | 0.009709 | 2266.447 | 146.5012 | 13 |
| LQHPT | 9 | 0.9581 | 0.954222 | 1.108811 | 0.026337 | 0.979673 | 0.116505 | 0.103941 | 0.009709 | 0.009709 | 2231.081 | 111.1351 | 9 |
| L | 9.5 | 0.8048 | 0.799871 | 2.124942 | 0.071526 | 0.77021 | 0.116505 | 0.103941 | 0.009709 | 0.009709 | 2560.868 | 440.9224 | 3 |
| Q | 9.5 | 0.8961 | 0.895037 | 2.261132 | 0.050719 | 1.612214 | 0.116505 | 0.103941 | 0.009709 | 0.009709 | 2437.721 | 317.7752 | 3 |
| H | 9.5 | 0.9357 | 0.925042 | 0.787316 | 0.029621 | 0.632094 | 0.106796 | 0.096326 | 0.009709 | 0.009709 | 2441.964 | 322.0181 | 14 |
| P | 9.5 | 0.7558 | 0.755181 | 6.054587 | 0.117204 | 2.221049 | 0.106796 | 0.096326 | 0.009709 | 0.009709 | 2553.476 | 433.5304 | 3 |
| T | 9.5 | 0.9124 | 0.902135 | 0.829859 | 0.022465 | 0.779733 | 0.097087 | 0.088521 | 0.009709 | 0.009709 | 2426.931 | 306.9855 | 6 |
| LQ | 9.5 | 0.9452 | 0.944003 | 1.179877 | 0.0288 | 1.001273 | 0.106796 | 0.096326 | 0.009709 | 0.009709 | 2315.434 | 195.4884 | 5 |
| LH | 9.5 | 0.913 | 0.905285 | 0.925532 | 0.035134 | 0.652169 | 0.126214 | 0.111365 | 0.009709 | 0.009709 | 2450.979 | 331.0332 | 15 |
| LP | 9.5 | 0.8821 | 0.87991 | 1.414871 | 0.055062 | 0.584664 | 0.106796 | 0.096326 | 0.009709 | 0.009709 | 2446.667 | 326.7215 | 5 |
| LT | 9.5 | 0.9079 | 0.89555 | 1.096394 | 0.045045 | 0.72315 | 0.135922 | 0.118599 | 0.009709 | 0.009709 | 2439.498 | 319.5521 | 8 |
| QH | 9.5 | 0.9471 | 0.945214 | 1.30496 | 0.030383 | 1.099685 | 0.106796 | 0.096326 | 0.009709 | 0.009709 | 2320.486 | 200.5405 | 18 |
| QP | 9.5 | 0.9097 | 0.907089 | 2.309897 | 0.049429 | 1.785807 | 0.106796 | 0.096326 | 0.009709 | 0.009709 | 2414.311 | 294.3655 | 6 |
| QT | 9.5 | 0.9493 | 0.944259 | 1.355081 | 0.031992 | 1.147806 | 0.106796 | 0.096326 | 0.009709 | 0.009709 | 2272.325 | 152.3787 | 7 |
| HP | 9.5 | 0.9373 | 0.932652 | 0.948493 | 0.030972 | 0.724569 | 0.106796 | 0.096326 | 0.009709 | 0.009709 | 2391.08 | 271.1337 | 18 |
| HT | 9.5 | 0.9393 | 0.927731 | 1.320445 | 0.037738 | 1.085649 | 0.116505 | 0.103941 | 0.009709 | 0.009709 | 2404.688 | 284.7426 | 11 |
| PT | 9.5 | 0.9228 | 0.910284 | 1.486906 | 0.047478 | 1.06395 | 0.145631 | 0.125642 | 0.009709 | 0.009709 | 2384.144 | 264.1981 | 9 |
| LQH | 9.5 | 0.9487 | 0.946781 | 1.137994 | 0.027698 | 0.974034 | 0.106796 | 0.096326 | 0.009709 | 0.009709 | 2312.035 | 192.0887 | 10 |
| LQP | 9.5 | 0.9432 | 0.941317 | 1.231743 | 0.031386 | 1.023013 | 0.106796 | 0.096326 | 0.009709 | 0.009709 | 2303.221 | 183.2753 | 8 |
| LQT | 9.5 | 0.9516 | 0.948964 | 1.138868 | 0.027382 | 0.982013 | 0.116505 | 0.103941 | 0.009709 | 0.009709 | 2282.374 | 162.428 | 7 |
| LHP | 9.5 | 0.959 | 0.955988 | 0.66561 | 0.020911 | 0.581376 | 0.106796 | 0.096326 | 0.009709 | 0.009709 | 2309.554 | 189.6081 | 17 |
| LHT | 9.5 | 0.9295 | 0.915729 | 1.062824 | 0.036758 | 0.848396 | 0.116505 | 0.103941 | 0.009709 | 0.009709 | 2409.856 | 289.9105 | 12 |
| LPT | 9.5 | 0.964 | 0.95281 | 1.068333 | 0.027037 | 0.980045 | 0.126214 | 0.111365 | 0.009709 | 0.009709 | 2273.14 | 153.1937 | 10 |
| QHP | 9.5 | 0.948 | 0.946673 | 1.329976 | 0.030145 | 1.137528 | 0.106796 | 0.096326 | 0.009709 | 0.009709 | 2296.342 | 176.3964 | 14 |
| QHT | 9.5 | 0.9504 | 0.946057 | 1.325185 | 0.03106 | 1.129 | 0.116505 | 0.103941 | 0.009709 | 0.009709 | 2270.82 | 150.874 | 9 |
| QPT | 9.5 | 0.9522 | 0.94712 | 1.435312 | 0.031327 | 1.247116 | 0.106796 | 0.096326 | 0.009709 | 0.009709 | 2266.859 | 146.9131 | 8 |
| HPT | 9.5 | 0.9463 | 0.93608 | 1.317694 | 0.031505 | 1.148361 | 0.126214 | 0.111365 | 0.009709 | 0.009709 | 2361.291 | 241.3455 | 19 |
| LQHP | 9.5 | 0.9504 | 0.94804 | 1.14248 | 0.02808 | 0.982412 | 0.106796 | 0.096326 | 0.009709 | 0.009709 | 2291.997 | 172.0507 | 15 |
| LQHT | 9.5 | 0.9513 | 0.948572 | 1.14904 | 0.027725 | 0.9893 | 0.116505 | 0.103941 | 0.009709 | 0.009709 | 2284.53 | 164.5836 | 9 |
| LQPT | 9.5 | 0.9574 | 0.953747 | 1.114909 | 0.026484 | 0.982401 | 0.116505 | 0.103941 | 0.009709 | 0.009709 | 2233.506 | 113.5604 | 8 |
| LHPT | 9.5 | 0.9664 | 0.957716 | 0.974855 | 0.024828 | 0.892427 | 0.116505 | 0.103941 | 0.009709 | 0.009709 | 2272.241 | 152.2955 | 17 |
| QHPT | 9.5 | 0.9516 | 0.947166 | 1.406668 | 0.031114 | 1.221683 | 0.106796 | 0.096326 | 0.009709 | 0.009709 | 2267.676 | 147.7302 | 12 |
| LQHPT | 9.5 | 0.9574 | 0.953768 | 1.114632 | 0.026465 | 0.98225 | 0.116505 | 0.103941 | 0.009709 | 0.009709 | 2233.659 | 113.7134 | 8 |
| L | 10 | 0.8045 | 0.799586 | 2.157855 | 0.07212 | 0.782735 | 0.106796 | 0.096326 | 0.009709 | 0.009709 | 2561.007 | 441.0613 | 3 |
| Q | 10 | 0.896 | 0.894976 | 2.260001 | 0.05073 | 1.611047 | 0.116505 | 0.103941 | 0.009709 | 0.009709 | 2437.864 | 317.9177 | 3 |
| H | 10 | 0.9219 | 0.920387 | 0.726745 | 0.024857 | 0.569003 | 0.106796 | 0.096326 | 0.009709 | 0.009709 | 2444.089 | 324.1434 | 10 |
| P | 10 | 0.7559 | 0.755247 | 6.048515 | 0.117156 | 2.21924 | 0.106796 | 0.096326 | 0.009709 | 0.009709 | 2553.528 | 433.5817 | 3 |
| T | 10 | 0.9124 | 0.902135 | 0.829859 | 0.022465 | 0.779733 | 0.097087 | 0.088521 | 0.009709 | 0.009709 | 2432.563 | 312.617 | 6 |
| LQ | 10 | 0.9451 | 0.943995 | 1.181771 | 0.028829 | 1.002635 | 0.106796 | 0.096326 | 0.009709 | 0.009709 | 2315.89 | 195.9439 | 5 |
| LH | 10 | 0.9019 | 0.893939 | 0.998307 | 0.039521 | 0.629691 | 0.116505 | 0.103941 | 0.009709 | 0.009709 | 2461.184 | 341.2381 | 12 |
| LP | 10 | 0.8815 | 0.879294 | 1.434222 | 0.055498 | 0.591024 | 0.106796 | 0.096326 | 0.009709 | 0.009709 | 2447.142 | 327.1958 | 5 |
| LT | 10 | 0.9068 | 0.894501 | 1.097343 | 0.045594 | 0.711841 | 0.135922 | 0.118599 | 0.009709 | 0.009709 | 2445.347 | 325.4008 | 8 |
| QH | 10 | 0.9457 | 0.943722 | 1.321127 | 0.031138 | 1.103468 | 0.106796 | 0.096326 | 0.009709 | 0.009709 | 2321.351 | 201.405 | 16 |
| QP | 10 | 0.9095 | 0.906981 | 2.306674 | 0.049361 | 1.783412 | 0.106796 | 0.096326 | 0.009709 | 0.009709 | 2414.577 | 294.6307 | 6 |
| QT | 10 | 0.9482 | 0.94373 | 1.362794 | 0.03213 | 1.150128 | 0.106796 | 0.096326 | 0.009709 | 0.009709 | 2274.755 | 154.8091 | 6 |
| HP | 10 | 0.9309 | 0.925934 | 0.987295 | 0.034761 | 0.700077 | 0.135922 | 0.118599 | 0.009709 | 0.009709 | 2396.188 | 276.2419 | 14 |
| HT | 10 | 0.934 | 0.906592 | 1.105815 | 0.036459 | 1.022496 | 0.116505 | 0.103941 | 0.009709 | 0.009709 | 2413.343 | 293.3968 | 8 |
| PT | 10 | 0.9202 | 0.908528 | 1.514613 | 0.048235 | 1.069946 | 0.135922 | 0.118599 | 0.009709 | 0.009709 | 2388.395 | 268.4494 | 8 |
| LQH | 10 | 0.9482 | 0.94643 | 1.143951 | 0.027772 | 0.978251 | 0.106796 | 0.096326 | 0.009709 | 0.009709 | 2312.517 | 192.5715 | 9 |
| LQP | 10 | 0.9429 | 0.941229 | 1.235669 | 0.031436 | 1.025941 | 0.106796 | 0.096326 | 0.009709 | 0.009709 | 2304.65 | 184.704 | 8 |
| LQT | 10 | 0.951 | 0.948643 | 1.138645 | 0.027446 | 0.97973 | 0.116505 | 0.103941 | 0.009709 | 0.009709 | 2285.811 | 165.8647 | 7 |
| LHP | 10 | 0.9569 | 0.953742 | 0.678892 | 0.021507 | 0.587966 | 0.106796 | 0.096326 | 0.009709 | 0.009709 | 2317.402 | 197.4564 | 16 |
| LHT | 10 | 0.9183 | 0.905743 | 1.01419 | 0.039152 | 0.748869 | 0.135922 | 0.118599 | 0.009709 | 0.009709 | 2420.884 | 300.9379 | 9 |
| LPT | 10 | 0.9628 | 0.951898 | 1.052778 | 0.02711 | 0.961061 | 0.126214 | 0.111365 | 0.009709 | 0.009709 | 2279.545 | 159.5993 | 9 |
| QHP | 10 | 0.9478 | 0.946329 | 1.344773 | 0.030408 | 1.149342 | 0.106796 | 0.096326 | 0.009709 | 0.009709 | 2302.607 | 182.6615 | 15 |
| QHT | 10 | 0.9493 | 0.945021 | 1.325559 | 0.031411 | 1.122241 | 0.116505 | 0.103941 | 0.009709 | 0.009709 | 2277.152 | 157.2064 | 9 |
| QPT | 10 | 0.9519 | 0.946877 | 1.447701 | 0.031499 | 1.256495 | 0.106796 | 0.096326 | 0.009709 | 0.009709 | 2267.891 | 147.9453 | 7 |
| HPT | 10 | 0.9416 | 0.930286 | 1.329488 | 0.034883 | 1.115002 | 0.126214 | 0.111365 | 0.009709 | 0.009709 | 2366.357 | 246.4116 | 15 |
| LQHP | 10 | 0.9493 | 0.946954 | 1.153642 | 0.028566 | 0.985853 | 0.116505 | 0.103941 | 0.009709 | 0.009709 | 2295.686 | 175.74 | 14 |
| LQHT | 10 | 0.951 | 0.94851 | 1.142746 | 0.027571 | 0.98278 | 0.116505 | 0.103941 | 0.009709 | 0.009709 | 2284.693 | 164.747 | 7 |
| LQPT | 10 | 0.9567 | 0.953169 | 1.120734 | 0.026706 | 0.984931 | 0.116505 | 0.103941 | 0.009709 | 0.009709 | 2238.697 | 118.7511 | 8 |
| LHPT | 10 | 0.9655 | 0.956564 | 0.980842 | 0.025245 | 0.896143 | 0.116505 | 0.103941 | 0.009709 | 0.009709 | 2282.07 | 162.1241 | 17 |
| QHPT | 10 | 0.9513 | 0.946879 | 1.415514 | 0.03128 | 1.228096 | 0.106796 | 0.096326 | 0.009709 | 0.009709 | 2269.045 | 149.0989 | 11 |
| LQHPT | 10 | 0.9568 | 0.953178 | 1.121292 | 0.026704 | 0.985496 | 0.116505 | 0.103941 | 0.009709 | 0.009709 | 2238.646 | 118.7002 | 8 |
